# Phasic metabolic remodeling coordinates cardiac myofibroblast differentiation and function

**DOI:** 10.1016/j.redox.2026.104441

**Published:** 2026-10-16

**Authors:** Collin K. Wells, Lianay Gutierrez Luque, Daniel C. Nguyen, Madison S. Teer, Ning Chen, Yania Martinez-Ondaro, Pawel K. Lorkiewicz, Richa Singhal, Maleesha De Silva, Bradford G. Hill

**Affiliations:** a Center for Cardiometabolic Science, Christina Lee Brown Envirome Institute, University of Louisville School of Medicine, Louisville, KY, USA; b Department of Biochemistry and Molecular Genetics, University of Louisville School of Medicine, Louisville, KY, USA; c Department of Physiology, University of Louisville, Louisville, KY, USA

**Keywords:** Fibroblast, Glycolysis, Biosynthesis, Mitochondria, Stable isotope, Fibrosis

## Abstract

Fibroblast differentiation into matrix-producing, contractile myofibroblasts is accompanied by metabolic reprogramming, but whether these changes precede differentiation or arise from the activated phenotype remains unclear. We integrated time-resolved extracellular flux analysis, transcriptomics, stable isotope-resolved metabolomics, and metabolic interventions to define the relationship between metabolism and myofibroblast differentiation in adult mouse cardiac fibroblasts stimulated with transforming growth factor-β (TGFβ). TGFβ increased glycolysis within 1.5 h, before broad transcriptional remodeling and induction of periostin and α-smooth muscle actin. Mitochondrial respiration initially declined but increased at 48 h as the phenotype emerged, accompanied by smaller, less elongated mitochondria and greater respiratory capacity. [U-^13^C_6_]-glucose tracing revealed progressive incorporation of glucose-derived carbon into glycolytic intermediates, nucleotide sugars, and glycerol 3-phosphate, followed later by increased labeling of tricarboxylic acid cycle intermediates. TGFβ-treated fibroblasts also demonstrated enhanced insulin-stimulated glycolysis. Replacing glucose with galactose attenuated collagen and periostin induction, whereas glyceraldehyde 3-phosphate dehydrogenase inhibition prevented induction of collagen, periostin, and α-smooth muscle actin. Conversely, glucose transporter 1 or mutant PFK2 (Glyco^Hi^) overexpression increased glycolysis without inducing myofibroblast markers. Together, these loss- and gain-of-function studies indicate that glycolytic activity is required for complete differentiation but is not sufficient to initiate the phenotype. Inhibition of mitochondrial Complex I or ATP synthase preferentially reduced collagen abundance, while glycolytic and Complex I inhibition diminished TGFβ-induced collagen gel contraction. These findings define a phasic metabolic program in which early glycolytic activation precedes transcriptional and phenotypic remodeling, whereas mitochondrial respiratory capacity increases later and distinct metabolic nodes selectively regulate collagen abundance and contractile behavior.

## Highlights

• TGFβ activates glycolysis before broad transcriptional remodeling.
• Glucose carbon is later redirected into biosynthetic and Krebs cycle pools.
• Activated glycolysis is required but insufficient for complete myofibroblast conversion.
• Myofibroblasts have smaller mitochondria with greater respiratory capacity.
• Metabolic nodes selectively control collagen accumulation and contraction.

## Graphical abstract

**Figure:**
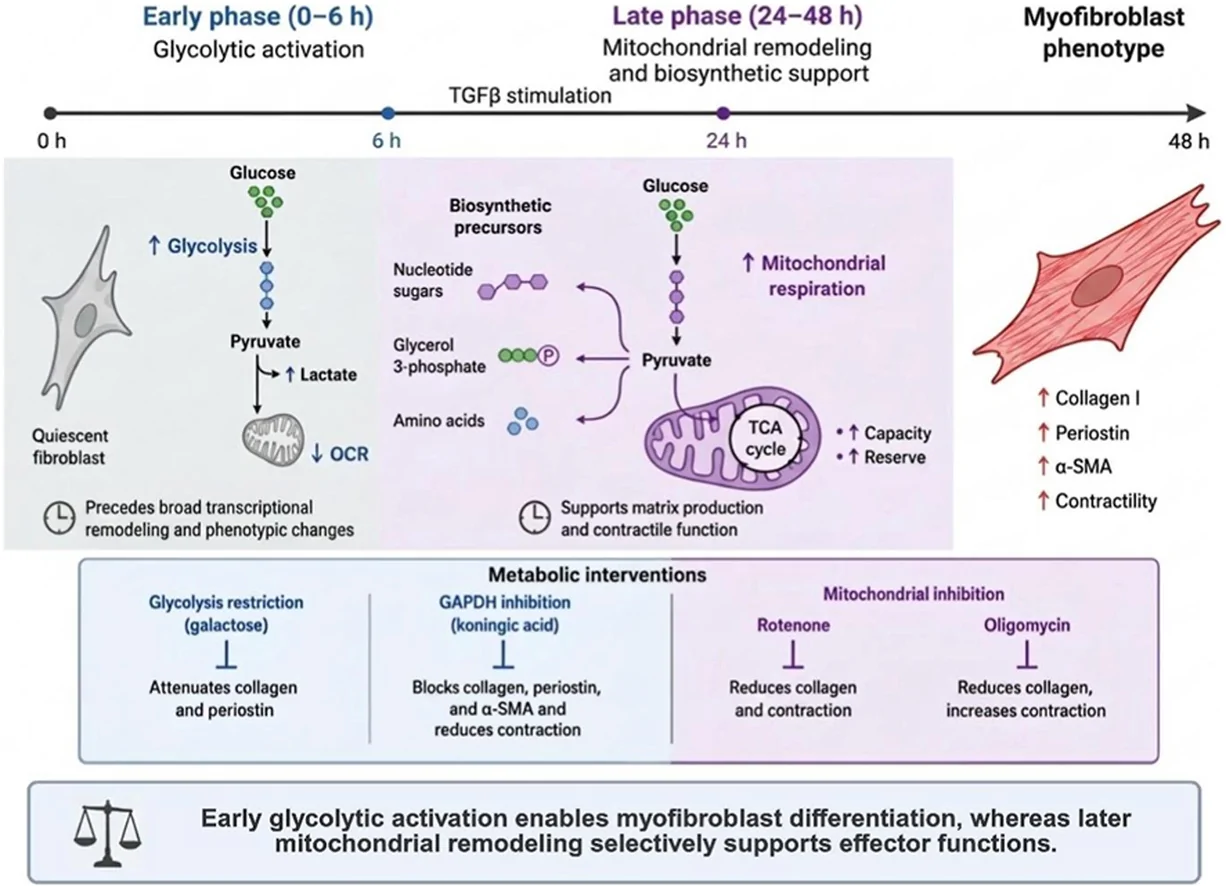

## 1 Introduction

Fibroblasts are highly plastic mesenchymal cells that play essential roles in tissue homeostasis and wound repair. Following tissue injury, fibroblasts differentiate into myofibroblasts, a process characterized by robust extracellular matrix (ECM) synthesis and the upregulation of contractile machinery that promotes wound closure. Myofibroblasts are among the most biosynthetically active cells in injured tissues, capable of producing extraordinarily large quantities of collagen as well as polysaccharides such as hyaluronan [1], [2], [3], [4]. Together with increased cytoskeletal remodeling and force generation, these processes impose substantial energetic and biosynthetic demands, suggesting that coordinated metabolic remodeling may be required to support myofibroblast differentiation. Defining the metabolic basis of this transition could reveal new therapeutic strategies to limit pathological fibrosis.

During tissue repair and remodeling, fibroblast activation is driven by a diverse array of signaling ligands that shape fibroblast phenotype and function. Acquisition of activated or myofibroblast phenotypes requires not only increased energy production but also enhanced anabolic capacity. Consistent with this concept, prior studies have demonstrated that perturbation of core metabolic pathways influences fibrotic responses. In particular, glucose and glutamine metabolism have emerged as critical regulators of myofibroblast differentiation. Inhibition of glycolysis, either at glucose entry or at the phosphofructokinase step, appears to attenuate fibroblast activation [5], [6], [7], [8], [9], [10]. Beyond ATP production, glycolytic intermediates directly contribute to ECM biosynthesis: glucose-6-phosphate and fructose-6-phosphate serve as precursors for UDP-sugars required for hyaluronan synthesis [11], while 3-phosphoglycerate can supply carbon for the production of glycine, which is the most abundant amino acid in collagen [12], [13], [14], [15]. Furthermore, recent studies implicate glutaminolysis in both promoting and sustaining the activated cardiac fibroblast phenotype [16], [17]. Collectively, these findings support a close link between metabolic remodeling and myofibroblast differentiation; however, when metabolic changes occur relative to activating signals, and whether they are necessary or sufficient to drive fibrotic responses, remain poorly defined.

Accordingly, the goal of this study was to define the temporal nature and functional significance of metabolic remodeling induced by the prototypical profibrotic cytokine transforming growth factor-β (TGFβ). By integrating extracellular flux analysis and stable isotope-resolved metabolomics with transcriptomic profiling and established markers of myofibroblast differentiation, we demonstrate that glycolytic activation occurs early following TGFβ stimulation and is required, but not sufficient, for complete myofibroblast differentiation. Moreover, we show that the phenotypic consequences of metabolic perturbation depend on the glycolytic process targeted, with restriction of glucose-supported glycolysis and inhibition of glyceraldehyde 3-phosphate dehydrogenase (GAPDH)-dependent central glycolytic flux exerting distinct effects on the induction of individual myofibroblast markers and on collagen abundance. Mitochondrial activity likewise exerts selective control over downstream effector functions, particularly collagen abundance and matrix contraction, without uniformly regulating all features of the differentiated phenotype. Together, these findings establish a temporal and functional framework for metabolic remodeling during fibroblast activation and identify pathway- and node-specific metabolic checkpoints that may be leveraged to selectively modulate fibrotic responses.

## 2 Results

*TGFβ induces an early glycolytic response that precedes myofibroblast formation:* Myofibroblast differentiation involves significant metabolic changes, including increased glucose catabolism and oxidative phosphorylation [18], [19]. To understand the temporal nature of these metabolic shifts in relation to myofibroblast differentiation, we first assessed when the myofibroblast phenotype appears by treating murine adult cardiac fibroblasts with TGFβ for 6–48 h. Basic fibroblast growth factor (bFGF) was used as an anti-myofibroblastic comparator rather than a ligand-free control. This approach is based on previous studies showing that bFGF maintains a fibroblast-like phenotype and opposes TGFβ-induced myofibroblast differentiation in several mesenchymal cell types, including human cardiac fibroblasts [20], [21], [22], [23]. As shown in Fig. 1A–C, the myofibroblast markers periostin (POSTN) and α-smooth muscle actin (αSMA) were significantly elevated 48 h after TGFβ exposure, but were not significantly elevated at earlier times. Because TGFβ-induced myofibroblast marker responses were broadly similar in fibroblasts derived from male and female hearts (Suppl. Fig. 1), and recent single-nucleus transcriptomic analysis of post-MI hearts demonstrated largely conserved temporal fibroblast responses across sexes [24], subsequent mechanistic studies were performed primarily in male-derived fibroblasts.

**Fig. 1:**
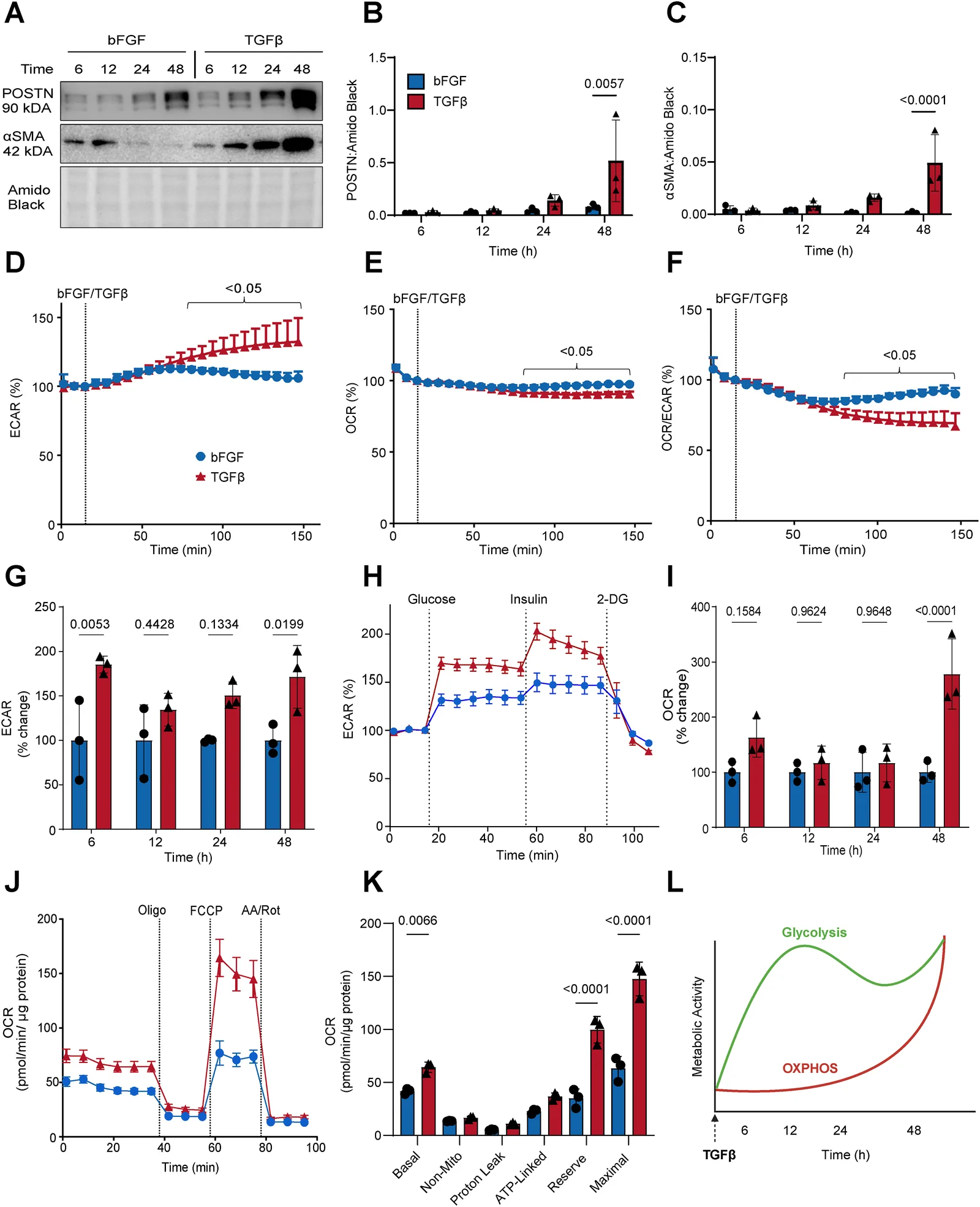
**TGFβ induces an early glycolytic response that precedes acquisition of the myofibroblast phenotype.** Naïve cardiac fibroblasts isolated from adult male mice were treated with ligand, i.e. TGFβ (20 ng/ml) or bFGF (control; 20 ng/ml), followed by assessment of myofibroblast markers and catabolic rates. (**A**) Representative immunoblots of myofibroblast markers, periostin (POSTN) and α-smooth muscle actin (αSMA), after 6–48 h of incubation with ligand. (**B, C**) Quantification of POSTN and αSMA. (**D**) Glycolytic rates, as assessed by the extracellular acidification rate (ECAR), after acute ligand treatment. (**E**) Respiratory activity, as assessed by the oxygen consumption rate (OCR), after acute ligand treatment. (**F**) Acute changes in cell reliance on mitochondrial respiration versus glycolysis after acute ligand treatment, as indicated by the OCR/ECAR ratio. (**G**) Time-dependent analysis of ECAR after treatment with ligands for 6–48 h. (**H**) Glycolysis stress test in which fibroblasts treated with ligand for 48 h were subjected to sequential injections of glucose (10 mM), insulin (100 nM), and 2-deoxyglucose (2-DG, 50 mM). (**I**) Time-dependent analysis of basal OCR following treatment with ligands for 6–48 h. (**J**) Mitochondrial stress test conducted on cardiac fibroblasts treated with ligands for 48 h. (**K**) Parameters describing the nature of oxygen consumption after ligand treatment for 48 h. (**L**) Visual representation of relationships between glycolysis, mitochondrial respiration, and myofibroblast formation. Schematic created in BioRender. Wells, C. (2026) https://BioRender.com/i7e9flu. *n* = 3–4 male mice per group. Statistics: Unpaired Student's t-test or Two-Way ANOVA with repeated measures, where appropriate. Data are shown as means ± SD.

Next, we investigated whether metabolic changes precede or coincide with establishment of the myofibroblast phenotype. As shown in Fig. 1D, glycolytic activity, as assessed by the extracellular acidification rate (ECAR), increased within 1.5 h of TGFβ treatment. Conversely, mitochondrial respiration, assessed via the oxygen consumption rate (OCR), decreased shortly after TGFβ provision (Fig. 1E). The increased reliance of fibroblasts on glycolysis early after ligand exposure is further evidenced by lower OCR/ECAR ratio in TGFβ-treated fibroblasts (Fig. 1F).

To further resolve the time course of glycolytic and respiratory activation, we examined metabolic changes occurring 6–48 h after ligand treatment. As shown in Fig. 1G, ECAR values were significantly elevated 6 and 48 h after TGFβ provision. Although not significantly elevated at 12 and 24 h after ligand treatment, mean ECAR rates remained higher than corresponding controls. A modified glycolysis stress test incorporating sequential injections of glucose, insulin, and 2-deoxyglucose (2-DG) demonstrated that myofibroblasts not only show elevated glycolytic rates upon glucose provision, but also demonstrate enhanced insulin-stimulated glycolysis (Fig. 1H; Suppl. Fig. 2). Notably, ECAR rates remained relatively similar throughout the 5–20 mM range of glucose, indicating that hyperglycemic conditions do not markedly elevate ECAR rates (Suppl. Fig. 2).

Unlike ECAR values, OCR values were elevated only when the myofibroblast phenotype was manifest, i.e., after 48 h of TGFβ treatment (Fig. 1I). Mitochondrial stress tests conducted after 48 h of TGFβ provision demonstrated significantly higher basal respiration and mitochondrial reserve and maximal capacities (Fig. 1J and K). Results of permeabilized fibroblast mitochondrial assays showed that myofibroblasts have higher state 3 and state 4_o_ succinate-supported respiration and higher state 3 palmitoylcarnitine-supported respiration, with pyruvate-supported respiration following similar trends, supporting the idea that myofibroblasts have generally higher mitochondrial respiratory capacity for a range of substrates (Suppl. Fig. 3). Collectively, these findings establish a temporal framework in which glycolysis is rapidly induced as an early metabolic response to TGFβ, preceding both respiratory activation and myofibroblast differentiation (Fig. 1L).

*TGFβ induces mitochondrial remodeling without increasing respiratory complex abundance*: Given that TGFβ signaling has been linked with altered mitochondrial dynamics [25], [26], [27], we assessed whether mitochondrial morphology was altered in TGFβ-treated cardiac fibroblasts. Interestingly, myofibroblast mitochondria were small and round compared with bFGF controls, as indicated by confocal microscopy analysis of MitoTracker Red-loaded fibroblasts (Fig. 2A–2C). Immunoblotting showed little change in representative subunits of the mitochondrial respiratory complexes, indicating that the increased respiratory capacity was not accompanied by a broad increase in the measured oxidative phosphorylation proteins (Fig. 2D–2F). Together, these findings indicate that enhanced respiratory capacity is accompanied by remodeling toward smaller, less elongated mitochondria without a detectable increase in representative respiratory complex subunits.

**Fig. 2:**
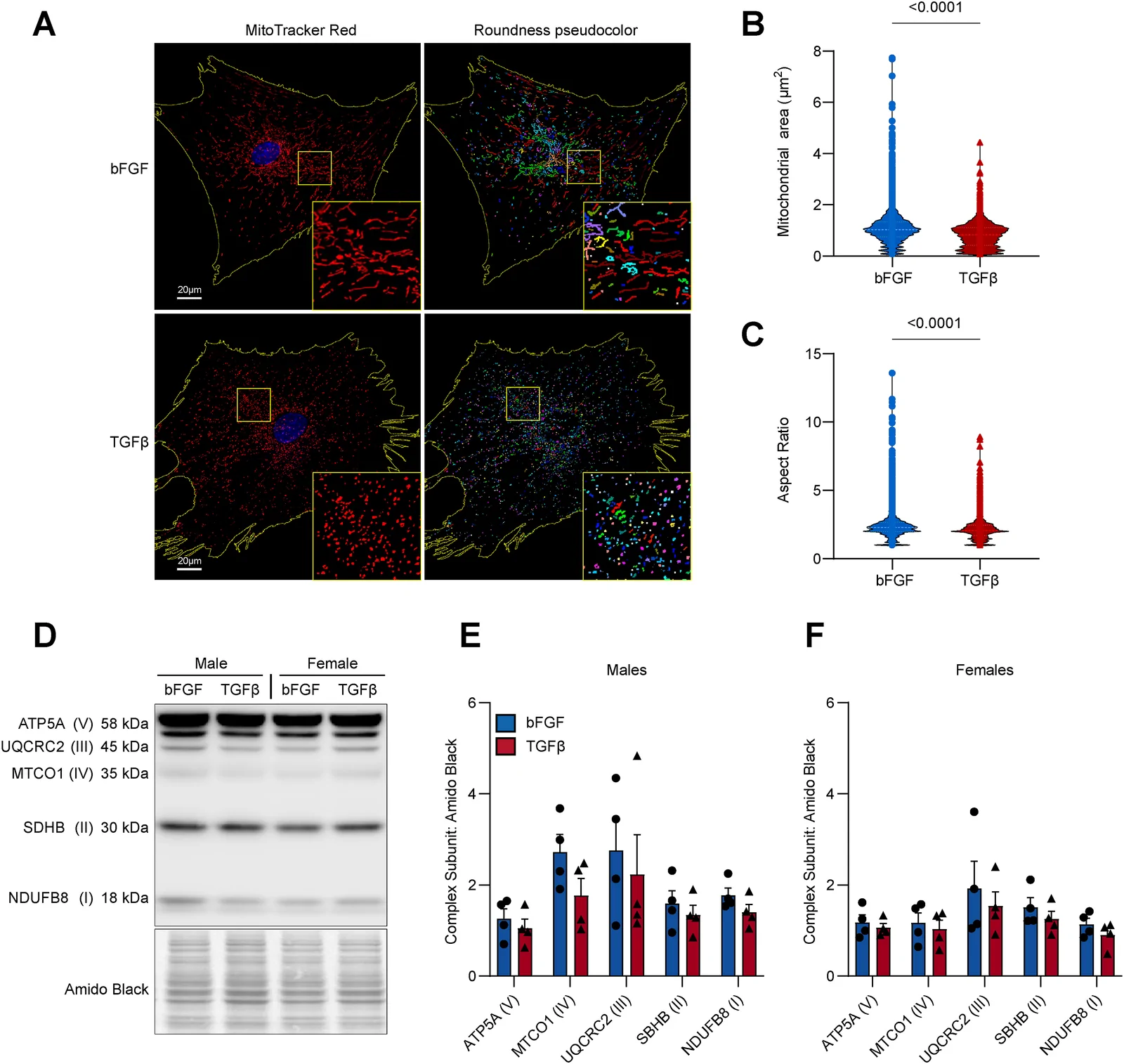
**TGFβ induces remodeling toward smaller, less elongated mitochondria without increasing representative respiratory complex subunits.** (**A**) Representative MitoTracker Red fluorescence images and corresponding roundness-pseudocolor maps of mitochondria segmented by adaptive thresholding following 2 d of TGFβ (20 ng/ml) or bFGF (20 ng/ml) treatment. (**B**) Violin plot showing the mitochondria area for TGFβ- and bFGF-treated cells. p < 0.0001 vs. bFGF (control) group. (**C**) Violin plot showing the aspect ratio for TGFβ- and bFGF-treated cells (n = 37–39 cells/analysis from 3 independent biological replicates). p < 0.0001 vs. bFGF (control) group. (**D**) Assessment of respiratory core proteins in whole cell lysates of bFGF and TGFβ-treated cells. (**E, F**) Quantification of panel D. *n* = 4 male and female mice per group. Statistics: Unpaired Student's t-test, comparing bFGF vs. TGFβ. Data in panels E and F are shown as means ± SD.

*Temporal resolution of TGFβ-induced changes in gene transcription*: To define when transcriptional remodeling emerges after TGFβ stimulation, we performed bulk RNA-seq on cardiac fibroblasts treated with TGFβ or bFGF for 1, 6, 12, 24, or 48 h. Principal component analysis revealed little separation between ligand groups at 1 h, whereas clear divergence was evident by 6 h (Fig. 3A). Only 394 genes were differentially expressed at 1 h, but 4188 differentially expressed genes (DEGs) were detected by 6 h, with the total DEG burden peaking at 24 h (Fig. 3B). These data indicate that TGFβ rapidly initiates a transcriptional program that expands substantially after 1 h of ligand treatment. Additionally, both upregulated and downregulated genes contributed at all time points, indicating broad bidirectional remodeling of the fibroblast transcriptome.

**Fig. 3:**
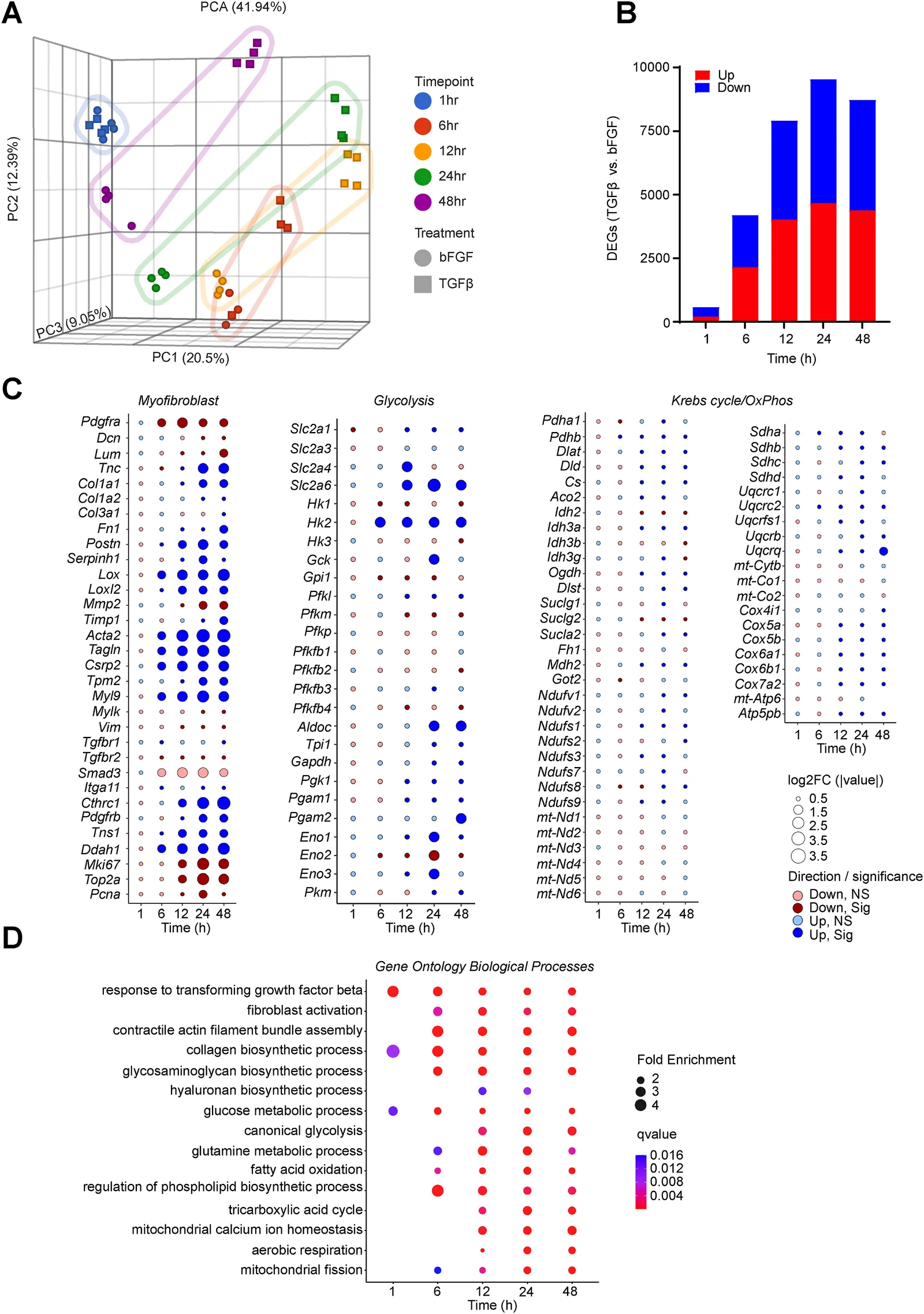
**Time-resolved RNA-seq reveals a phased transcriptional response to TGFβ stimulation.** Naïve adult mouse cardiac fibroblasts were treated with TGFβ (20 ng/ml) or bFGF control (20 ng/mL) for 1, 6, 12, 24, or 48 h before bulk RNA sequencing. (**A**) Principal component analysis of transcriptomic profiles. (**B**) Quantification of differentially expressed genes (DEGs) at each time point in TGFβ-treated fibroblasts relative to bFGF-treated controls. (**C**) Bubble plots of selected DEGs grouped by biological function, including myofibroblast differentiation/extracellular matrix remodeling (left panel), glycolysis and glucose metabolism (second panel), and Krebs cycle/oxidative phosphorylation (right panels). Differential expression was assessed using DESeq2 with Benjamini-Hochberg adjustment. OXPHOS, oxidative phosphorylation. n = 4 male biological replicates per group. (**D**) Gene Ontology Biological Process enrichment analysis of DEGs comparing TGFβ versus bFGF across treatment time points. The bubble plot highlights enriched terms corresponding to functional DEG categories, including myofibroblast differentiation, extracellular matrix remodeling, glycolysis and glucose metabolism, Krebs cycle activity, oxidative phosphorylation, and mitochondrial dynamics. The x axis shows treatment time points, and the y axis lists enriched biological processes. Bubble size indicates fold enrichment, and bubble color represents the adjusted q value from low to high using a red to blue gradient.

To identify the transcriptional programs underlying this response, DEGs were examined across curated functional categories related to myofibroblast differentiation, glycolysis, and Krebs cycle/oxidative phosphorylation (Fig. 3C). At 1 h, relatively few genes in these categories were altered. By 6 h, TGFβ induced a clear myofibroblast-associated signature, including reduced *Pdgfra*, increased *Pdgfrb*, altered TGFβ receptor expression, and upregulation of contractile- and differentiation-associated genes such as *Acta2*, *Postn*, *Tagln*, *Csrp2*, *Myl9*, and *Tns1*. Matrix remodeling genes, including *Lox* and *Loxl2*, were also induced. By 12 h, these changes were amplified, while proliferation associated genes such as *Mki67*, *Top2a*, and *Pcna* were reduced, consistent with progression toward a more differentiated and contractile fibroblast state.

TGFβ also remodeled metabolic gene programs over time. Glycolytic changes included sustained induction of *Hk2* with reciprocal reduction of *Hk1*, later upregulation of glucose transporter genes including *Slc2a1*, *Slc2a4*, and *Slc2a6*, and broader regulation of glycolytic enzymes from 12 h onward, including changes among enolase isoforms. In contrast, Krebs cycle and oxidative phosphorylation genes were altered mainly after 12 h and generally showed more modest transcriptional changes, with *Uqcrq* standing out as a more strongly induced mitochondrial complex III-associated gene. These findings suggest that early glycolytic activation after TGFβ exposure is likely caused by acute signaling and is subsequently reinforced by transcriptional remodeling of metabolic pathways.

To identify enriched biological pathways associated with each stage of the TGFβ response, DEGs were analyzed using clusterProfiler for Gene Ontology Biological Process enrichment, with visualization generated for selected terms related to myofibroblast activation, extracellular matrix remodeling, and metabolic reprogramming (Fig. 3D). Response to TGFβ, glucose metabolic process, and collagen biosynthetic process were enriched as early as 1 h, indicating early engagement of TGFβ signaling, metabolic, and matrix-associated programs. By 6 h, enrichment expanded to fibroblast activation, contractile actin filament bundle assembly, glycosaminoglycan biosynthesis, canonical glycolysis, glutamine metabolism, fatty acid oxidation, and mitochondrial fission. From 12 h onward, additional enrichment was observed for hyaluronan biosynthesis, phospholipid biosynthetic regulation, tricarboxylic acid cycle, mitochondrial calcium ion homeostasis, aerobic respiration, and mitochondrial fission, suggesting later transcriptional activation of biosynthetic and oxidative pathways as well as mitochondrial dynamics. These temporal enrichment patterns support a coordinated, stepwise transition toward metabolic reprogramming, myofibroblast activation, and extracellular matrix remodeling.

To complement this analysis, Gene Ontology Biological Process enrichment was performed at each time point, with the 15 most significantly enriched terms ranked by fold enrichment (Suppl. Fig. 4). At 1 h, the top enriched terms included responses to glucose starvation, fatty acids, IL-1, mechanical stress, and protein misfolding, together with several immune and developmental or morphogenetic processes. By 6 h, enriched terms expanded to include cell migration, epithelial-to-mesenchymal transition-related developmental programs, oxidative stress responses, GPCR and GTP-binding signaling, stress-granule assembly, and mitochondrion-endoplasmic reticulum membrane tethering. At 12 h, cell cycle and protein-processing programs became more prominent, including mitotic DNA replication, cytokinesis, DNA-strand elongation, centrosome organization, spindle-checkpoint regulation, signal peptide processing, and regulation of translation during endoplasmic reticulum stress. At 24 h, genes associated with mitotic and DNA replication processes were prominently enriched among the differentially expressed genes, largely reflecting remodeling of cell-cycle programs. At 48 h, DNA replication, chromosome condensation, and spindle organization remained among the top enriched processes, accompanied by adherens junction assembly, macropinocytosis, elastic-fiber assembly, and several differentiation- and development-associated categories. Considered together with the focused differential expression and pathway analyses, these findings support a phased, time-dependent remodeling of the cardiac fibroblast transcriptome in response to TGFβ relative to bFGF in which an initially limited response occurs at 1 h, expands substantially by 6 h, and subsequently encompasses cell cycle regulation, stress responses, developmental- and differentiation-associated programs, cell adhesion, and extracellular matrix remodeling.

*Stable isotope-resolved metabolomics reveals temporal changes in glucose carbon incorporation into catabolic and biosynthetic metabolite pools*: To further understand the nature of the metabolic changes associated with myofibroblast differentiation, we supplanted glucose in the fibroblast culture medium with ^13^C_6_-glucose for 6 h, enabling stable isotope-assisted resolution of glucose-utilizing pathways [28]. In previous murine cardiac fibroblast studies, we found that selected metabolite pools approached an isotopic plateau by 6 h, with dynamic phase labeling occurring in pentose phosphate pathway and nucleotide biosynthetic pathway metabolites. Krebs cycle intermediates such as citrate show near steady-state labeling by 6 h of ^13^C_6_-glucose provision [29]. Therefore, we performed reverse time course studies, in which TGFβ (or bFGF) was provided for 6–48 h, with media containing ^13^C_6_-glucose provided only during the last 6 h of incubation (Fig. 4A) and examined effects on the abundance and ^13^C fractional enrichment of metabolites in glycolysis, ancillary pathways of glucose metabolism, and Krebs cycle intermediates.

**Fig. 4:**
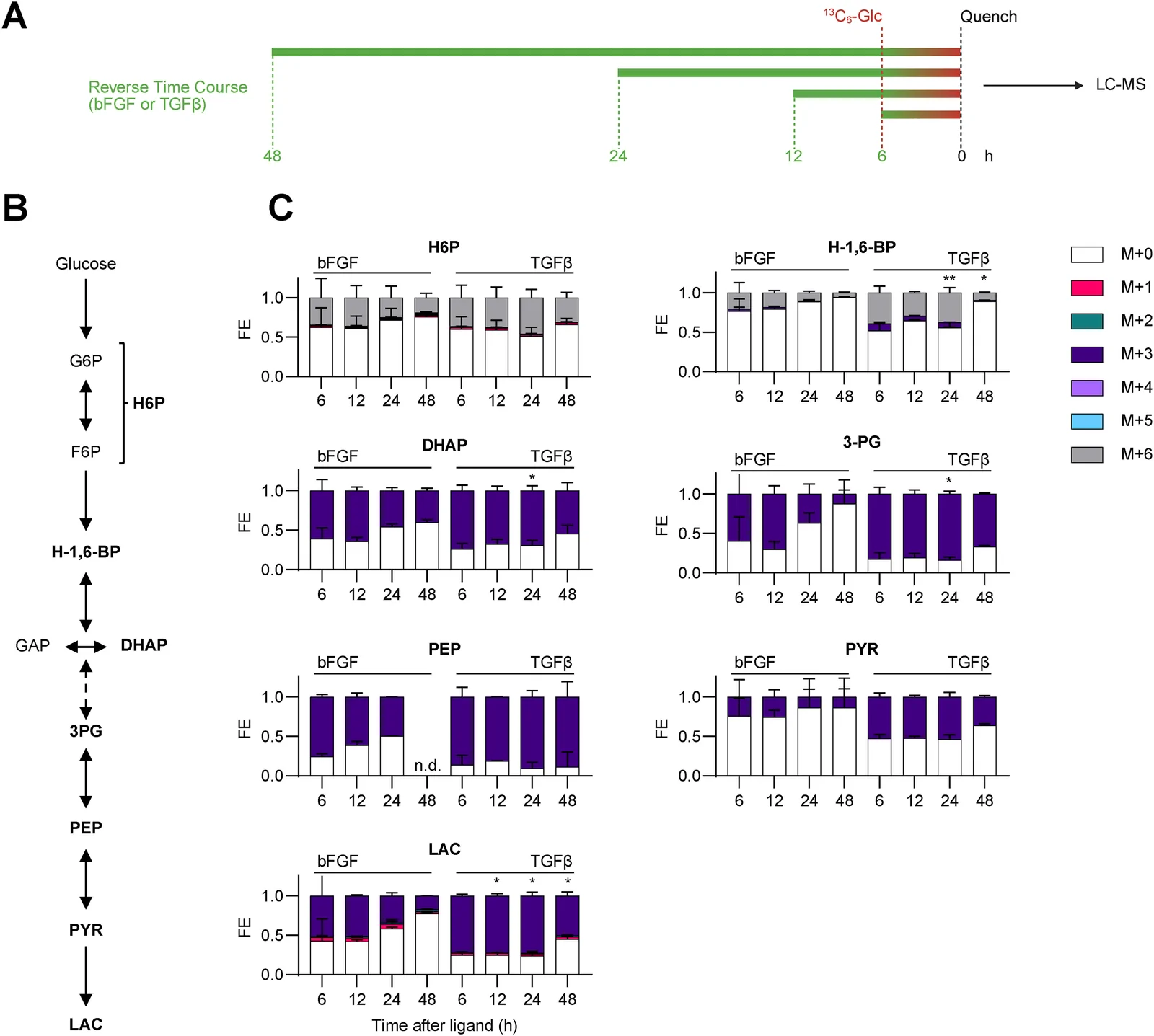
**TGFβ increases glucose-derived labeling of glycolytic metabolites.** Cardiac fibroblasts isolated from adult male mice were treated with TGFβ or bFGF for 6–48 h, and unlabeled glucose was supplanted with ^13^C_6_-glucose for the last 6 h of the protocol. Metabolites were then extracted for stable isotope-resolved metabolomics. (**A**) Schematic of time course. (**B**) Schematic of glycolysis. Bolded metabolites were included in the analysis. (**C**) Stacked bar fractional enrichment (FE) isotopologue data of glycolytic intermediates. Total ^13^C enrichment values were transformed via logit transformation prior to statistical analysis. *n* = 3 male mice per group. For statistical analyses, two-way ANOVA repeated measures with Sidak's post-hoc test was performed. Asterisks indicate significance of total fractional enrichment (TFE) after logit transformation (TFE and normalized protein abundance data shown in Suppl. Fig. 5). Data are shown as means ± SD. Values not detected for certain time points are represented as “n.d.”. H6P, hexose-6-phosphate; H-1,6-BP, hexose-1,6-bisphosphate; DHAP, dihydroxyacetone phosphate; 3-PG, 3-phosphoglycerate; PEP, phosphoenolpyruvate; PYR, pyruvate; LAC, lactate. *p < 0.05; **p < 0.001.

Glycolysis: Of the metabolites detected in glycolysis (Fig. 4B, bold), the overall abundances were not significantly different, with the exception of 3 PG, which was elevated after 24 h of TGFβ treatment (Suppl. Fig. 5). Analysis of total fractional enrichment (TFE) revealed that TGFβ treatment increased ^13^C enrichment in several glycolytic intermediates, including hexose 1,6-bisphosphate (H-1,6-BP), dihydroxyacetone phosphate (DHAP), and 3-phosphoglycerate (3 PG) after 24 h of ligand provision, with significant increases in lactate (LAC) enrichment occurring at 12 h and afterwards (Suppl. Fig. 5). Fractional enrichment and isotopologue analysis showed expected ^13^C labeling patterns, with generally higher fractional enrichment in the m+6 isotopologues of 6-carbon intermediates and in the m+3 isotopologues of 3-carbon intermediates, especially after 24 h of TGFβ treatment (Fig. 4C). The presence of m+3 H-1,6-BP is consistent with recombination of labeled and unlabeled triose-phosphate pools through reversible glycolytic reactions. The appearance of m+1 lactate together with m+1 malate and aspartate is compatible with secondary recycling of glucose-derived carbon, potentially involving ^13^CO_2_ fixation and pyruvate-cycling pathways, although the specific route cannot be resolved from these data. Together with the extracellular flux measurements, these data support increased utilization of extracellular glucose by TGFβ-treated cardiac fibroblasts.

Ancillary biosynthetic pathways of glucose metabolism: Because glycolytic intermediates can enter into ancillary pathways to regulate redox state and the biosynthesis of cellular building blocks, we next examined glucose carbon incorporation and the relative abundances of intermediates and end products of the pentose phosphate, glucuronate, hexosamine biosynthetic, glycerolipid, and serine biosynthesis pathways. As shown in Suppl. Fig. 6 relative abundances were not different in any of the measured biosynthetic pathway intermediates or end products; however, UDP-*N*-acetyl hexosamine (UDP-HexNAc), the end product of the hexosamine biosynthetic pathway; UDP-hexose (UDP-Hex), a nucleotide-sugar pool involved in glycogen and glycoconjugate synthesis; and glycerol 3-phosphate (G3P) showed elevated ^13^C enrichment as early as 12 h after TGFβ provision. UDP-hexuronic acid (UDP-HexA), an essential building block for hyaluronic acid, and adenosine monophosphate (AMP) demonstrated elevated ^13^C enrichment 48 h after TGFβ treatment. As shown in Fig. 5, fractional enrichment isotopologue analysis indicated expected labeling patterns for ancillary biosynthetic pathway intermediates and end products. Ribose 5-phosphate was detected predominantly as the m+0 and m+5 isotopologues, consistent with direct production from ^13^C_6_-glucose via the oxidative pentose phosphate pathway. The presence of m+1, m+2, and m+3 isotopologues, despite hexose 6-phosphate being limited to m+0, m+3, and m+6 (see Fig. 4), could indicate carbon rearrangement through the reversible non-oxidative pentose phosphate pathway as well as oxidation of positional m+3 hexose isotopomers. AMP showed the expected m+5 labeling pattern, indicating labeled ribose incorporation, with TGFβ-treated fibroblasts showing higher labeling throughout the time course. UDP-Hex and UDP-HexA were composed primarily of m+6 isotopologues, with relatively minor higher-mass labeling (e.g., m+11), whereas UDP-HexNAc displayed a broader isotopologue distribution with substantial higher-order ^13^C enrichment. This more extensive labeling of UDP-HexNAc appeared most evident in the TGFβ condition at 12–24 h, suggesting greater routing of glucose-derived carbon into the hexosamine nucleotide sugar pool. Alanine and serine were both partially ^13^C-labeled, although alanine displayed a broader isotopologue distribution and serine remained predominantly m+0. The m+3 species of G3P was elevated at 12 and 24 h post-TGFβ treatment, whereas glycerophosphoglycerol was composed largely of unlabeled carbon with minor m+3, m+4, and m+6 enrichment. Collectively, these data indicate that TGFβ stimulation increases glucose-derived labeling of nucleotide sugar, glycerol 3-phosphate, glycerophosphoglycerol, and AMP pools.

**Fig. 5:**
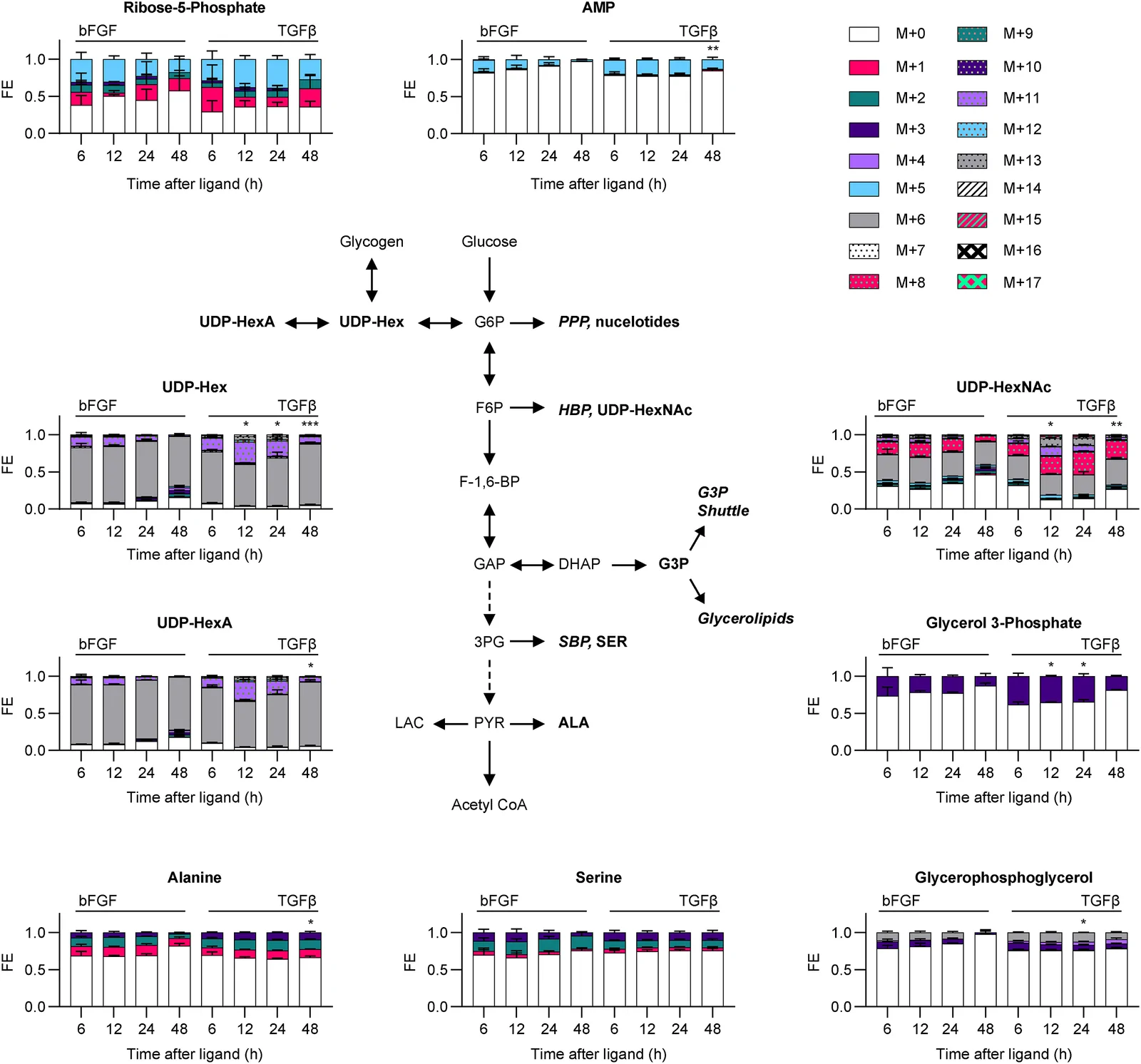
**TGFβ increases glucose-derived labeling of biosynthetic metabolite pools.** Cardiac fibroblasts isolated from adult male mice were treated with TGFβ or bFGF for 6–48 h, and unlabeled glucose was supplanted with ^13^C_6_-glucose for the last 6 h of the protocol; metabolites were then extracted for stable isotope-resolved metabolomics. Included in the center is a schematic of ancillary biosynthetic pathways. Bolded metabolites were included in the analysis. Fractional enrichment (FE) isotopologue data of ancillary biosynthetic pathway intermediates. Total fractional enrichment (TFE) values were transformed via logit transformation prior to statistical analysis. *n* = 3 male mice per group. For statistical analyses, two-way ANOVA repeated measures with Sidak's post-hoc test was performed. Asterisks indicate significance of total fractional enrichment (TFE) after logit transformation (TFE and protein-normalized abundance data are shown in Suppl. Fig. 6). Data are shown as means ± SD. ALA, alanine; AMP, adenosine monophosphate; G3P, glycerol 3-phosphate; R5P, ribose 5-phosphate; UDP-Hex, uridine diphosphate-hexose; UDP-HexA, UDP-hexuronate; UDP-HexNAc, UDP-*N*-acetylhexosamine. *p < 0.05; **p < 0.001; ***p < 0.0001.

Krebs cycle: To assess how glucose carbon contribution to the Krebs cycle changes during myofibroblast differentiation, we examined the abundance and ^13^C labeling of tricarboxylic acid intermediates following TGFβ stimulation using the reverse time-course labeling strategy mentioned above (see Fig. 4A). During the first 24 h of ligand treatment, the total pool sizes of most Krebs cycle intermediates were unchanged, although isocitrate and α-ketoglutarate abundance increased after 12 h of TGFβ exposure. By 48 h, however, total fractional ^13^C enrichment increased in several Krebs cycle intermediates (Suppl. Fig. 7), coincident with the emergence of the myofibroblast phenotype and increased mitochondrial respiratory capacity (see Fig. 1). Because previous time-course studies in murine cardiac fibroblasts showed that citrate labeling approaches an isotopic plateau within 6 h of ^13^C_6_-glucose provision [29], these measurements provide an estimate of the relative contribution of glucose-derived carbon to the citrate pool, although they do not quantify absolute pathway flux. At 48 h, more than half of citrate molecules in TGFβ-treated fibroblast contained at least one glucose-derived carbon. Labeling was lower in α-ketoglutarate and downstream intermediates, consistent with dilution by unlabeled substrates, including glutamine-derived carbon, as reported [17].

Early time points showed little evidence of increased glucose-derived carbon entry into the Krebs cycle intermediates, paralleling the absence of increased mitochondrial respiration before 48 h. Isotopologue analysis revealed increased citrate m+2, m+4, and m+5 labeling in TGFβ-treated fibroblasts (Fig. 6). Citrate m+2 is consistent with pyruvate dehydrogenase-dependent entry of glucose-derived acetyl CoA, whereas higher-order citrate isotopologues are compatible with condensation of labeled acetyl CoA with labeled oxaloacetate and continued cycling of glucose-derived carbon. Isocitrate showed comparably little change in labeling despite the increase in citrate enrichment, potentially reflecting compartmentation or differential turnover of citrate and isocitrate pools; whether export of citrate for biosynthesis (e.g., to fatty acids) contributes to this divergence cannot be determined from these measurements alone. Malate labeling increased at 24 h, including a modest increase in m+3 enrichment, with aspartate exhibiting a similar pattern. Because malate and aspartate m+3 can arise from pyruvate carboxylase-dependent formation of m+3 oxaloacetate, these findings are consistent with an anaplerotic contribution of glucose-derived pyruvate, although multiple cycle labeling and carbon recycling may also contribute. Taken as a whole, these data support a phasic metabolic response to TGFβ in which early glycolytic activation is followed by increased routing of glucose carbon into biosynthetic pathways and, later, enhanced entry and cycling of glucose-derived carbon through the Krebs cycle.

**Fig. 6:**
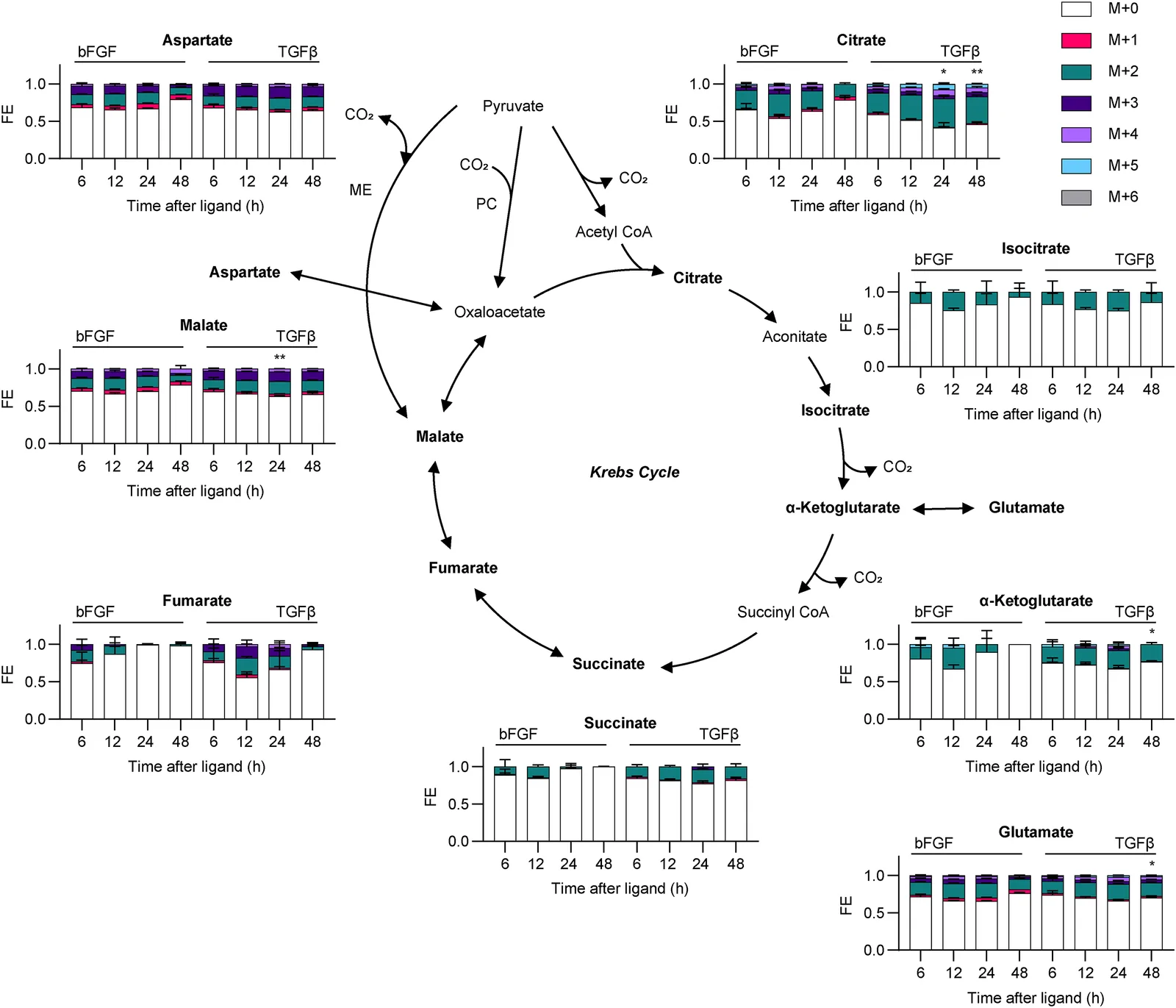
**TGFβ induces a delayed increase in glucose-derived labeling of Krebs cycle intermediates.** Primary cardiac fibroblasts isolated from adult male mice were treated with TGFβ or bFGF for 6–48 h, and unlabeled glucose was supplanted with ^13^C_6_-glucose for the last 6 h of the protocol. Metabolites were then extracted for stable isotope-resolved metabolomics. Included in the center is a schematic of the Krebs cycle. Bolded metabolites were included in the analysis. Fractional enrichment isotopologue data of Krebs cycle intermediates are shown in stacked bar plots. Total ^13^C enrichment values were transformed via logit transformation prior to statistical analysis. *n* = 3 male mice per group. For statistical analyses, two-way ANOVA repeated measures with Sidak's post-hoc test was performed. Asterisks indicate significance of total fractional enrichment (TFE) after logit transformation (TFE and protein-normalized abundance data are shown in Suppl. Fig. 7). Data shown as means ± SD. *p < 0.05; **p < 0.001.

*Glycolysis regulates myofibroblast differentiation*: To determine how glycolytic rate affects cardiac myofibroblast differentiation, we first examined the effect of glycolysis inhibition on fibroblast metabolism and TGFβ-mediated myofibroblast differentiation. Guided by the RNA-seq data, we found that cardiac fibroblasts express the core enzymes of the Leloir pathway [30], including GALK1, GALT, GALE, and GALM, indicating that they can convert galactose to glucose 1-phosphate and thereby sustain downstream glucose metabolism when galactose replaces glucose, albeit at a substantially lower glycolytic rate. Indeed, cardiac fibroblasts cultured with galactose showed a ∼3–4-fold decrease in glycolytic rate without affecting mitochondrial oxidative phosphorylation (Fig. 7A and B). Switching the carbohydrate substrate to galactose showed a modest effect on fibroblast viability, as indexed by LDH cytotoxicity assay (Suppl. Fig. 8). Next, we assessed how culture in galactose influences myofibroblast differentiation. Culture in galactose during 48 h of TGFβ stimulation completely abolished increases in collagen 1a1 (Col1a1) and diminished POSTN upregulation, without affecting upregulation of αSMA (Fig. 7C and D).

**Fig. 7:**
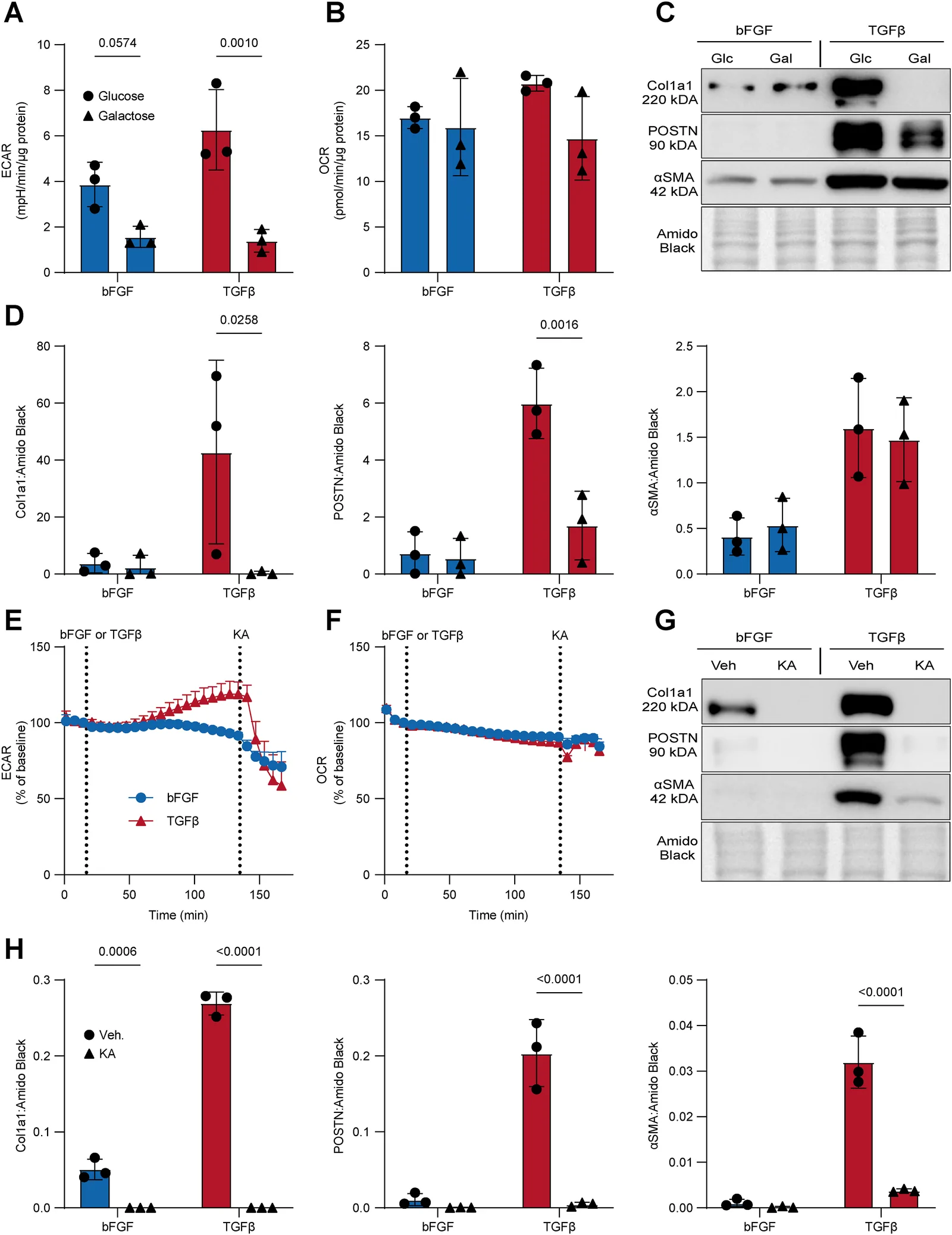
**Glycolytic restriction differentially impairs TGFβ-induced myofibroblast differentiation and collagen accumulation.** Primary cardiac fibroblasts isolated from adult male mice were treated with transforming growth factor-β (TGFβ; 20 ng/ml) or basic fibroblast growth factor (bFGF; 20 ng/mL). (**A, B**) Basal extracellular acidification rate (ECAR; A) and oxygen consumption rate (OCR; B) after 48 h of ligand treatment in medium containing glucose (Glc) or in medium in which glucose was replaced with galactose (Gal). (**C**) Representative immunoblots and (**D**) densitometric quantification of type I collagen α1 chain (COL1A1), periostin (POSTN), and α-smooth muscle actin (αSMA) after 48 h under the indicated carbohydrate and ligand conditions. (**E, F**) Real-time ECAR (E) and OCR (F), expressed as percentages of the pre-injection baseline, during sequential acute administration of bFGF or TGFβ followed by the glyceraldehyde-3-phosphate dehydrogenase inhibitor koningic acid (KA; 20 μM). (**G**) Representative immunoblots and (**H**) densitometric quantification of COL1A1, POSTN, and αSMA after 48 h of treatment with bFGF or TGFβ in the presence of vehicle (Veh) or KA (20 μM). ECAR and OCR values in panels A and B were normalized to total protein. Immunoblot densitometry was normalized to total lane protein visualized by amido black staining. Data are means ± SD from three independent fibroblast isolations, each obtained from a different male mouse. Statistical comparisons in A, B, D, and H were performed by two-way ANOVA followed by Šídák's multiple-comparisons test; exact p values are shown. Glc, glucose; Gal, galactose; Veh, vehicle; KA, koningic acid.

As an additional approach, we also inhibited the payoff phase of glycolysis by treating fibroblasts with koningic acid (KA), which inhibits glyceraldehyde 3-phosphate dehydrogenase (GAPDH) by covalently modifying its active site cysteine [31]. To confirm whether KA inhibits glycolysis in TGFβ-treated cells, we treated cardiac fibroblasts with bFGF or TGFβ, and, after induction of the early glycolytic response, exposed the cells to KA (20 μM) and examined changes in ECAR in real time. As shown in Fig. 7E, acute KA administration rapidly reduced ECAR, including the elevated ECAR observed in TGFβ-treated fibroblasts, without producing a comparable acute reduction in OCR (Fig. 7F). The presence of KA prevented upregulation of Col1a1, POSTN, and αSMA, indicating that blockade of the payoff phase of glycolysis abolishes TGFβ-induced myofibroblast formation (Fig. 7G and H). Notably, KA did not cause cell death as measured by LDH cytotoxicity assay (Suppl. Fig. 8). These findings indicate that glycolytic rate is a major regulator of myofibroblast differentiation.

*Heightened glycolytic activity is not sufficient for myofibroblast differentiation*: We next asked whether increasing glycolytic activity, in the absence of a profibrotic stimulus, is sufficient to promote myofibroblast differentiation. To address this question, we used two complementary adenoviral gain-of-function approaches: overexpression of glucose transporter 1 (GLUT1) to increase cellular glucose uptake and overexpression of a glycolysis-promoting mutant PFK2 construct (Glyco^Hi^ [32], [33]) to increase glycolytic activity at the level of the committed step in glycolysis, i.e., phosphofructokinase 1. These approaches complement the loss-of-function studies described above and permit a direct test of whether increasing glycolytic activity through distinct mechanisms is sufficient to initiate the myofibroblast program.

Glut1 adenoviral transduction increased GLUT1 abundance and glycolysis in bFGF-treated fibroblasts, confirming that enhanced glucose transport was sufficient to elevate glycolytic activity (Suppl. Fig. 9A and 9B). Despite increasing glycolytic activity in bFGF-treated fibroblasts, GLUT1 overexpression did not induce POSTN, αSMA, or collagen and produced only modest changes in these markers in TGFβ-treated cells (Suppl. Fig. 9C). To determine whether this lack of differentiation reflected the particular site at which glycolysis was enhanced, we next increased glycolytic activity using the Glyco^Hi^ approach. Glyco^Hi^ construct overexpression similarly increased glycolytic activity in bFGF-treated fibroblasts, but did not induce POSTN, αSMA, or other markers of myofibroblast differentiation (Suppl. Fig. 9D and 9E). Thus, increasing glycolysis through either enhanced glucose transport or downstream stimulation of glycolytic flux was insufficient to initiate the myofibroblast phenotype in the absence of a profibrotic stimulus. Taken together with the loss-of-function experiments, these findings indicate that sufficient glycolytic activity is required to support the differentiated myofibroblast phenotype but is not independently sufficient to initiate it. Rather, glycolytic activation appears to provide a permissive metabolic state that enables execution of the differentiation program initiated by TGFβ-dependent signaling.

*Inhibition of mitochondrial electron transport and ATP synthesis preferentially suppresses collagen abundance while preserving myofibroblast marker induction*: To determine if mitochondrial activity influences myofibroblast formation, we co-treated cardiac fibroblasts with bFGF or TGFβ and the Complex I inhibitor rotenone (1 μM) or vehicle (DMSO) for 48 h and assessed Col1a1 and myofibroblast marker levels. As shown in Fig. 8A and B, rotenone completely abolished Col1a1 expression and modestly decreased POSTN and αSMA abundance. Similarly, blockade of mitochondrial ATP synthase with oligomycin markedly diminished Col1a1 levels and modestly diminished POSTN, but did not affect αSMA (Fig. 8C and D). Neither rotenone nor oligomycin promoted significant cytotoxicity compared with DMSO control (Suppl. Fig. 8). These findings suggest that mitochondrial activity plays a prominent role in collagen upregulation, but has only marginal effects on markers of myofibroblast differentiation.

**Fig. 8:**
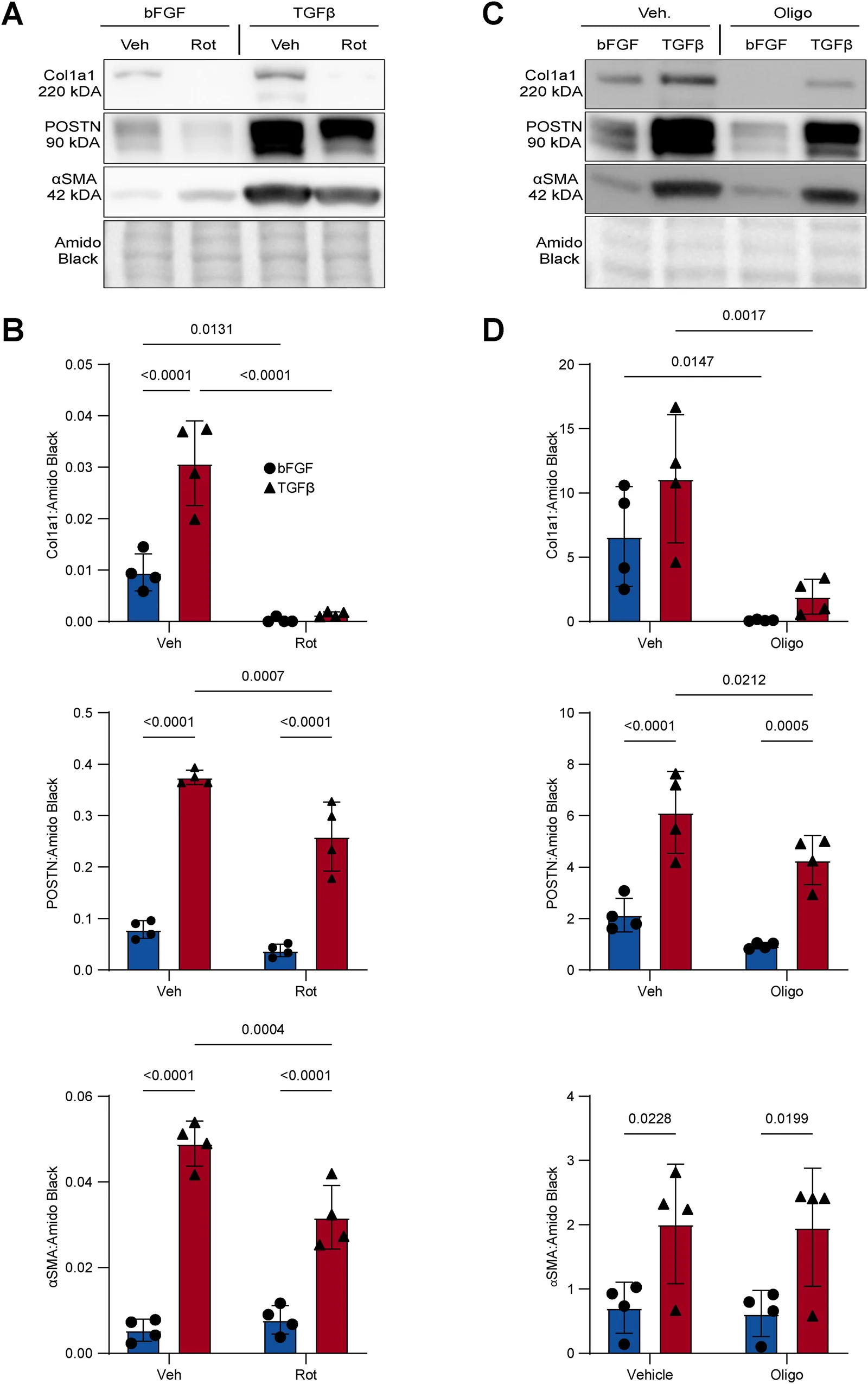
**Inhibition of mitochondrial respiration preferentially suppresses collagen accumulation while largely preserving myofibroblast marker induction.** Primary cardiac fibroblasts isolated from adult male mice were treated for 48 h with basic fibroblast growth factor (bFGF; 20 ng/mL) or transforming growth factor-β (TGFβ; 20 ng/ml) in the presence of vehicle or inhibitors of mitochondrial respiration. (**A**) Representative immunoblots and (**B**) densitometric quantification of type I collagen α1 chain (COL1A1), periostin (POSTN), and α-smooth muscle actin (αSMA) following treatment with vehicle (Veh) or the Complex I inhibitor rotenone (Rot; 1 μM). (**C**) Representative immunoblots and (**D**) densitometric quantification of COL1A1, POSTN, and αSMA following treatment with vehicle or the ATP synthase inhibitor oligomycin (Oligo; 1 μM). Immunoblot densitometry was normalized to total lane protein visualized by amido black staining. Data are means ± SD from four independent fibroblast isolations, each obtained from a different male mouse. Statistical comparisons were performed by two-way ANOVA followed by Šídák's multiple-comparisons test; exact p values are shown. Veh, vehicle; Rot, rotenone; Oligo, oligomycin.

*Effects of glycolysis and mitochondrial metabolism on myofibroblast contractile activity*: In addition to secreting extracellular matrix, a major function of myofibroblasts is to promote wound contraction and closure [34], [35]. Therefore, to determine the effect of metabolism on the contractile activity of fibroblasts, we performed a series of gel contraction assays, in which cardiac fibroblasts were seeded into a collagen gel matrix and then treated with ligands in the absence or presence of inhibitors of glycolysis or mitochondrial activity. As shown in Fig. 9, both KA and rotenone diminished TGFβ-induced contractile activity, which could suggest that loss of glycolysis- or mitochondria-derived ATP production is critical for myofibroblast contraction. To test the effect of mitochondria-derived ATP production more directly, this experiment was repeated using oligomycin. Interestingly, oligomycin promoted fibroblast gel contraction even in the bFGF-treated condition, suggesting that loss of mitochondrial ATP synthesis alone is insufficient to suppress contractile activity. In light of prior work showing that ATP synthase inhibition triggers rapid peri-mitochondrial actin polymerization [36], these findings raise the possibility that oligomycin triggers actin polymerization that promotes fibroblast contraction. Because actin cytoskeletal organization and stress-fiber formation contribute importantly to fibroblast force generation [37], this response could reinforce gel contraction despite inhibition of mitochondrial ATP synthesis, which may be important to facilitate wound contraction even in tissue areas where oxygen is limiting.

**Fig. 9:**
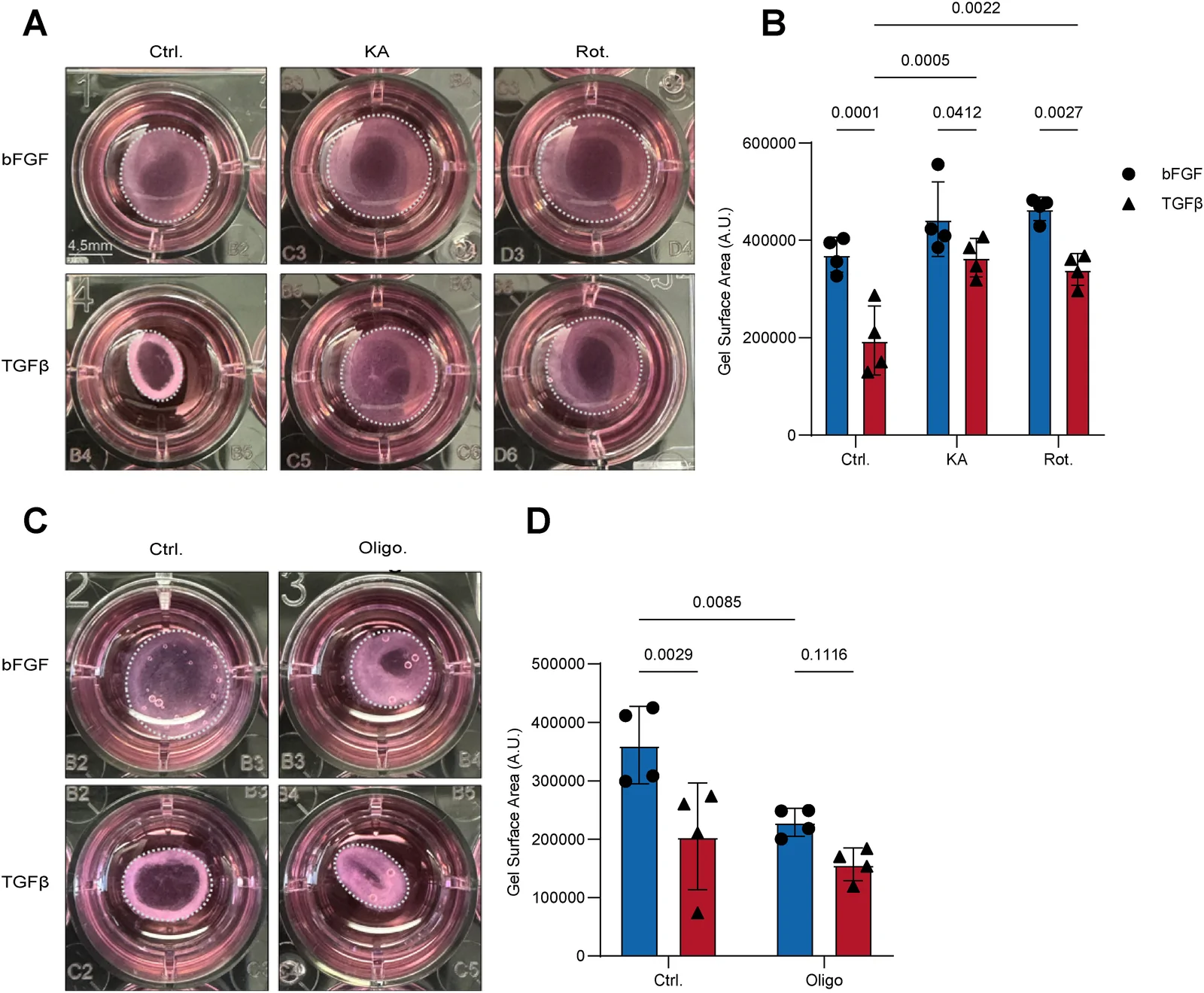
**Glycolytic and mitochondrial inhibitors differentially regulate fibroblast-mediated collagen gel contraction.** Primary cardiac fibroblasts isolated from adult male mice were embedded in collagen gels and treated with basic fibroblast growth factor (bFGF; 20 ng/mL) or transforming growth factor-β (TGFβ; 20 ng/ml) in the presence of the indicated metabolic inhibitors. (**A**) Representative images of collagen gels after 5 d of treatment with control vehicle (Ctrl), the glyceraldehyde-3-phosphate dehydrogenase inhibitor koningic acid (KA; 20 μM), or the mitochondrial Complex I inhibitor rotenone (Rot; 1 μM). (**B**) Quantification of residual gel surface area for the conditions shown in A. (**C**) Representative images of collagen gels after 5 d of treatment with control vehicle or the ATP synthase inhibitor oligomycin (Oligo; 1 μM). (**D**) Quantification of residual gel surface area for the conditions shown in C. Smaller residual gel area indicates greater fibroblast-mediated gel contraction and matrix compaction. Data are means ± SD from four independent fibroblast isolations, each obtained from a different male mouse. Statistical comparisons were performed by two-way analysis of variance followed by Šídák's multiple-comparisons test; exact P values are shown. Ctrl, control; KA, koningic acid; Rot, rotenone; Oligo, oligomycin.

## 3 Discussion

Myofibroblast differentiation is generally viewed as a transcriptionally driven response to profibrotic signaling, with metabolic remodeling serving primarily to meet the energetic and biosynthetic demands of the activated state. The findings of this study support a modified view in which metabolic remodeling is not merely a downstream consequence of differentiation, but an early and functionally important component of the fibroblast response to TGFβ. By integrating extracellular flux analysis, time-resolved transcriptomics, stable isotope-resolved metabolomics, and metabolic perturbation studies, we show that TGFβ rapidly increases glycolysis, while transcriptional remodeling develops progressively and glucose-derived carbon is increasingly redistributed into biosynthetic and mitochondrial metabolite pools, accompanied later by greater respiratory capacity. Metabolic perturbation further revealed that restricting glycolytic activity impairs acquisition of the complete myofibroblast phenotype, whereas increasing glycolytic activity through either GLUT1 or Glyco^Hi^ overexpression was insufficient to initiate differentiation in the absence of a profibrotic stimulus. In contrast, manipulation of mitochondrial metabolism exerted more selective effects on collagen abundance and contractile activity. Together, these findings support a multidimensional model of myofibroblast differentiation in which TGFβ-dependent signaling provides instructive cues, early glycolytic activation acts permissively to enable execution of the differentiation program, and later transcriptional and mitochondrial remodeling reinforce selected effector functions. Rather than producing a single metabolically activated state, these processes establish partially separable metabolic dependencies for matrix production, canonical differentiation markers, and contractile behavior.

The finding that heightened glycolysis is an early regulator of myofibroblast differentiation is a central observation of this study. Previous studies established that TGFβ induces glycolytic reprogramming in fibroblasts, with increased ECAR evident by 6 h in lung fibroblasts and accompanied by subsequent induction of PFKFB3, which peaked at approximately 8 h [10]. A similar temporal pattern has been observed in hepatic stellate cells, in which glycolysis increases by 6 h and GLUT1 abundance begins to rise within several hours of TGFβ stimulation [38]. In the present study, however, glycolytic rate increased within approximately 1–1.5 h of TGFβ exposure, well before robust induction of POSTN and αSMA and before the extensive transcriptomic separation observed at 6 h and later. Thus, glycolytic activation appears to occur considerably earlier than has generally been resolved in prior fibroblast studies. This temporal relationship does not imply that metabolism precedes all transcriptional responses, because several hundred genes were already differentially expressed at 1 h and early enriched processes included TGFβ-responsive, metabolic, and matrix-associated programs. The rapidity of the metabolic response nevertheless suggests that its initiation may rely substantially on acute signaling to pre-existing metabolic machinery, with subsequent transcriptional induction of glucose transporters and glycolytic enzymes reinforcing the glycolytic state.

The signaling mechanisms that establish these temporally distinct metabolic phases remain to be determined. TGFβ activates canonical SMAD2/3 signaling as well as noncanonical pathways including PI3K-AKT-mTOR, ERK, p38, and JNK, which may contribute differently over the course of fibroblast activation [39]. The rapid increase in glycolysis observed within approximately 1–1.5 h, when relatively little metabolic transcriptional remodeling was evident, suggests that the initial response may involve acute regulation of pre-existing metabolic machinery rather than requiring extensive new gene expression. Consistent with this possibility, TGFβ has been shown to regulate GLUT1 expression and glycolysis through SMAD, p38 MAPK, and PI3K-AKT signaling in activated mesenchymal cells [38]. At later times, transcriptional responses could reinforce and diversify the metabolic program. In fibroblasts, TGFβ-dependent mTORC1 signaling promotes glycolytic and mitochondrial activity and, in cooperation with SMAD3, activates ATF4-dependent glucose uptake and serine-glycine biosynthesis required to support collagen production [13], [40]. ERK, p38, JNK, and AKT may provide additional context-dependent inputs, although their specific contributions to the metabolic phases identified here remain unresolved. Thus, the early glycolytic and later oxidative/anabolic phases may reflect sequential or overlapping outputs of multiple TGFβ signaling branches rather than activation of a single pathway. Defining which signaling nodes control each phase will require direct temporal perturbation studies.

The stable isotope studies indicate that increased glucose use extends beyond glycolysis and lactate production. The reverse time-course SIRM strategy used here was designed to resolve how glucose utilization changes as fibroblasts progress through differentiation. Rather than varying tracer exposure time, fibroblasts were exposed to TGFβ for progressively longer periods but received [U-^13^C_6_]-glucose only during the final 6 h of each treatment interval. Thus, isotope incorporation was compared over an equivalent labeling window at different stages of the TGFβ response, permitting temporal changes in the relative contribution of glucose carbon to be distinguished from differences caused simply by tracer duration. This approach was informed by prior studies in cardiac fibroblasts showing that a 6-h labeling interval provides steady-state or near steady-state labeling for several glucose-derived metabolite pools, including glycolytic intermediates, citrate, UDP-Hexuronic acid, and UDP-HexNAc, while retaining dynamic information in pentose phosphate and nucleotide biosynthetic pathways [29]. TGFβ increased glucose-derived labeling of glycolytic intermediates and, subsequently, of UDP-Hex, UDP-HexNAc, UDP-Hexuronic acid, AMP, glycerol 3-phosphate, and glycerophosphoglycerol, consistent with progressive redistribution of glucose carbon into nucleotide sugar, nucleotide, and glycerolipid pathways. Fructose 6-phosphate and glucose-derived acetyl units contribute to UDP-N-acetylhexosamine production, whereas glucose 6-phosphate supports formation of UDP-glucuronate, linking glycolysis to protein glycosylation, proteoglycan synthesis, and hyaluronan production [11]. Cardiac fibroblasts exhibit substantial hexosamine and UDP-glucuronate pathway activity [29] and are an important source of HAS2-dependent high-molecular-weight hyaluronan after myocardial injury [4]. Glucose-derived 3-phosphoglycerate can, in some conditions, also support serine and glycine synthesis for collagen production [13], [15], while increased glycerol 3-phosphate labeling may reflect greater allocation of glucose carbon toward membrane lipid synthesis. Thus, the temporal SIRM analysis indicates that TGFβ progressively redirects glucose carbon into biosynthetic pools that support distinct extracellular matrix products and the cellular infrastructure required for their production.

The perturbation studies using galactose and metabolic enzyme inhibitors distinguish metabolic necessity from sufficiency and indicate that individual components of the myofibroblast phenotype have distinct metabolic requirements. Consistent with this concept, Lombardi et al. showed that mitochondrial calcium exchange regulates α-ketoglutarate availability and epigenetic remodeling during fibroblast differentiation, while reduced glycolytic activity diminished αSMA-positive fibroblasts [8]. In the present study, replacing glucose with galactose markedly reduced glycolysis, prevented TGFβ-induced Col1a1 upregulation, and attenuated POSTN induction while preserving αSMA expression. In contrast, GAPDH inhibition with KA prevented induction of Col1a1, POSTN, and αSMA. Similar selectivity has been reported in other fibroblast models: 2-deoxyglucose, PFKFB3 inhibition, and HK2 knockdown differentially affect αSMA, collagen, and fibronectin expression [7], [41], [42], whereas LDH inhibition can suppress glycolysis or lactate production without preventing αSMA fiber formation or fibronectin expression [43]. These interventions therefore should not be considered interchangeable tests of glycolysis. Restricting glucose-supported glycolytic throughput or inhibiting GAPDH differs mechanistically from targeting PFKFB3 [10], PKM isoforms [12], [29], [44], or LDH-dependent lactate production [43], because each perturbation alters a different combination of ATP generation, NAD^+^ regeneration, precursor allocation, metabolite signaling, and noncanonical enzyme functions [45]. Thus, the resulting phenotype depends on both the metabolic node targeted and the fibroblast function measured.

Consistent with this idea, mitochondrial inhibitor studies indicate that matrix production and differentiation marker expression are metabolically distinct outputs. Rotenone and oligomycin markedly reduced Col1a1 abundance while exerting smaller or negligible effects on POSTN and αSMA. This preferential sensitivity of collagen does not necessarily imply that mitochondrial ATP production is limiting, because respiration also supports anaplerosis, amino acid synthesis, redox balance, and metabolite-sensitive signaling. Fibroblast collagen production has been linked to pyruvate carboxylase-dependent anaplerosis [46], glutamine-supported carbon and nitrogen metabolism [47], α-ketoglutarate-dependent collagen translation and proline hydroxylation [48], and proline biosynthesis that helps maintain mitochondrial redox homeostasis [49]. Mitochondrial calcium handling can likewise regulate α-ketoglutarate availability and epigenetic signaling during myofibroblast differentiation [8]. Thus, mitochondrial inhibition could suppress collagen through altered precursor supply, redox state, signaling, or protein processing rather than ATP limitation alone. Consistent with additional non-energetic roles, disruption of Complex III-derived reactive oxygen species attenuates TGFβ-induced profibrotic gene expression without preventing Smad activation [50]. Together, these findings reinforce that matrix production and canonical myofibroblast marker expression have separable metabolic requirements.

The particular sensitivity of collagen production to glycolytic and mitochondrial inhibition may reflect its unusually high biosynthetic demands. Protein translation requires approximately four high-energy phosphate equivalents per amino acid incorporated [51] and has accounted for approximately one-third of cellular ATP turnover in classic mammalian cell estimates [52]. Thus, translation of a type I procollagen heterotrimer containing ∼4300 residues requires a minimum of ∼17,000 high-energy phosphate equivalents [51], [53], [54], [55], before accounting for precursor biosynthesis, post-translational processing, folding, quality control, trafficking, and secretion. Consistent with this burden, galactose substitution and inhibition of GAPDH, Complex I, or ATP synthase each prevented TGFβ-induced COL1A1 accumulation. Collagen synthesis also depends on glycine derived from *de novo* synthesis [13], [15], proline and α-ketoglutarate supplied through glutamine metabolism [47], [49], and extensive ER and secretory processing [56], [57], [58], [59]. Accordingly, glycolytic or mitochondrial inhibition could reduce collagen abundance through altered energy supply, precursor availability, redox balance, translation, processing, or trafficking. Importantly, this metabolic sensitivity may be product-specific. Activated cardiac fibroblasts produce HAS2-dependent high-molecular-weight hyaluronan largely independently of collagen production, proliferation, and migration [4], indicating that distinct ECM products can have separable biosynthetic regulation and metabolic dependencies. Thus, the marked sensitivity of COL1A1 may reflect requirements specific to collagen production rather than a generalized failure of matrix synthesis.

Although these loss-of-metabolic-function studies establish that sufficient glycolytic activity is required for complete myofibroblast differentiation, the gain-of-function experiments indicate that increased glycolysis alone is not sufficient to initiate the phenotype. GLUT1 overexpression increased glycolytic activity in bFGF-treated fibroblasts without inducing myofibroblast markers, demonstrating that enhanced glucose uptake and glycolytic capacity do not independently specify myofibroblast fate in primary murine cardiac fibroblasts. Importantly, this conclusion was reinforced using Glyco^Hi^ overexpression, which increases glycolytic activity by augmenting intracellular levels of fructose-2,6-bisphosphate to activate phosphofructokinase 1 [32], [33]. Glyco^Hi^ overexpression similarly increased glycolysis in bFGF-treated fibroblasts but did not induce myofibroblast markers. Thus, increasing glycolysis through either enhanced glucose transport or downstream stimulation of glycolytic flux is insufficient to drive myofibroblast conversion in the absence of an appropriate profibrotic signal. These complementary gain-of-function approaches argue against the possibility that the negative GLUT1 result simply reflects limitations imposed by downstream glycolytic control and instead support a model in which glycolysis is permissive rather than instructive. These findings suggest that increased glycolytic capacity provides the energetic and biosynthetic resources needed to execute the differentiation program, whereas TGFβ-dependent signaling and transcriptional remodeling supply the cell fate-specific information necessary to initiate and specify the myofibroblast phenotype.

Mitochondrial remodeling emerged later than the early glycolytic response, arguing against a simple Warburg-like shift from oxidative metabolism to glycolysis during myofibroblast differentiation. At 48 h of TGFβ treatment, basal, maximal, and reserve respiratory capacities were increased, and permeabilized cell studies demonstrated greater substrate-supported respiration. This coordinated enhancement of glycolytic and oxidative metabolism is consistent with prior studies of TGFβ-induced fibroblast differentiation [7], [8]. Increased glucose-derived labeling of citrate and malate at 24–48 h further indicates delayed recruitment of glucose-derived carbon into mitochondrial metabolism. Similarly, studies in TGFβ-treated mouse embryonic fibroblasts found substantial glucose-derived citrate labeling but progressively lower labeling of downstream intermediates, with glutamine providing a major carbon contribution to α-ketoglutarate [17]. Our temporal analysis extends these observations by showing that increased glucose contribution to mitochondrial pools becomes most apparent only after glycolytic activation and coincident with increased respiratory capacity. Thus, mitochondrial remodeling may represent a later reinforcing phase that supports the metabolic demands of the differentiated myofibroblast rather than initiating the transition itself.

TGFβ-treated fibroblasts contained smaller, less elongated mitochondria despite little change in representative respiratory complex subunits, suggesting that increased respiratory capacity reflects structural and functional remodeling rather than simply greater abundance of oxidative phosphorylation proteins. One possibility is altered organization of respiratory complexes into higher-order supercomplexes, which can vary with metabolic state and substrate use [60]. Mitochondrial membrane architecture may provide an additional level of regulation because cristae density and organization can influence oxidative capacity and supercomplex assembly [61], [62]. Consistent with increased geometric surface availability, modeling of the observed changes in mitochondrial area and aspect ratio predicted an approximately 10.6% increase in total outer mitochondrial surface area assuming conserved mitochondrial volume; for comparison, idealized division of one sphere into two equal-volume spheres would increase surface area by ∼26%. Although these estimates do not measure inner membrane or cristae area, the observed morphological remodeling could accompany changes in cristae, organelle contacts, or respiratory organization that enhance metabolic capacity. Importantly, mitochondrial dynamics can directly influence fibroblast phenotype and function. Inhibition of Drp1-mediated fission attenuates TGFβ-induced αSMA and collagen expression in renal and corneal fibroblasts [25], [26], whereas excessive fission promotes proliferation and collagen production in right-ventricular fibroblasts in pulmonary hypertension [63]. DRP1/MFF-dependent fission also supports stiffness-directed migration of human lung fibroblasts [64]. Thus, the smaller, less elongated mitochondria observed here may represent functionally relevant remodeling, although it remains to be determined whether Drp1-dependent fission is required for the metabolic and phenotypic changes in cardiac fibroblasts examined here.

Our findings further suggest that metabolism selectively regulates fibroblast functions. KA and rotenone attenuated TGFβ-induced collagen gel contraction, whereas oligomycin unexpectedly increased contraction even in bFGF-treated cells, arguing against a simple requirement for mitochondrial ATP synthesis in force generation. Contractile competence is also not synonymous with αSMA expression, as fibroblast-specific deletion of *Acta2* does not impair cardiac myofibroblast contractility, likely because other actin isoforms compensate for its loss [65]. The oligomycin response similarly illustrates that mechanical function can be dissociated from conventional markers of myofibroblast differentiation. This selectivity is consistent with our prior finding that shifting cardiac fibroblasts from PKM2 toward the more catalytically active PKM1 isoform enhanced contraction [29], suggesting that metabolic configuration can influence force generation. One possible explanation for the oligomycin effect is a compensatory cytoskeletal and glycolytic response, as mitochondrial dysfunction can induce Arp2/3-dependent perimitochondrial actin polymerization and increased extracellular acidification through calcium- and AMPK-dependent signaling [36], [66]. Although this response could contribute to enhanced matrix compaction, collagen gel shrinkage is not a direct measure of actomyosin force and is also influenced by cell number, adhesion, matrix organization, and collagen cross-linking [67], [68], [69]. Direct measurements of cytoskeletal organization and traction force will therefore be needed to help define the mechanism. Nevertheless, the divergent effects of rotenone and oligomycin reinforce that matrix production, marker expression, and contractile behavior have distinct metabolic requirements.

The temporal sequence identified here raises the possibility that metabolic interventions may have strongly context- and stage-dependent effects. Following myocardial infarction, early fibroblast activation and collagen deposition are required for wound stabilization, and restricting glycolytic or other metabolic pathways during this reparative phase could impair scar formation. Interventions aimed at limiting pathological matrix production may therefore be more effective after the initial reparative response, when persistent fibroblast activity contributes increasingly to scar maturation and adverse remodeling. The appropriate therapeutic window is also likely to differ across disease settings; unlike MI, chronic conditions such as aging or pressure overload produce interstitial fibrosis without a discrete injury-and-repair sequence. Thus, the objective may not be to prevent fibroblast activation, but rather to preserve necessary reparative functions while selectively disrupting metabolic processes that sustain excessive matrix production. The increased insulin responsiveness of TGFβ-treated fibroblasts further indicates that metabolic dependencies are shaped by the signaling environment and may couple substrate use to the energetic and biosynthetic demands of repair. Consistent with this concept, prolonged insulin administration after experimental MI accelerated fibroblast accumulation and scar maturation, increased scar thickness, and reduced rupture [70], although systemic administration precludes attribution specifically to fibroblast glycolysis. Because core metabolic pathways are ubiquitous and essential for tissue repair, broad inhibition may have limited therapeutic selectivity. An alternative strategy might target the state- or function-specific organization of metabolism, including local enzyme assemblies that support matrix synthesis, cytoskeletal remodeling, or substrate channeling. Functional glycolytic metabolons have been described on mitochondria, and locally generated glycolytic ATP can support vesicular transport [71], [72], raising the possibility that analogous spatially organized metabolic systems, including metabolic assemblies associated with collagen-translating ribosomes, may operate in fibroblasts. Defining and selectively disrupting such interactions could provide a means of modulating pathological matrix production without broadly suppressing cellular metabolism.

Several limitations should be considered. bFGF was used as an active anti-myofibroblastic comparator rather than a neutral vehicle; thus, the experiments define differences between TGFβ- and bFGF-directed states and cannot completely distinguish TGFβ activation from bFGF-mediated suppression. Nevertheless, bFGF is commonly used to limit culture-induced fibroblast autoactivation [20], [21], [22], [23]. More broadly, primary fibroblasts are highly sensitive to *ex vivo* context. The supraphysiologic rigidity of standard culture substrates can promote spreading, stress-fiber assembly, αSMA expression, matrix production, and mechanical memory, potentially altering fibroblast state and metabolic demand before treatment [73], [74]. Because both ligand conditions shared the same culture history and substrate environment, substrate stiffness alone cannot explain the treatment-dependent differences observed here; nevertheless, culture-induced autoactivation could compress the dynamic range between states and thereby mask or attenuate some metabolic effects of exogenous TGFβ. Mechanical context is also relevant in vivo, where altered cardiomyocyte contractility can generate mechano-sensitized fibroblast states involving matrix-integrin and focal-adhesion signaling [75]. Nutrient composition may similarly create or mask metabolic dependencies; for example, extracellular pyruvate can alter fibroblast sensitivity to glutaminase inhibition [76]. Most mechanistic experiments were performed in male-derived fibroblasts. Although TGFβ-induced marker responses were broadly similar in male- and female-derived cells and major post-MI fibroblast transcriptional programs appear largely shared between sexes [24], these findings do not establish sex independence of the metabolic responses examined here. In addition, fixed-duration ^13^C_6_-glucose labeling reports glucose-carbon incorporation rather than absolute pathway flux and cannot resolve intracellular compartmentation or positional labeling. The metabolic perturbations used here may also have effects beyond their nominal targets. These limitations highlight the need for direct flux measurements and for validation in models of cardiac injury and fibrosis.

Translation of these findings to cardiac fibrosis in vivo will also require consideration of the multicellular environment in which fibroblast activation occurs. Fibroblast phenotype and metabolism are influenced by signals from immune cells, cardiomyocytes, endothelial cells, extracellular matrix, and circulating nutrients, interactions that are incompletely reproduced by isolated cell culture or conventional cardiac organoid systems. Recent studies have demonstrated that macrophage-derived IL-1β can direct profibrotic cardiac fibroblast states in vivo, illustrating how immune-fibroblast communication can modify the response to injury [77], [78]. Consequently, the metabolic dependencies defined here should be tested in models that preserve the temporal and cellular complexity of cardiac injury and repair.

In summary, TGFβ induces a temporally ordered metabolic program in cardiac fibroblasts in which glycolytic activity rises rapidly before broad transcriptional remodeling, followed by increased incorporation of glucose-derived carbon into biosynthetic and mitochondrial pools and greater respiratory capacity as the myofibroblast phenotype develops. Sufficient glycolytic activity is required, but not independently sufficient, for complete differentiation, while perturbation of distinct glycolytic and mitochondrial nodes differentially affects collagen accumulation, marker expression, and matrix contraction. Thus, metabolic remodeling is not simply a downstream consequence of myofibroblast differentiation but an integral, phased component of the differentiation program. Rather than supporting a single binary transition to an “activated” state, metabolism appears to be deployed in phases to enable distinct components of fibroblast differentiation and function.

## 4 Methods

*Animal models*: All animal procedures were performed following the Guide for the Care and Use of Laboratory Animals published by the U.S. National Institutes of Health and were approved by the University of Louisville Institutional Animal Care and Use Committee (Louisville, KY, USA). Male and female C57BL/6J mice (12–20 weeks of age) were purchased from Jackson Laboratories (stock no. 000664) and housed in a pathogen-free facility under a standard 12 h light/dark cycle with *ad libitum* access to food and water.

*Isolation, culture, and treatment of primary cardiac fibroblasts*: Adult mouse cardiac fibroblasts were isolated after collagenase digestion, as in Ref. [79], with slight modifications. Briefly, after euthanasia, the hearts were excised, and the aorta was cannulated using a 23-gauge needle. Ice-cold PBS was perfused through the aorta, and any excess aorta and adipose tissue were carefully excised from the heart before mincing it with a razor blade under sterile conditions. The minced tissue was then digested in a solution of type II collagenase (prepared in sterile PBS, 5000U/heart, Worthington, 46H16739) for 45 min at 37°C with gentle agitation. Collagenase activity was then quenched with culture medium [i.e., Dulbecco's Modified Eagle's Medium/F12 GlutaMax (DMEM, Gibco 10565018) containing 10% fetal bovine serum (FBS, VWR 76324-888), 1% penicillin/streptomycin/amphotericin B (Sigma, A5955), 1% insulin transferrin selenium (ITS-G, Gibco 41400045), and 20 ng/ml human bFGF (Peprotech 100-18B)] and the mixture was then centrifuged at 500g for 10 min. Supernatants were aspirated, and cell pellets were resuspended in culture media before being passed through a 70 μm cell strainer. The cells were then plated in a T-75 cell culture flask (10062-860, VWR) and incubated at 37°C in 5% CO2. Media was changed after 2 h and again after 24 h. For experiments involving ligand stimulation, fibroblasts were treated with either 20 ng/ml bFGF (Peprotech) or 20 ng/ml TGFβ (Peprotech) in the absence of serum at 37°C prior to endpoints. In select experiments, medium was replaced with medium containing galactose (Sigma, G0750) as the only 6-carbon carbohydrate source for the duration of the ligand treatments. Similarly, pharmacological inhibitors of particular steps in glycolysis or mitochondrial metabolism, i.e., koningic acid (Cayman Chemical Company,14079), rotenone (Sigma, R8875), and oligomycin (Sigma, 75351) were added for the duration of treatments. All experiments used cells at passage 1.

Primary cardiac fibroblasts were transduced with recombinant adenoviruses generated and purified by Vector Biolabs (Malvern, PA). The Glyco^Hi^ adenovirus (Ad-pd-PFK2) encodes a mutant form of rat liver 6-phosphofructo-2-kinase/fructose-2,6-bisphosphatase (*Pfkfb1*) containing the S32A and H258A substitutions. This construct was originally generated by site-directed mutagenesis to produce a kinase-active, phosphatase-inactive enzyme, thereby promoting accumulation of fructose-2,6-bisphosphate and increasing glycolytic activity [80], [81], [82], [83]. Adenoviruses encoding mouse *Slc2a1* (ADV-272284) and GFP control were also obtained from Vector Biolabs. For viral titration, fibroblasts were exposed to adenovirus at multiplicities of infection (MOIs) of 50, 100, 250, 500, or 1000 for 24 h, after which virus-containing medium was replaced with fresh culture medium. An MOI of 500 was selected for subsequent experiments with both glycolysis-promoting constructs. For functional studies, fibroblasts were transduced for 24 h, the viral medium was replaced with fresh medium, and cells were subsequently maintained under serum-starved conditions or treated with bFGF (20 ng/mL) or TGFβ (20 ng/mL) for 48 h. Effects on glycolytic activity, mitochondrial respiration, and myofibroblast-marker expression were assessed by extracellular flux analysis and immunoblotting.

*Cell viability assays*: Cell viability following metabolic interventions was assessed by measuring lactate dehydrogenase (LDH) activity in conditioned medium and corresponding cell lysates. After 48 h of treatment, conditioned medium was collected and cells were lysed, and LDH activity was measured in both fractions, essentially as described [84]. For each sample, the fraction of total LDH retained intracellularly was calculated by dividing lysate LDH activity by the sum of LDH activity measured in the conditioned medium and cell lysate. Cell viability was then expressed relative to the corresponding control condition. The control condition was vehicle (DMSO) for experiments involving koningic acid, rotenone, or oligomycin and glucose-containing medium for experiments comparing glucose with galactose. Thus, a value of 100% represents the proportion of intracellularly retained LDH observed in the corresponding control group.

*Mitochondrial imaging*: Cardiac fibroblasts were isolated from C57BL/6J mice as described [29], [85]. The isolated cells were grown in T-75 cell culture flasks (10062–860, VWR) with complete media consisting of DMEM/F12 (Gibco 10-565-018) with 10% FBS (Cytiva SF30088.03), 1% ITS-G (Gibco 41400045), 20 ng/mL bFGF (PeproTech 100-18B) and 1% penicillin/streptomycin/amphotericin B (Sigma-Aldrich A5955) and incubated at 37°C in 5% CO_2_ until confluency. Sterile, 12 mm glass coverslips (Chemglass CLS-1760-012) were placed on 24 well plates (Corning 3526) and coated for 1 h with 1% fibronectin (Sigma-Aldrich F1141) and 0.02% bovine gelatin (Sigma-Aldrich G1393) in DPBS (Sigma-Aldrich D8537) at room temperature. The confluent fibroblasts cultures were trypsinized with 0.25% Trypsin-EDTA (Sigma-Aldrich T4049) and 4000 cells per well were plated and allowed to grow for 24 h in complete media. The cultures were then treated with either TGFβ (20 ng/ml, PeproTech, 100-21C) or bFGF (20 ng/ml, PeproTech, 100-18C) in serum-starved media (DMEM/F12 with 1% penicillin/streptomycin/amphotericin B). After 48 h of bFGF or TGFβ treatment the live cultures were stained with 100 nM MitoTracker™ Red CMXRos (Thermo Fisher Scientific M7512) in culture media for 45 min at 37°C in 5% CO_2_. The cultures subsequently were fixed with 4% paraformaldehyde (EMS15713S) for 15 min and washed 3× with DPBS. Cells were permeabilized with 0.1% Triton X-100 for 15 min and washed again with DPBS 2×. To obtain a proxy for cell boundaries, F-actin cytoskeleton was stained with Alexa Fluor™ 488 Phalloidin (Thermo Fisher A12379; 1:400) in DPBS with 1% BSA for 60 min, and nuclei were stained with 1 μg/mL DAPI (Sigma-Aldrich D9542) in DPBS for 15 min. The coverslips were washed 3× with DPBS, they were then mounted onto glass slides using Prolong™ Glass Antifade Mountant (Invitrogen P36982) and allowed to cure for 1 d at room temperature in the dark.

Cells were imaged using Nikon A1 laser scanning confocal microscope with a Nikon Plan Apo 60×1.4 oil immersion objective. Signals from mitochondria, the F-actin cytoskeleton, and nuclei were excited with 561, 488, and 405 nm lasers, respectively, and emissions were collected using 595/50, 525/50, and 450/50 nm band-pass filters, respectively. Images (1024 × 1024 pixels) were acquired at a lateral resolution of 0.21μm/pixel and an axial resolution of 0.3 μm/pixel. Raw data were optimized with Denoised.ai in NIS-Elements 5 and identical denoising and segmentation parameters were applied to all treatment groups without manual group-specific adjustment. Subsequently, mitochondrial, cellular, and nuclear boundaries were segmented in Fiji/ImageJ using macros and plugins (e.g., Mitochondria Analyzer), allowing each mitochondrion, nucleus, and cell to be individualized as a region of interest (ROI) and performing morphological analysis to describe the shape and distribution of mitochondria. Data were then analyzed and visualized with GraphPad Prism and Python libraries including Matplotlib, NumPy, Pandas, Tkinter, scipy.ndimage and Seaborn.

Referring to calculations presented in the Discussion, mitochondrial surface area was estimated from the mean projected area (A) and aspect ratio (AR; major-to-minor axis ratio) of segmented mitochondria [86], [87]. Mitochondria were modeled as either prolate spheroids or capped cylinders, with dimensions derived from A and AR using standard geometric relationships. For each model, surface area (S), volume (V), and the surface area-to-volume ratio (S/V) were calculated. The relative change in total mitochondrial surface area was then calculated as (S/V)TGFβ/(S/V)bFGF, assuming that total mitochondrial volume per cell remained constant. Using the measured values for bFGF (A = 1.11 μm^2^; AR = 2.48) and TGFβ (A = 0.81 μm^2^; AR = 2.14), this ratio was 1.106 for both the prolate-spheroid and capped-cylinder models, corresponding to an approximately 10.6% increase in total surface area. For comparison, idealized fission of one sphere of radius r into two equal-volume spheres gives each daughter sphere a radius of r/2^(1/3)^. The resulting total surface area is 2 × 4π[r/2^(1/3)^]^2^ = 4πr^2^ × 2^(1/3)^, representing a 1.260-fold, or approximately 26%, increase relative to the original sphere. These estimates assume smooth, rotationally symmetric mitochondria, imaging near the longitudinal plane, and conserved total mitochondrial volume.

*Western Blot Analysis*: Total protein was harvested after washing cells with ice-cold PBS. Cells were scraped in lysis buffer (20 mM HEPES, 110 mM KCl, 1 mM EDTA, 1% IGE-PAL, and 0.1% SDS, pH 7.0) containing protease inhibitor cocktail (Sigma-Aldrich, P8340). The cell lysates were incubated on ice for 30 min and then centrifuged at 20,000g for 20 min. The supernatants were then removed for total protein measurements (Lowry assay) and Western blotting. For conditioned medium experiments, 35 μl of conditioned medium was used for Western blotting. Protein samples were mixed with Laemmli sample buffer (125 mM Tris-HCl, 10% SDS, 50% Glycerol, 0.05% Bromophenol Blue, pH 6.8) and incubated at 95°C for 5 min. Each sample was then loaded with equal protein on a 7.5% acrylamide/bis SDS-PAGE gel for electrophoresis at 120 V. SDS-PAGE-resolved proteins were then transferred overnight onto a PVDF membrane (Cytiva, 10600021) at 4°C at 20 V.Membranes were blocked for 1 h at room temperature with Tris-buffered saline containing Tween-20 (TBS-T) and 5% bovine serum albumin except for GLUT-1 and total OXPHOS which were blocked in milk (BioRad, 1706404). Membranes were probed with primary antibodies overnight at 4°C. The following day, membranes were washed 3× using TBS-T and then incubated with secondary antibody for 1.5 h. Membranes were then washed 3× with TBS-T before exposure to Pierce™ ECL Plus Western Blotting Substrate (Thermo Scientific, 32132) and imaged on a ChemiDoc imager (BioRad). Protein was normalized to total lane protein with an amido black stain. Primary antibodies used included: anti-COL1A1 (1:20,000; Thermo Fisher, PA5-29569), anti-Periostin (1:5000; Millipore, ABT253), anti-α-smooth muscle actin (1:30,000; Cell Signaling, 19245S), anti-GLUT-1 (1:1000 in 3% milk; Millipore, 07-1401), Total OXPHOS (1:500 in 1% milk; Abcam, MS604). The secondary antibody used was HRP-linked anti-rabbit IgG (1:2500; Cell Signaling, 7074) and anti-mouse IgG (1:2500; Cell Signaling, 7076 for Total OXPHOS staining. As a loading control, densitometry values were normalized to amido black (Abcam, ab146311) intensity. For conditioned medium experiments, immunoblot densitometry was normalized to total cell protein.

*Extracellular flux analysis*: Cardiac fibroblasts were seeded in Seahorse XFe96 Cell Culture Microplates (Agilent) at 20,000 cells per well in 100 μL of culture medium and incubated overnight at 37°C in 5% CO_2_. Following the indicated bFGF or TGFβ treatment period, cells were washed and the culture medium was replaced with 180 μL of Seahorse XF DMEM assay medium (Agilent), pH 7.4, supplemented as appropriate for the individual assay. Plates were incubated for 60 min at 37°C in a non-CO_2_ incubator before analysis using an XFe96 Extracellular Flux Analyzer (Agilent). For mitochondrial stress tests, XF DMEM was supplemented with glucose (17.5 mM), glutamine (2.5 mM), and pyruvate (0.5 mM). Following three baseline measurements, oligomycin (1 μg/mL), carbonyl cyanide-p-trifluoromethoxyphenylhydrazone (FCCP; 1 μM), and rotenone plus antimycin A (1 and 10 μM, respectively) were sequentially injected through ports A–C. Basal, ATP-linked, proton-leak, maximal, reserve, and non-mitochondrial respiration were calculated from the resulting oxygen consumption rate (OCR) profiles. For assessment of acute ligand responses, baseline OCR and extracellular acidification rate (ECAR) were recorded before injection of bFGF or TGFβ through port A, followed by continuous measurement of OCR and ECAR. For assessment of insulin-stimulated glycolytic activity after chronic bFGF or TGFβ treatment, glucose was injected to a final concentration of 10 mM, followed by insulin (100 nM) and 2-deoxy-d-glucose (2-DG; 50 mM). For experiments comparing glucose- and galactose-supported metabolism, XF DMEM contained either glucose or galactose at 17.5 mM, as indicated.

Mitochondrial respiratory capacity in permeabilized cardiac fibroblasts was assessed following saponin-mediated plasma membrane permeabilization and provision of defined mitochondrial substrates, essentially as described [88]. OCR and ECAR values were normalized to total protein measured in each well using the Lowry assay. Technical replicate wells were averaged to generate a single value for each independent mouse-derived fibroblast isolation.

*Stable isotope resolved metabolomics*: Analysis of the fate of glucose was performed, essentially as described [28], [32], [89], [90]. Following temporal ligand stimulation (6, 12, 24, 48 h; bFGF or TGFβ), cardiac fibroblasts were incubated at 37°C in DMEM in 6-well plates containing either universally labeled glucose (^13^C_6_-glucose, Cambridge Isotope Laboratories) or unlabeled glucose (17.5 mM) for the final 6 h of the incubation. Following incubation, cells were washed 3× with cold PBS, and quenched by addition of ice-cold acetonitrile:water:chloroform solution at a final ratio of 2:1.5:1. After vortexing, the solution was centrifuged at 18000*g* for 20 min at 4°C. Polar fractions were lyophilized using a Freezone 2.5L −84°C benchtop freeze dryer (Labconco) and reconstituted in 200 μL of LCMS grade water with 0.1% formic acid (v/v) (Thermo Scientific, LS118-1). The samples were vortexed, and centrifuged at 10,000g for 20 min at 4°C, and 75 μL of the supernatant was transferred to a UPLC total recovery glass vial. Next, 5 μL of the sample was injected for liquid chromatography-mass spectrometry (LC-MS) analysis.

*LC-MS analysis*: LC-MS analysis was carried out using Acquity I-Class UPLC system (Waters, MA) coupled to SYNAPT XS Mass Spectrometer (Waters, MA) as described [29], [91]. A reversed phase 2.1 mm × 150 mm Acquity Premier CSH C18 1.7 μm VanGuardT FIT column (Waters, MA) was used for the chromatographic separation with a binary gradient composed of 0.1% formic acid in water (solvent A) and 0.1% formic acid in acetonitrile (Thermo LS120-1, solvent B). The gradient profile started at 0% of B, increased to 7% B over 3.4 min at 0.450 ml/min flow rate, and then increased to 95% B over 6.6 min with a flow rate of 0.600 ml/min. The gradient was held at 95% B for 2.0 min, then returned to the initial conditions over 0.05 min, followed by a 2.95 min re-equilibration before the next injection. The column and sample temperatures were maintained at 60°C and 5°C, respectively.

Quadrupole time-of-flight mass spectrometry (QTOF-MS) data were collected with an electrospray ion source operated in both negative (−) and positive (+) modes. In (−) mode, the capillary voltage was 2.25 kV, the source temperature was 120°C, the desolvation gas flow was 700L/h at a temperature of 650°C, and the cone gas flow was 150 L/h. In (+) mode, the capillary voltage was 0.43 kV, the source temperature was 100°C, the desolvation gas flow was 1000L/h at a temperature of 500°C, and the cone gas flow was 50 L/h. The MS^E^ data independent acquisition (DIA) was performed over the *m*/*z* range of 40−930 Da with low collision energy off (function 1) and high collision energy ramping from 10 to 40 V (function 2) in both ion modes. Each of these functions employed a scan time of 0.2 s. Mass calibration was performed using sodium formate before the sample run, and leucine enkephalin was used as the lock mass solution during the acquisition. After data collection, raw files were imported to UNIFI 1.9 software package (Waters, MA).

*Stable isotope data analyses*. Isotopologue peak deconvolution and assignments were performed using UNIFI 1.9 software package (Waters, MA). Peaks were assigned using a metabolite library first generated and verified using full scan MS and tandem MS (MS/MS) spectra of unlabeled samples [33], [92]. The UNIFI parameters, mass error window and retention time window were kept ±10 mDa and ±0.05 min, respectively for SIRM library matching. The identified peak list and their intensities were exported into a Microsoft Excel (.xlsx) file. All assignments were visually inspected, compared with unlabeled samples and any misassignments were manually deleted, as described [28]. Then, a comma-separated (.csv) file was generated with a table including, compound name, formula, isotope label, and peak intensities of each sample. Finally, the natural isotopic abundance was corrected using the AccuCor algorithm [93] run using R Script (R version 4.3.1 - “Beagle Scouts”). The resulting data were analyzed and plotted with GraphPad Prism (GraphPad Software, San Diego, Ca, USA).

*Gel Contraction Assay*: Fibroblast contractile function was assessed by a collagen contraction assay as described in Ref. [17], with brief modifications. Briefly, 40,000 cardiac fibroblasts were seeded into a 1 mg/mL collagen type I (Corning) gel matrix and cast into a 24 well plate. Following collagen polymerization (20 min at room temperature), the gel was gently released from the edges of the well using a 200 μL pipette tip and media was added containing either 20 ng/mL bFGF (Peprotech) or 20 ng/ml TGFβ (Peprotech) and respective drug treatments. Images were taken 5 d after the gel was released from well edges. ImageJ software (NIH) was used to calculate the surface area.

*Bulk RNA-Sequencing*: Total RNA was isolated from cardiac fibroblasts using the RNeasy micro kit (Qiagen) and cleaned using Min Elute Clean Up Kit (Qiagen). Samples were assessed for purity on the GE Nanovue Plus spectrophotometer, quantitated on Qubit Fluorometer and RNA integrity was assessed on the Agilent 2100 Bioanalyzer with RNA Nano 6000 kit. Libraries were constructed using Illumina Stranded mRNA Library Prep kit and quantified by Qubit Fluorometer using the Qubit 1X dsDNA HS Assay. Kit. Final libraries were normalized to 4 nM with RSB + Tween resuspension buffer, re-quantified, and then equimolar pooled and loaded onto a P4-100 cycle XLEAP cartridge on the NextSeq 2000 platform for sequencing. FASTQ files were generated utilizing BaseSpace DRAGEN analysis Version 4.2.7.

*Bioinformatics Analysis*: Quality control (Q.C.) of the raw sequence data was performed using FastQC (version 0.10.0) for each sequencing sample [94]. The sequences were aligned to the mouse reference genome mm10/GRCm38 using STAR version 2.6 [95]. Differential expression of ENSEMBL protein-coding transcripts was performed using DESeq2 [96], and raw counts were obtained from the STAR-aligned bam format files using HTSeq version 0.10.0 [97]. The raw counts were normalized using the Relative Log Expression (RLE) method and then filtered to exclude genes with fewer than ten counts across the samples. DESeq2 guidelines were used to identify differentially expressed genes, and all P values were adjusted for testing multiple genes (Benjamini–Hochberg procedure; p ≤ 0.05). Functional enrichment analysis was performed using the clusterProfiler R package to identify enriched Gene Ontology biological processes for each set of differentially expressed genes (DEGs) [98]. DEGs were defined as genes with q value < 0.05. Enriched biological processes were retained if they had a gene count ≥5 and q value < 0.05. RNA-seq data were deposited in the NCBI GEO database under Accession Number GSE343789.

*Statistical analysis*: Unless otherwise indicated, the biological replicate was an independent cardiac fibroblast isolation obtained from a different mouse. Technical replicates, including replicate wells in extracellular flux assays, were averaged to generate a single value for each biological replicate before statistical analysis. Data are presented as mean ± SD, and the number of independent biological replicates (n) is indicated in the corresponding figure legends. Statistical analyses of non-transcriptomic data were performed using GraphPad Prism version 10 (GraphPad Software). For comparisons between two independent groups, two-tailed unpaired Student's t-tests were used. Paired Student's t-tests were used when experimental conditions were applied to cells derived from the same fibroblast isolation, as indicated for the permeabilized cell respiration studies. For experiments containing two experimental factors, such as ligand treatment and metabolic intervention, two-way ANOVA was performed followed by Šídák's multiple comparisons test for prespecified pairwise comparisons. Repeated-measures two-way ANOVA was used when multiple conditions or time points were assessed within the same biological replicate, followed by Šídák's multiple-comparisons test. For experiments containing more than two groups defined by a single factor, one-way ANOVA followed by Šídák's multiple-comparisons test was used. Welch's unpaired *t*-test was used where specifically indicated. Exact statistical tests are provided in the corresponding figure legends. Statistical significance was defined as p < 0.05. For stable isotope-resolved metabolomics, relative metabolite abundances were normalized to total cellular protein before statistical analysis. Total fractional ^13^C enrichment values, which are bounded between 0 and 1, were transformed using the logit transformation before hypothesis testing. Treatment and time effects were analyzed by repeated measures two-way ANOVA followed by Šídák's multiple-comparisons test. Statistical significance displayed on isotopologue-distribution plots reflects analysis of total fractional enrichment rather than separate statistical testing of individual isotopologue fractions. RNA-sequencing data were analyzed separately using DESeq2. Differentially expressed genes were identified using Benjamini–Hochberg adjustment for multiple testing and were considered significant at an adjusted q value < 0.05. Gene Ontology Biological Process enrichment was performed using clusterProfiler, with enriched terms retained at q < 0.05 and a minimum gene count of five.

## CRediT authorship contribution statement

**Collin K. Wells:** Writing – review & editing, Writing – original draft, Methodology, Investigation, Funding acquisition, Formal analysis, Conceptualization. **Lianay Gutierrez Luque:** Writing – review & editing, Writing – original draft, Methodology, Investigation, Formal analysis, Conceptualization. **Daniel C. Nguyen:** Writing – review & editing, Writing – original draft, Methodology, Investigation, Funding acquisition, Formal analysis, Conceptualization. **Madison S. Teer:** Investigation, Formal analysis. **Ning Chen:** Writing – review & editing, Investigation, Formal analysis. **Yania Martinez-Ondaro:** Investigation, Formal analysis. **Pawel K. Lorkiewicz:** Writing – review & editing, Formal analysis, Data curation. **Richa Singhal:** Writing – review & editing, Writing – original draft, Methodology, Investigation, Formal analysis, Data curation, Conceptualization. **Maleesha De Silva:** Writing – review & editing, Investigation, Formal analysis, Data curation. **Bradford G. Hill:** Writing – review & editing, Writing – original draft, Supervision, Project administration, Methodology, Investigation, Funding acquisition, Formal analysis, Conceptualization.

## Ethics declaration

This study was conducted in accordance with the ARRIVE (Animal Research: Reporting of In Vivo Experiments) guidelines. This study was approved by the University of Louisville Institutional Animal Care and Use Commitee.

## Declaration of generative AI and AI-assisted technologies in the manuscript preparation process

During the preparation of this work, the authors used ChatGPT (OpenAI) to refine language for grammar and syntax, to check for mistakes, and to draft the Graphical Abstract, which was subsequently re-made in BioRender [Created in BioRender. Gutierrez, L. (2026) https://BioRender.com/dsq53p9]. After using this tool/service, the authors reviewed and edited the content for accuracy and take full responsibility for the content of the published article.

## Declaration of competing interest

The authors declare that they have no known competing financial interests or personal relationships that could have appeared to influence the work reported in this paper.

## Data Availability

The RNA-sequencing data generated in this study have been deposited in the NCBI Gene Expression Omnibus (GEO) under accession number GSE343789. The stable isotope-resolved metabolomics (SIRM) data generated in this study have been deposited in the NIH Common Fund’s National Metabolomics Data Repository (NMDR), Metabolomics Workbench (Sud et al., 2016; PMID: 26467476), under Study ID ST005266 (Project ID PR003402). The authors acknowledge the Metabolomics Workbench, which is supported by NIH grants U2C-DK119886 and OT2-OD030544. All other data supporting the findings of this study are included within the article and its Supplementary Materials or are available from the corresponding author upon reasonable request.

## References

[1] Breul S.D., Bradley K.H., Hance A.J., Schafer M.P., Berg R.A., Crystal R.G. (1980). Control of collagen production by human diploid lung fibroblasts. J. Biol. Chem..

[2] Tolstoshev P., Berg R.A., Rennard S.I., Bradley K.H., Trapnell B.C., Crystal R.G. (1981). Procollagen production and procollagen messenger RNA levels and activity in human lung fibroblasts during periods of rapid and stationary growth. J. Biol. Chem..

[3] Green H., Hemerman D. (1964). Production of hyaluronate and collagen by fibroblast clones in culture. Nature.

[4] Little D.T., Howard C.M., Pendergraft E., Brittian K.R., Audam T.N., Lukudu E.W., Smith J., Nguyen D., Nishida Y., Yamaguchi Y., Brainard R.E., Singhal R.A., Jones S.P. (2025). Activated cardiac fibroblasts are a primary source of high-molecular-weight hyaluronan production. Am. J. Physiol.: Cell Physiol..

[5] Henderson J., Duffy L., Stratton R., Ford D., O'Reilly S. (2020). Metabolic reprogramming of glycolysis and glutamine metabolism are key events in myofibroblast transition in systemic sclerosis pathogenesis. J. Cell Mol. Med..

[6] Ding H., Jiang L., Xu J., Bai F., Zhou Y., Yuan Q., Luo J., Zen K., Yang J. (2017). Inhibiting aerobic glycolysis suppresses renal interstitial fibroblast activation and renal fibrosis. Am. J. Physiol. Ren. Physiol..

[7] Bernard K., Logsdon N.J., Ravi S., Xie N., Persons B.P., Rangarajan S., Zmijewski J.W., Mitra K., Liu G., Darley-Usmar V.M., Thannickal V.J. (2015). Metabolic reprogramming is required for myofibroblast contractility and differentiation. J. Biol. Chem..

[8] Lombardi A.A., Gibb A.A., Arif E., Kolmetzky D.W., Tomar D., Luongo T.S., Jadiya P., Murray E.K., Lorkiewicz P.K., Hajnoczky G., Murphy E., Arany Z.P., Kelly D.P., Margulies K.B., Hill B.G., Elrod J.W. (2019). Mitochondrial calcium exchange links metabolism with the epigenome to control cellular differentiation. Nat. Commun..

[9] Chen W., Zhang J., Zhong W., Liu Y., Lu Y., Zeng Z., Huang H., Wan X., Meng X., Zou F., Cai S., Dong H. (2021). Anlotinib inhibits PFKFB3-Driven glycolysis in myofibroblasts to reverse pulmonary fibrosis. Front. Pharmacol..

[10] Xie N., Tan Z., Banerjee S., Cui H., Ge J., Liu R.M., Bernard K., Thannickal V.J., Liu G. (2015). Glycolytic reprogramming in myofibroblast differentiation and lung fibrosis. Am. J. Respir. Crit. Care Med..

[11] Vigetti D., Viola M., Karousou E., De Luca G., Passi A. (2014). Metabolic control of hyaluronan synthases. Matrix Biol..

[12] Satyanarayana G., Turaga R.C., Sharma M., Wang S., Mishra F., Peng G., Deng X., Yang J., Liu Z.R. (2021). Pyruvate kinase M2 regulates fibrosis development and progression by controlling glycine auxotrophy in myofibroblasts. Theranostics.

[13] Selvarajah B., Azuelos I., Plate M., Guillotin D., Forty E.J., Contento G., Woodcock H.V., Redding M., Taylor A., Brunori G., Durrenberger P.F., Ronzoni R., Blanchard A.D., Mercer P.F., Anastasiou D., Chambers R.C. (2019). mTORC1 amplifies the ATF4-dependent de novo serine-glycine pathway to supply glycine during TGF-beta(1)-induced collagen biosynthesis. Sci. Signal..

[14] Hamanaka R.B., Nigdelioglu R., Meliton A.Y., Tian Y., Witt L.J., O'Leary E., Sun K.A., Woods P.S., Wu D., Ansbro B., Ard S., Rohde J.M., Dulin N.O., Guzy R.D., Mutlu G.M. (2018). Inhibition of phosphoglycerate dehydrogenase attenuates bleomycin-induced pulmonary fibrosis. Am. J. Respir. Cell Mol. Biol..

[15] Nigdelioglu R., Hamanaka R.B., Meliton A.Y., O'Leary E., Witt L.J., Cho T., Sun K., Bonham C., Wu D., Woods P.S., Husain A.N., Wolfgeher D., Dulin N.O., Chandel N.S., Mutlu G.M. (2016). Transforming Growth Factor (TGF)-beta Promotes de Novo Serine Synthesis for Collagen Production. J. Biol. Chem..

[16] Gibb A.A., Murray E.K., Huynh A.T., Gaspar R.B., Ploesch T.L., Bedi K., Lombardi A.A., Lorkiewicz P.K., Roy R., Arany Z., Kelly D.P., Margulies K.B., Hill B.G., Elrod J.W. (2022). Glutaminolysis is essential for myofibroblast persistence and in vivo targeting reverses fibrosis and cardiac dysfunction in heart failure. Circulation.

[17] Gibb A.A., Huynh A.T., Gaspar R.B., Ploesch T.L., Lombardi A.A., Lorkiewicz P.K., Lazaropoulos M.P., Bedi K., Arany Z., Margulies K.B., Hill B.G., Elrod J.W. (2022). Glutamine uptake and catabolism is required for myofibroblast formation and persistence. J. Mol. Cell. Cardiol..

[18] Gibb A.A., Lazaropoulos M.P., Elrod J.W. (2020). Myofibroblasts and fibrosis: mitochondrial and metabolic control of cellular differentiation. Circ. Res..

[19] Guillard J., Schworer S. (2024). Metabolic control of collagen synthesis. Matrix Biol..

[20] Liguori T.T.A., Liguori G.R., Moreira L.F.P., Harmsen M.C. (2018). Fibroblast growth factor-2, but not the adipose tissue-derived stromal cells secretome, inhibits TGF-beta1-induced differentiation of human cardiac fibroblasts into myofibroblasts. Sci. Rep..

[21] Narine K., De Wever O., Van Valckenborgh D., Francois K., Bracke M., DeSmet S., Mareel M., Van Nooten G. (2006). Growth factor modulation of fibroblast proliferation, differentiation, and invasion: implications for tissue valve engineering. Tissue Eng..

[22] Desai V.D., Hsia H.C., Schwarzbauer J.E. (2014). Reversible modulation of myofibroblast differentiation in adipose-derived mesenchymal stem cells. PLoS One.

[23] Grotendorst G.R., Rahmanie H., Duncan M.R. (2004). Combinatorial signaling pathways determine fibroblast proliferation and myofibroblast differentiation. FASEB J..

[24] Singhal R., Ferrari I., Brainard R.E., Brittian K.R., Chariker J., Collins H.E., Moore J.B.t., Nong Y., Jones S.P. (2026). Temporal dynamics in Murine cardiac transcriptome following myocardial infarction. Circ. Res..

[25] Wang Y., Lu M., Xiong L., Fan J., Zhou Y., Li H., Peng X., Zhong Z., Wang Y., Huang F., Chen W., Yu X., Mao H. (2020). Drp1-mediated mitochondrial fission promotes renal fibroblast activation and fibrogenesis. Cell Death Dis..

[26] Jeon K.I., Kumar A., Wozniak K.T., Nehrke K., Huxlin K.R. (2022). Defining the role of mitochondrial fission in corneal myofibroblast differentiation. Investig. Ophthalmol. Vis. Sci..

[27] Tong Z., Du X., Zhou Y., Jing F., Ma J., Feng Y., Lou S., Wang Q., Dong Z. (2024). Drp1-mediated mitochondrial fission promotes pulmonary fibrosis progression through the regulation of lipid metabolic reprogramming by ROS/HIF-1alpha. Cell. Signal..

[28] Lorkiewicz P.K., Gibb A.A., Rood B.R., He L., Zheng Y., Clem B.F., Zhang X., Hill B.G. (2019). Integration of flux measurements and pharmacological controls to optimize stable isotope-resolved metabolomics workflows and interpretation. Sci. Rep..

[29] Wells C.K., Nguyen D.C., Brainard R.E., McNally L.A., De Silva M., Brittian K.R., Garrett L., Taylor M.S., Martinez-Ondaro Y., Howard C., Suluru S., Dassanayaka S., Mohamed T.M.A., Singhal R., Gibb A.A., Lorkiewicz P.K., Moore J.B.t., Jones S.P., Hill B.G. (2025). Pyruvate kinase splice variants in fibroblasts influence cardiac remodeling after myocardial infarction in male mice. J. Mol. Cell. Cardiol..

[30] Coelho A.I., Berry G.T., Rubio-Gozalbo M.E. (2015). Galactose metabolism and health. Curr. Opin. Clin. Nutr. Metab. Care.

[31] Sakai K., Hasumi K., Endo A. (1991). Identification of koningic acid (heptelidic acid)-modified site in rabbit muscle glyceraldehyde-3-phosphate dehydrogenase. Biochim. Biophys. Acta.

[32] Gibb A.A., Lorkiewicz P.K., Zheng Y.T., Zhang X., Bhatnagar A., Jones S.P., Hill B.G. (2017). Integration of flux measurements to resolve changes in anabolic and catabolic metabolism in cardiac myocytes. Biochem. J..

[33] Salabei J.K., Lorkiewicz P.K., Mehra P., Gibb A.A., Haberzettl P., Hong K.U., Wei X., Zhang X., Li Q., Wysoczynski M., Bolli R., Bhatnagar A., Hill B.G. (2016). Type 2 diabetes dysregulates glucose metabolism in cardiac progenitor cells. J. Biol. Chem..

[34] Sun Y., Weber K.T. (2000). Infarct scar: a dynamic tissue. Cardiovasc. Res..

[35] Vallee A., Lecarpentier Y. (2019). TGF-beta in fibrosis by acting as a conductor for contractile properties of myofibroblasts. Cell Biosci..

[36] Chakrabarti R., Fung T.S., Kang T., Elonkirjo P.W., Suomalainen A., Usherwood E.J., Higgs H.N. (2022). Mitochondrial dysfunction triggers actin polymerization necessary for rapid glycolytic activation. J. Cell Biol..

[37] Sandbo N., Dulin N. (2011). Actin cytoskeleton in myofibroblast differentiation: ultrastructure defining form and driving function. Transl. Res. : J. Lab. Clin. Med..

[38] Zhou M.Y., Cheng M.L., Huang T., Hu R.H., Zou G.L., Li H., Zhang B.F., Zhu J.J., Liu Y.M., Liu Y., Zhao X.K. (2021). Transforming growth factor beta-1 upregulates glucose transporter 1 and glycolysis through canonical and noncanonical pathways in hepatic stellate cells. World J. Gastroenterol..

[39] Tzavlaki K., Moustakas A. (2020). TGF-beta signaling. Biomolecules.

[40] O'Leary E.M., Tian Y., Nigdelioglu R., Witt L.J., Cetin-Atalay R., Meliton A.Y., Woods P.S., Kimmig L.M., Sun K.A., Gokalp G.A., Mutlu G.M., Hamanaka R.B. (2020). TGF-beta promotes metabolic reprogramming in lung fibroblasts via mTORC1-dependent ATF4 activation. Am. J. Respir. Cell Mol. Biol..

[41] Wang F., Yin X., Fan Y.M., Zhang X., Ma C., Jia K., Zhou W., Tang Z., Qi L.W., Li J. (2023). Upregulation of glycolytic enzyme PFKFB3 by deubiquitinase OTUD4 promotes cardiac fibrosis post myocardial infarction. J. Mol. Med. (Berl.).

[42] Chen Z.T., Gao Q.Y., Wu M.X., Wang M., Sun R.L., Jiang Y., Guo Q., Guo D.C., Liu C.Y., Chen S.X., Liu X., Wang J.F., Zhang H.F., Chen Y.X. (2021). Glycolysis inhibition alleviates cardiac fibrosis after myocardial infarction by suppressing cardiac fibroblast activation. Front. Cardiovasc. Med..

[43] Schruf E., Schroeder V., Kuttruff C.A., Weigle S., Krell M., Benz M., Bretschneider T., Holweg A., Schuler M., Frick M., Nicklin P., Garnett J.P., Sobotta M.C. (2019). Human lung fibroblast-to-myofibroblast transformation is not driven by an LDH5-dependent metabolic shift towards aerobic glycolysis. Respir. Res..

[44] Zhang H., Wang D., Li M., Plecita-Hlavata L., D'Alessandro A., Tauber J., Riddle S., Kumar S., Flockton A., McKeon B.A., Frid M.G., Reisz J.A., Caruso P., El Kasmi K.C., Jezek P., Morrell N.W., Hu C.J., Stenmark K.R. (2017). Metabolic and proliferative state of vascular adventitial fibroblasts in pulmonary hypertension is regulated through a MicroRNA-124/PTBP1 (polypyrimidine tract binding protein 1)/Pyruvate kinase muscle axis. Circulation.

[45] Turner E., Roda G., Londono P., Herrera J.A., Evans C.M., Rangarajan S. (2024). Inhibition of distal glycolytic enzymes suppresses fibroblast activation without deleterious bioenergetic effects. Am. J. Respir. Cell Mol. Biol..

[46] Schworer S., Pavlova N.N., Cimino F.V., King B., Cai X., Sizemore G.M., Thompson C.B. (2021). Fibroblast pyruvate carboxylase is required for collagen production in the tumour microenvironment. Nat. Metab..

[47] Hamanaka R.B., O'Leary E.M., Witt L.J., Tian Y., Gokalp G.A., Meliton A.Y., Dulin N.O., Mutlu G.M. (2019). Glutamine metabolism is required for collagen protein synthesis in lung fibroblasts. Am. J. Respir. Cell Mol. Biol..

[48] Ge J., Cui H., Xie N., Banerjee S., Guo S., Dubey S., Barnes S., Liu G. (2018). Glutaminolysis promotes collagen translation and stability via alpha-ketoglutarate-mediated mTOR activation and proline hydroxylation. Am. J. Respir. Cell Mol. Biol..

[49] Schworer S., Berisa M., Violante S., Qin W., Zhu J., Hendrickson R.C., Cross J.R., Thompson C.B. (2020). Proline biosynthesis is a vent for TGFbeta-induced mitochondrial redox stress. EMBO J..

[50] Jain M., Rivera S., Monclus E.A., Synenki L., Zirk A., Eisenbart J., Feghali-Bostwick C., Mutlu G.M., Budinger G.R., Chandel N.S. (2013). Mitochondrial reactive oxygen species regulate transforming growth factor-beta signaling. J. Biol. Chem..

[51] Stouthamer A.H. (1973). A theoretical study on the amount of ATP required for synthesis of microbial cell material. Antonie Leeuwenhoek.

[52] Buttgereit F., Brand M.D. (1995). A hierarchy of ATP-consuming processes in mammalian cells. Biochem. J..

[53] Chu M.L., de Wet W., Bernard M., Ding J.F., Morabito M., Myers J., Williams C., Ramirez F. (1984). Human pro alpha 1(I) collagen gene structure reveals evolutionary conservation of a pattern of introns and exons. Nature.

[54] Tromp G., Kuivaniemi H., Stacey A., Shikata H., Baldwin C.T., Jaenisch R., Prockop D.J. (1988). Structure of a full-length cDNA clone for the prepro alpha 1(I) chain of human type I procollagen. Biochem. J..

[55] Kuivaniemi H., Tromp G., Chu M.L., Prockop D.J. (1988). Structure of a full-length cDNA clone for the prepro alpha 2(I) chain of human type I procollagen. Comparison with the chicken gene confirms unusual patterns of gene conservation. Biochem. J..

[56] De Giorgi F., Fumagalli M., Scietti L., Forneris F. (2021). Collagen hydroxylysine glycosylation: non-conventional substrates for atypical glycosyltransferase enzymes. Biochem. Soc. Trans..

[57] Omari S., Makareeva E., Gorrell L., Jarnik M., Lippincott-Schwartz J., Leikin S. (2020). Mechanisms of procollagen and HSP47 sorting during ER-to-Golgi trafficking. Matrix Biol..

[58] Malhotra V., Erlmann P. (2015). The pathway of collagen secretion. Annu. Rev. Cell Dev. Biol..

[59] Kruse N.J., Bornstein P. (1975). The metabolic requirements for transcellular movement and secretion of collagen. J. Biol. Chem..

[60] Zheng Y., Gibb A.A., Xu H., Liu S., Hill B.G. (2023). The metabolic state of the heart regulates mitochondrial supercomplex abundance in mice. Redox Biol..

[61] Nielsen J., Gejl K.D., Hey-Mogensen M., Holmberg H.C., Suetta C., Krustrup P., Elemans C.P.H., Ortenblad N. (2017). Plasticity in mitochondrial cristae density allows metabolic capacity modulation in human skeletal muscle. The Journal of physiology.

[62] Cogliati S., Frezza C., Soriano M.E., Varanita T., Quintana-Cabrera R., Corrado M., Cipolat S., Costa V., Casarin A., Gomes L.C., Perales-Clemente E., Salviati L., Fernandez-Silva P., Enriquez J.A., Scorrano L. (2013). Mitochondrial cristae shape determines respiratory chain supercomplexes assembly and respiratory efficiency. Cell.

[63] Tian L., Potus F., Wu D., Dasgupta A., Chen K.H., Mewburn J., Lima P., Archer S.L. (2018). Increased Drp1-Mediated mitochondrial fission promotes proliferation and collagen production by right ventricular fibroblasts in experimental pulmonary arterial hypertension. Front. Physiol..

[64] Guo T., Jiang C.S., Yang S.Z., Zhu Y., He C., Carter A.B., Antony V.B., Peng H., Zhou Y. (2023). Mitochondrial fission and bioenergetics mediate human lung fibroblast durotaxis. JCI Insight.

[65] Li Y., Li C., Liu Q., Wang L., Bao A.X., Jung J.P., Dodlapati S., Sun J., Gao P., Zhang X., Francis J., Molkentin J.D., Fu X. (2022). Loss of Acta2 in cardiac fibroblasts does not prevent the myofibroblast differentiation or affect the cardiac repair after myocardial infarction. J. Mol. Cell. Cardiol..

[66] Fung T.S., Chakrabarti R., Kollasser J., Rottner K., Stradal T.E.B., Kage F., Higgs H.N. (2022). Parallel kinase pathways stimulate actin polymerization at depolarized mitochondria. Curr. Biol..

[67] Redden R.A., Doolin E.J. (2003). Collagen crosslinking and cell density have distinct effects on fibroblast-mediated contraction of collagen gels. Skin Res. Technol..

[68] Zhu Y.K., Umino T., Liu X.D., Wang H.J., Romberger D.J., Spurzem J.R., Rennard S.I. (2001). Contraction of fibroblast-containing collagen gels: initial collagen concentration regulates the degree of contraction and cell survival, in vitro cellular & developmental biology. Animal.

[69] Thoumine O., Ott A. (1996). Influence of adhesion and cytoskeletal integrity on fibroblast traction. Cell Motil Cytoskeleton.

[70] Gudbjarnason S., Fenton J.C., Wolf P.L., Bing R.J. (1966). Stimulation of reparative processes following experimental myocardial infarction. Arch. Intern. Med..

[71] Wang H., Vant J.W., Zhang A., Sanchez R.G., Wu Y., Micou M.L., Luczak V., Whiddon Z., Carlson N.M., Yu S.B., Jabbo M., Yoon S., Abushawish A.A., Ghassemian M., Masubuchi T., Gan Q., Watanabe S., Griffis E.R., Hammarlund M., Singharoy A., Pekkurnaz G. (2024). Organization of a functional glycolytic metabolon on mitochondria for metabolic efficiency. Nat. Metab..

[72] Zala D., Hinckelmann M.V., Yu H., Lyra da Cunha M.M., Liot G., Cordelieres F.P., Marco S., Saudou F. (2013). Vesicular glycolysis provides on-board energy for fast axonal transport. Cell.

[73] Felisbino M.B., Rubino M., Travers J.G., Schuetze K.B., Lemieux M.E., Anseth K.S., Aguado B.A., McKinsey T.A. (2024). Substrate stiffness modulates cardiac fibroblast activation, senescence, and proinflammatory secretory phenotype. Am. J. Physiol. Heart Circ. Physiol..

[74] Balestrini J.L., Chaudhry S., Sarrazy V., Koehler A., Hinz B. (2012). The mechanical memory of lung myofibroblasts. Integr. Biol..

[75] Bretherton R.C., Reichardt I.M., Zabrecky K.A., Nagle A., Bailey L.R.J., Bugg D., Smolgovsky S., Gifford A.L., McMillen T.S., Goldstein A.J., Kooiker K.B., Flint G.V., Martinson A., Gunaje J., Koser F., Plaster E., Linke W.A., Regnier M., Moussavi-Harami F., Sniadecki N.J., DeForest C.A., Davis J. (2025). Preventing hypocontractility-induced fibroblast expansion alleviates dilated cardiomyopathy. Science.

[76] Contento G., Wilson J.A., Selvarajah B., Plate M., Guillotin D., Morales V., Trevisani M., Pitozzi V., Bianchi K., Chambers R.C. (2024). Pyruvate metabolism dictates fibroblast sensitivity to GLS1 inhibition during fibrogenesis. JCI Insight.

[77] Alexanian M., Padmanabhan A., Nishino T., Travers J.G., Ye L., Pelonero A., Lee C.Y., Sadagopan N., Huang Y., Auclair K., Zhu A., An Y., Ekstrand C.A., Martinez C., Teran B.G., Flanigan W.R., Kim C.K., Lumbao-Conradson K., Gardner Z., Li L., Costa M.W., Jain R., Charo I., Combes A.J., Haldar S.M., Pollard K.S., Vagnozzi R.J., McKinsey T.A., Przytycki P.F., Srivastava D. (2024). Chromatin remodelling drives immune cell-fibroblast communication in heart failure. Nature.

[78] Amrute J.M., Luo X., Penna V., Yang S., Yamawaki T., Hayat S., Bredemeyer A., Jung I.H., Kadyrov F.F., Heo G.S., Venkatesan R., Shi S.Y., Parvathaneni A., Koenig A.L., Kuppe C., Baker C., Luehmann H., Jones C., Kopecky B., Zeng X., Bleckwehl T., Ma P., Lee P., Terada Y., Fu A., Furtado M., Kreisel D., Kovacs A., Stitziel N.O., Jackson S., Li C.M., Liu Y., Rosenthal N.A., Kramann R., Ason B., Lavine K.J. (2024). Targeting immune-fibroblast cell communication in heart failure. Nature.

[79] Sadri G., Fischer A.G., Brittian K.R., Elliott E., Nystoriak M.A., Uchida S., Wysoczynski M., Leask A., Jones S.P., Moore J.B.t. (2022). Collagen type XIX regulates cardiac extracellular matrix structure and ventricular function. Matrix Biol..

[80] Wang Q., Donthi R.V., Wang J., Lange A.J., Watson L.J., Jones S.P., Epstein P.N. (2008). Cardiac phosphatase-deficient 6-phosphofructo-2-kinase/fructose-2,6-bisphosphatase increases glycolysis, hypertrophy, and myocyte resistance to hypoxia. Am. J. Physiol. Heart Circ. Physiol..

[81] Argaud D., Lange A.J., Becker T.C., Okar D.A., el-Maghrabi M.R., Newgard C.B., Pilkis S.J. (1995). Adenovirus-mediated overexpression of liver 6-phosphofructo-2-kinase/fructose-2,6-bisphosphatase in gluconeogenic rat hepatoma cells. Paradoxical effect on Fru-2,6-P2 levels. J. Biol. Chem..

[82] Kurland I.J., el-Maghrabi M.R., Correia J.J., Pilkis S.J. (1992). Rat liver 6-phosphofructo-2-kinase/fructose-2,6-bisphosphatase. Properties of phospho- and dephospho- forms and of two mutants in which Ser32 has been changed by site-directed mutagenesis. J. Biol. Chem..

[83] Tauler A., Lin K., Pilkis S.J. (1990). Hepatic 6-phosphofructo-2-kinase/fructose-2,6-bisphosphatase. Use of site-directed mutagenesis to evaluate the roles of His-258 and His-392 in catalysis. J. Biol. Chem..

[84] Perez J., Hill B.G., Benavides G.A., Dranka B.P., Darley-Usmar V.M. (2010). Role of cellular bioenergetics in smooth muscle cell proliferation induced by platelet-derived growth factor. Biochem. J..

[85] Nguyen D.C., Wells C.K., Taylor M.S., Martinez-Ondaro Y., Singhal R., Brittian K.R., Brainard R.E., Moore J.B., Hill B.G. (2025). Dietary branched-chain amino acids modify postinfarct cardiac remodeling and function in the murine heart. J. Am. Heart Assoc..

[86] Koopman W.J., Visch H.J., Smeitink J.A., Willems P.H. (2006). Simultaneous quantitative measurement and automated analysis of mitochondrial morphology, mass, potential, and motility in living human skin fibroblasts. Cytometry A.

[87] Nikolaisen J., Nilsson L.I., Pettersen I.K., Willems P.H., Lorens J.B., Koopman W.J., Tronstad K.J. (2014). Automated quantification and integrative analysis of 2D and 3D mitochondrial shape and network properties. PLoS One.

[88] Salabei J.K., Gibb A.A., Hill B.G. (2014). Comprehensive measurement of respiratory activity in permeabilized cells using extracellular flux analysis. Nat. Protoc..

[89] Abouleisa R.R.E., McNally L., Salama A.B.M., Hammad S.K., Ou Q., Wells C., Lorkiewicz P.K., Bolli R., Mohamed T.M.A., Hill B.G. (2021). Cell cycle induction in human cardiomyocytes is dependent on biosynthetic pathway activation. Redox Biol..

[90] Mehra P., Guo Y., Nong Y., Lorkiewicz P., Nasr M., Li Q., Muthusamy S., Bradley J.A., Bhatnagar A., Wysoczynski M., Bolli R., Hill B.G. (2018). Cardiac mesenchymal cells from diabetic mice are ineffective for cell therapy-mediated myocardial repair. Basic Res. Cardiol..

[91] Jobe T., Stephan J., Wells C.K., De Silva M., Lorkiewicz P.K., Hill B.G., Wysoczynski M. (2025). Phase partitioning of the neutrophil oxidative burst is coordinated by accessory pathways of glucose metabolism and mitochondrial activity. J. Biol. Chem..

[92] Wei X., Lorkiewicz P.K., Shi B., Salabei J.K., Hill B.G., Kim S., McClain C.J., Zhang X. (2017). Analysis of stable isotope assisted metabolomics data acquired by high resolution mass spectrometry. Anal. Methods.

[93] Su X., Lu W., Rabinowitz J.D. (2017). Metabolite spectral accuracy on orbitraps. Anal. Chem..

[94] Andrews S. (2014). FastQC: a quality control tool for high throughput sequence data. http://www.bioinformatics.babraham.ac.uk/projects/fastqc/.

[95] Dobin A., Davis C.A., Schlesinger F., Drenkow J., Zaleski C., Jha S., Batut P., Chaisson M., Gingeras T.R. (2013). STAR: ultrafast universal RNA-seq aligner. Bioinformatics.

[96] Love M.I., Huber W., Anders S. (2014). Moderated estimation of fold change and dispersion for RNA-seq data with DESeq2. Genome Biol..

[97] Anders S., Pyl P.T., Huber W. (2015). HTSeq--a Python framework to work with high-throughput sequencing data. Bioinformatics.

[98] Yu G., Wang L.G., Han Y., He Q.Y. (2012). clusterProfiler: an R package for comparing biological themes among gene clusters. OMICS.

