# Supplementary material for: Phasic metabolic remodeling coordinates cardiac myofibroblast differentiation and function
Source: Redox Biol. 2026 Oct 16;98:104441. doi: 10.1016/j.redox.2026.104441 (PMC13695315; doi:10.1016/j.redox.2026.104441)
Supplement: Supplementary file 1 [file mmc1.pdf]

Figure 1A

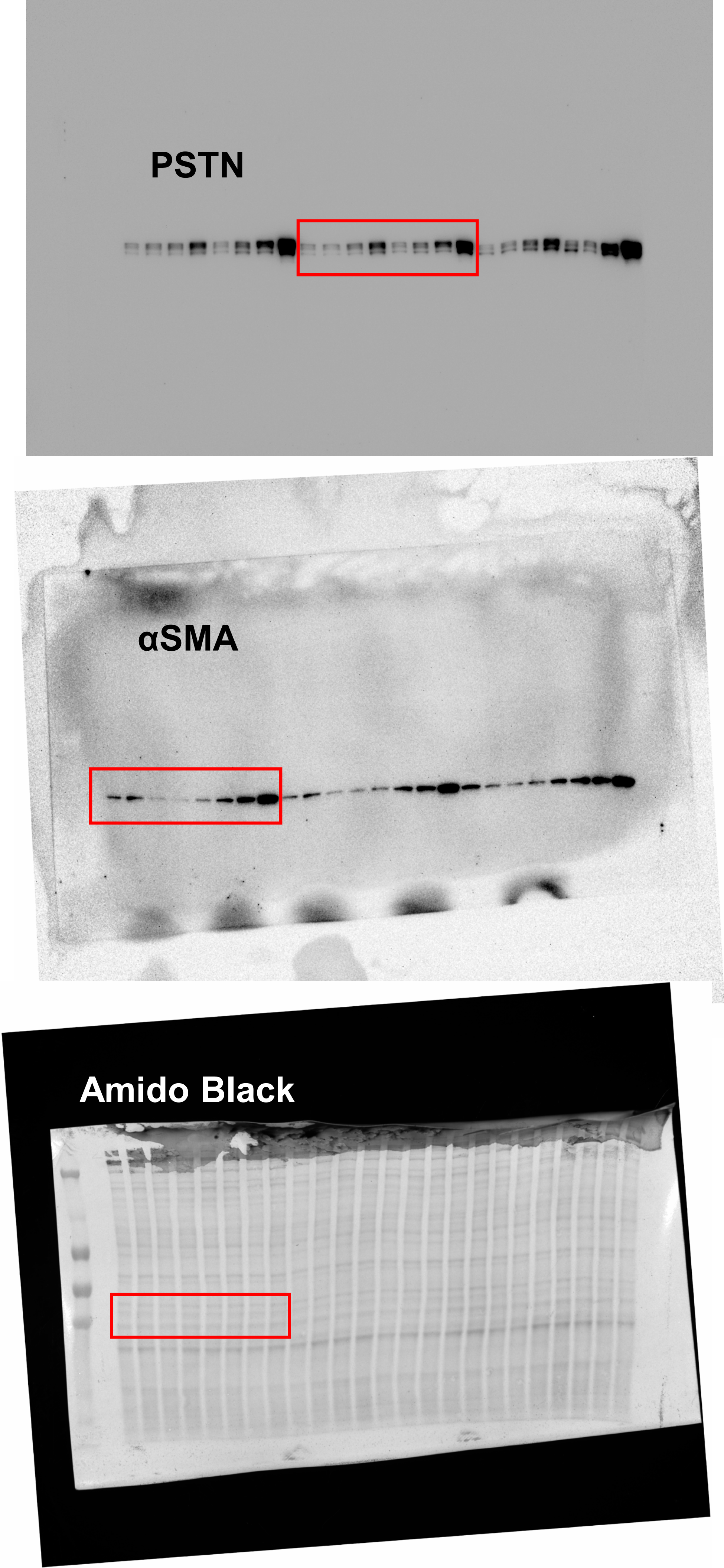
PSTN
αSMA
Amido Black

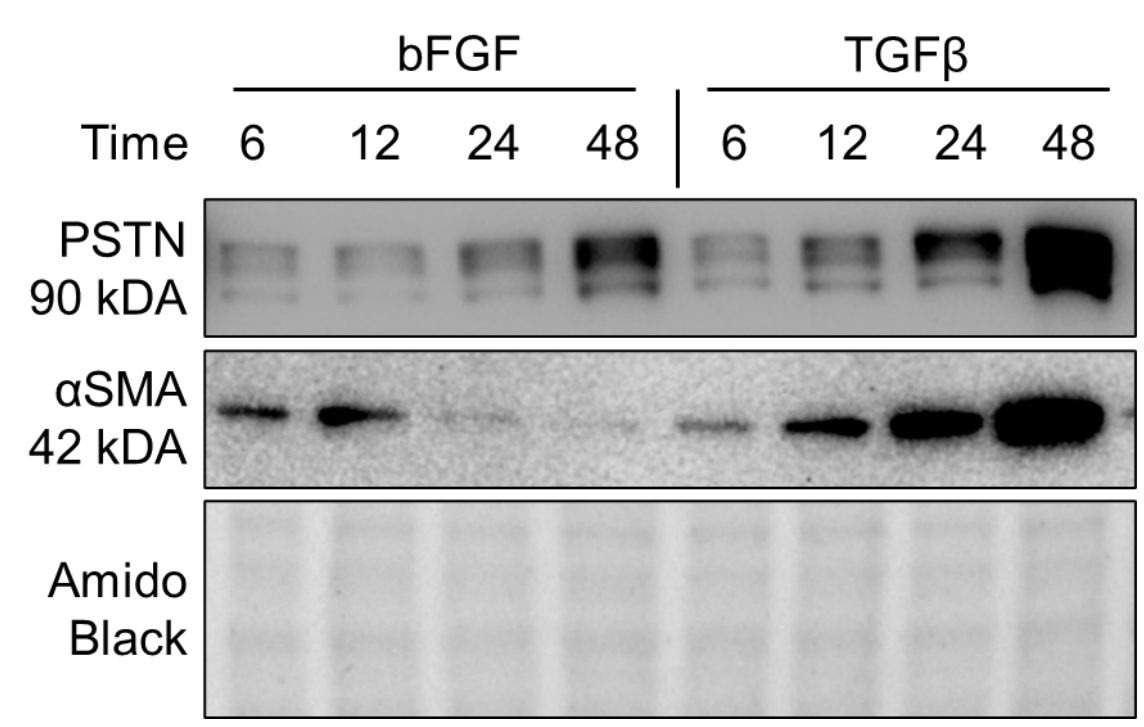
bFGF
TGFβ
Time
6
12
24
48
6
12
24
48
PSTN
90 kDA
αSMA
42 kDA
Amido
Black

Supp 1A

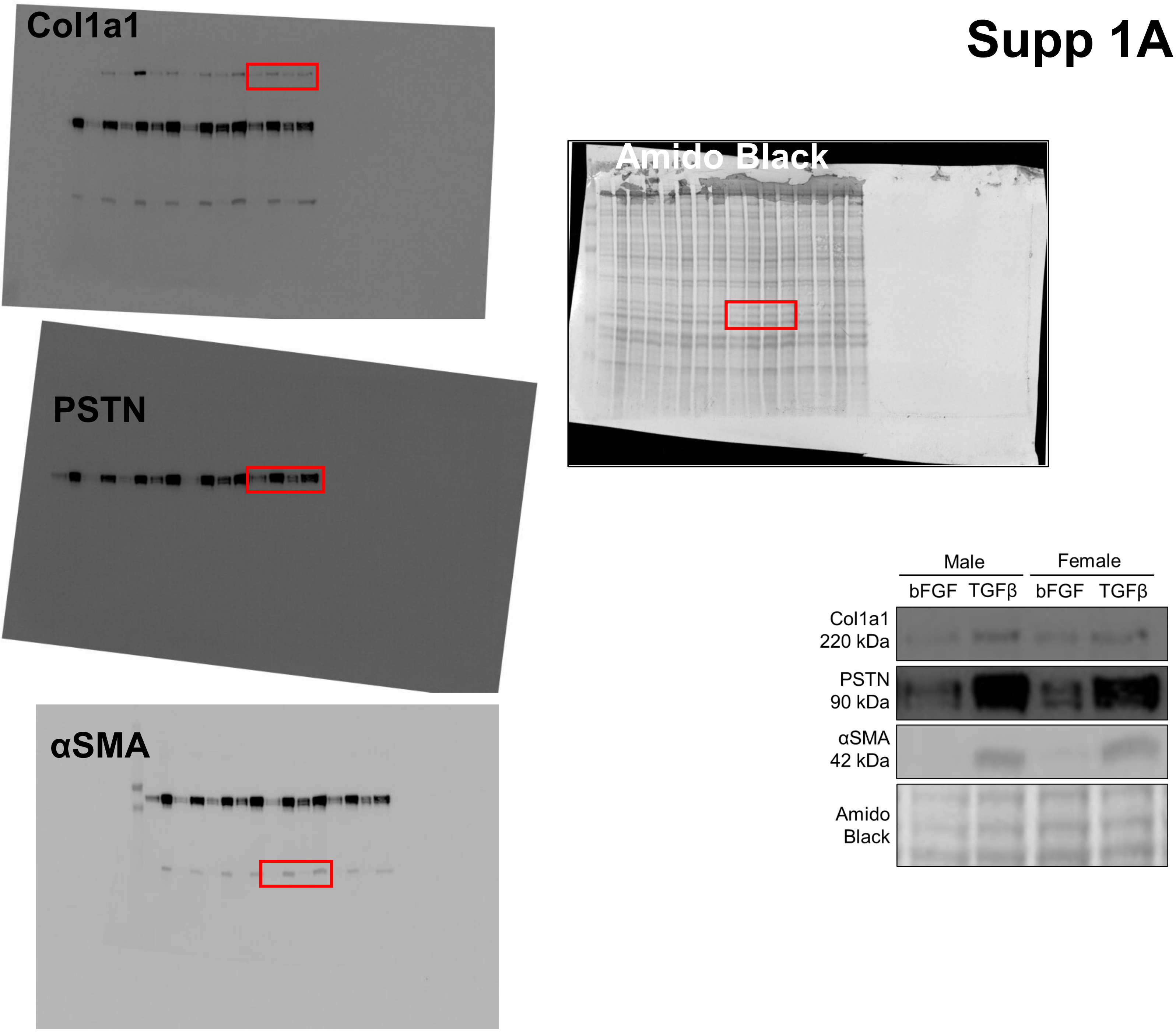

Supp 1B

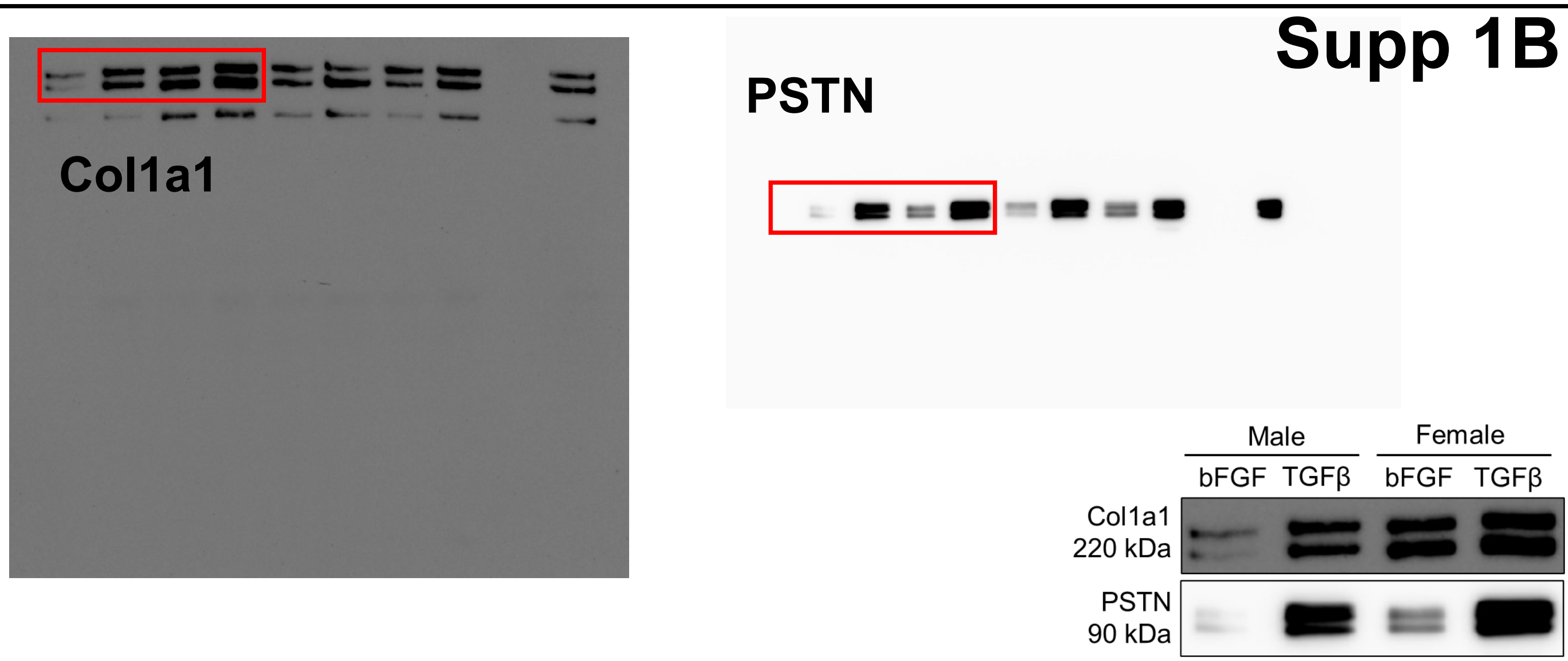

Figure 2D

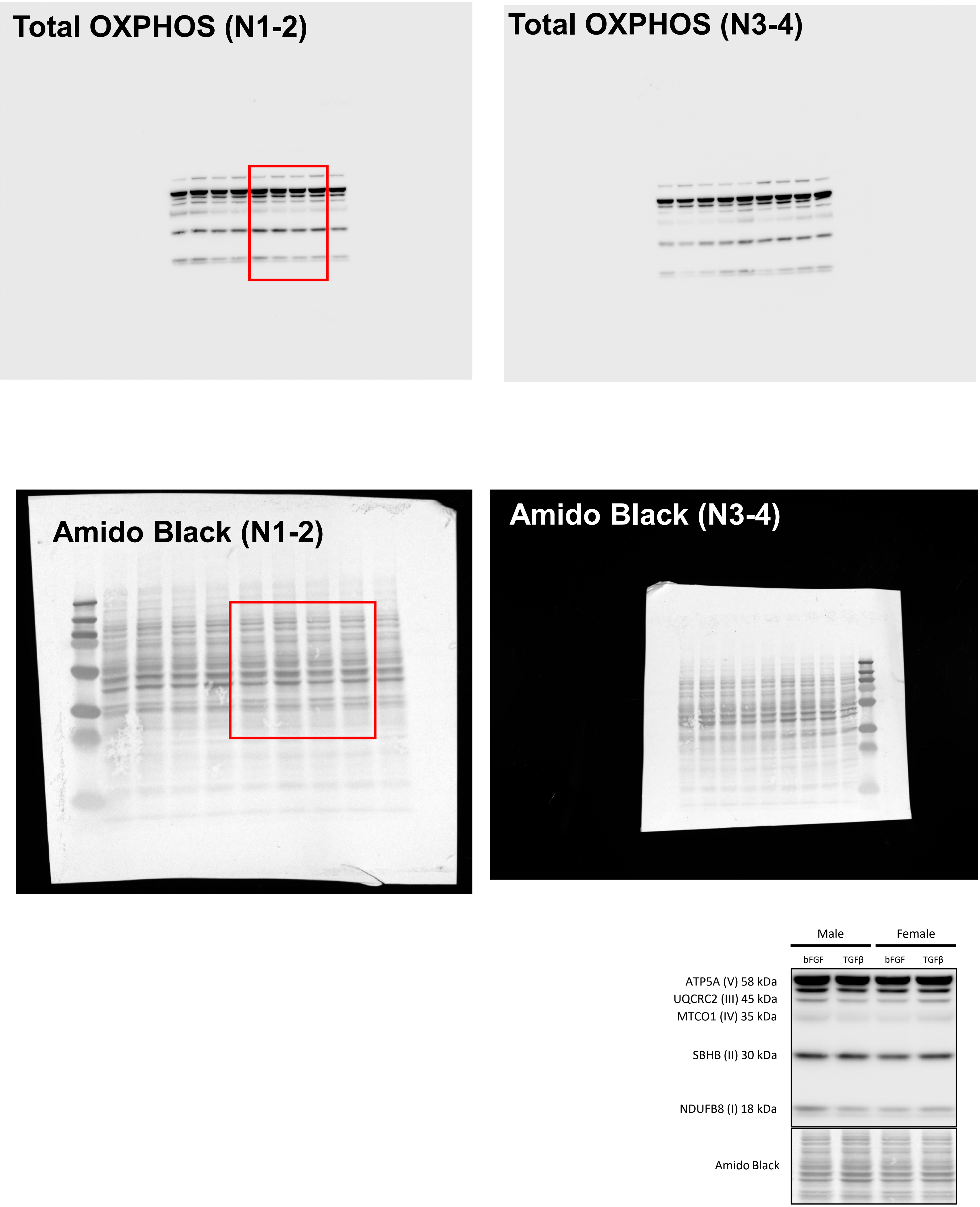
Total OXPHOS (N1-2)
Total OXPHOS (N3-4)
Amido Black (N1-2)
Amido Black (N3-4)
Male
Female
bFGF
TGFβ
bFGF
TGFβ
ATP5A (V) 58 kDa
UQCRC2 (III) 45 kDa
MTCO1 (IV) 35 kDa
SBHB (II) 30 kDa
NDUFB8 (I) 18 kDa
Amido Black

Figure 7C

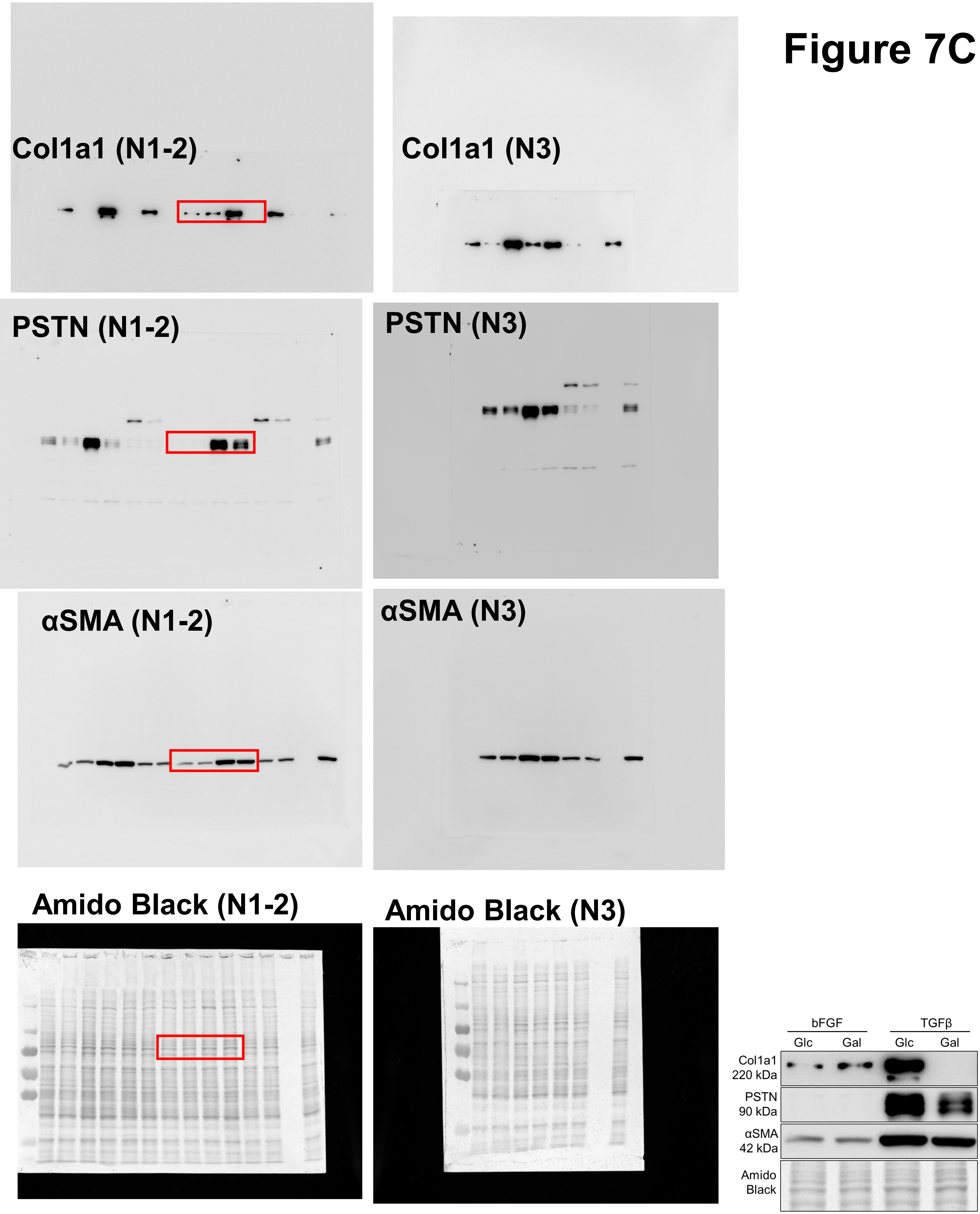

Col1a1 (N1-2)
Col1a1 (N3)
PSTN (N1-2)
PSTN (N3)
αSMA (N1-2)
αSMA (N3)
Amido Black (N1-2)
Amido Black (N3)
bFGF
TGFβ
Glc
Gal
Glc
Gal
Col1a1
220 kDa
PSTN
90 kDa
αSMA
42 kDa
Amido
Black

Figure 7G

Col1a1

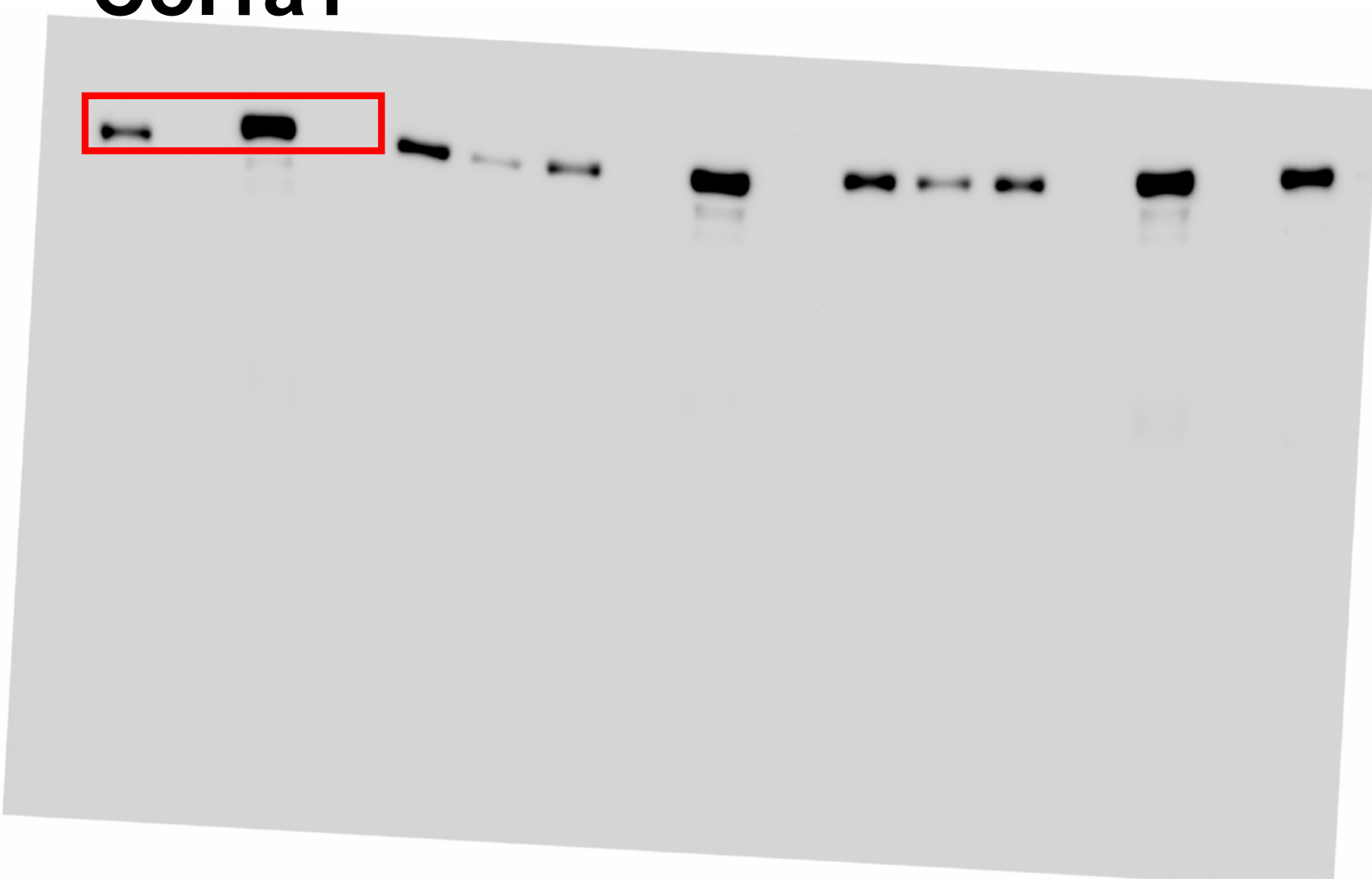

PSTN

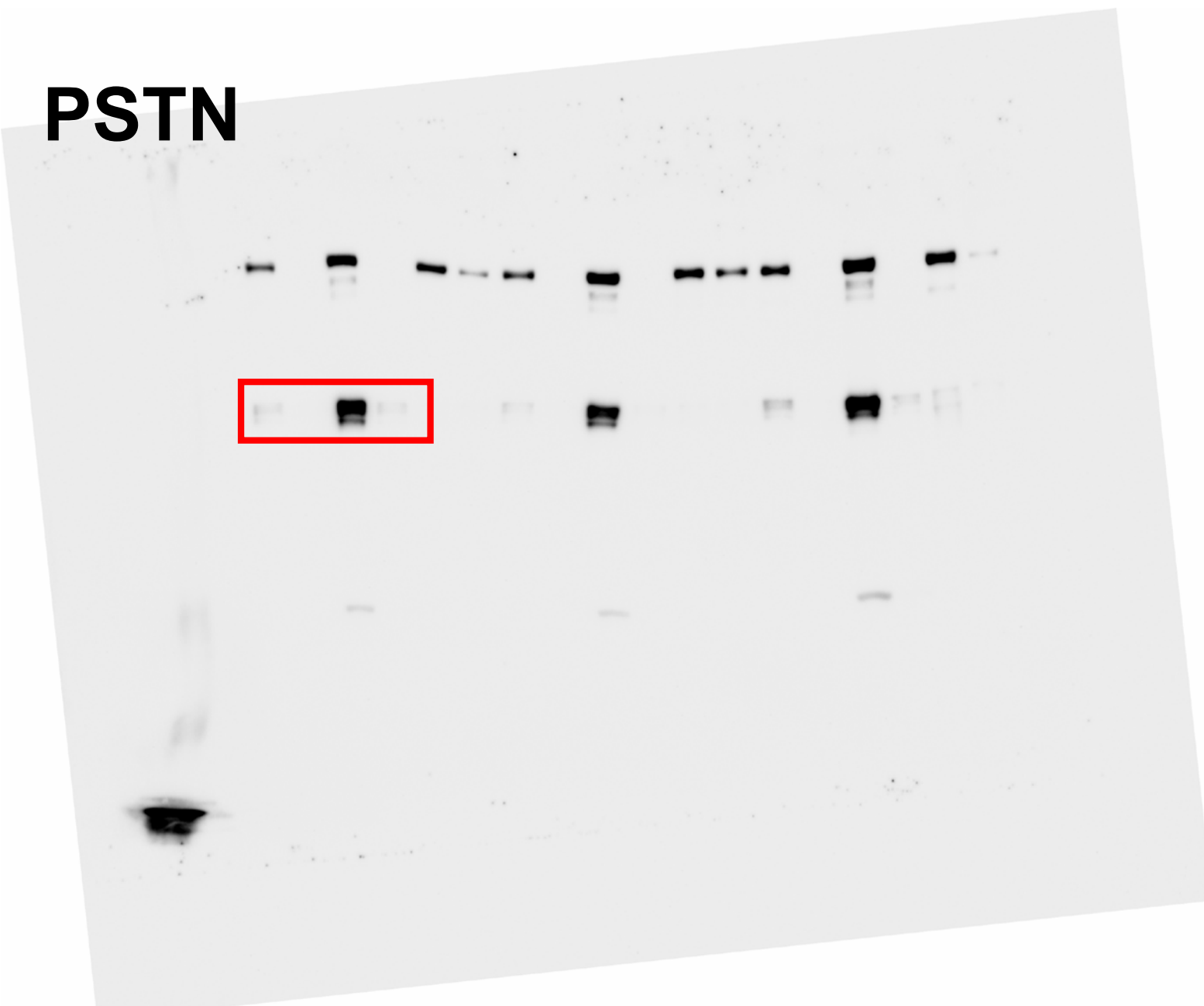

Amido Black (N3)

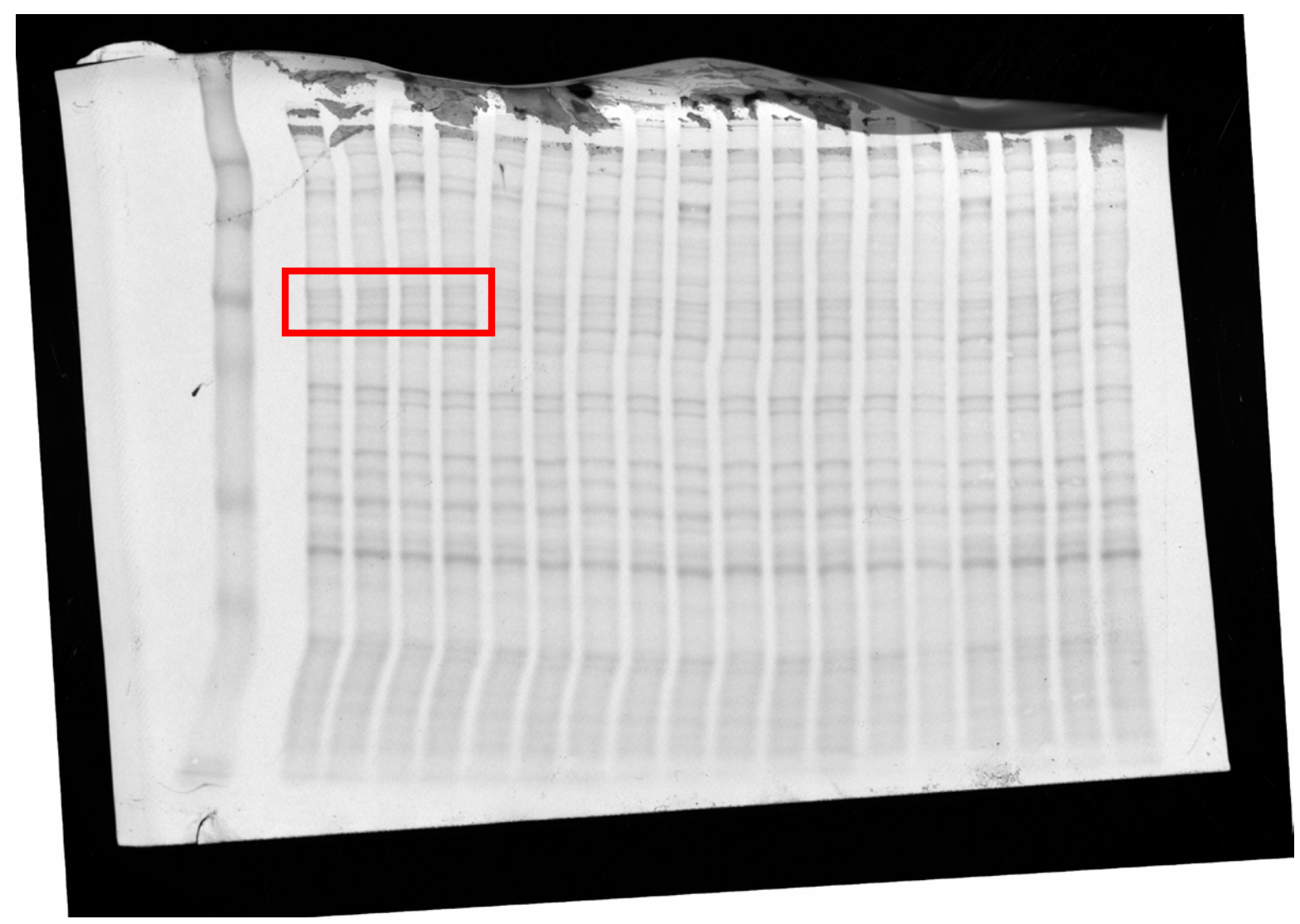

αSMA

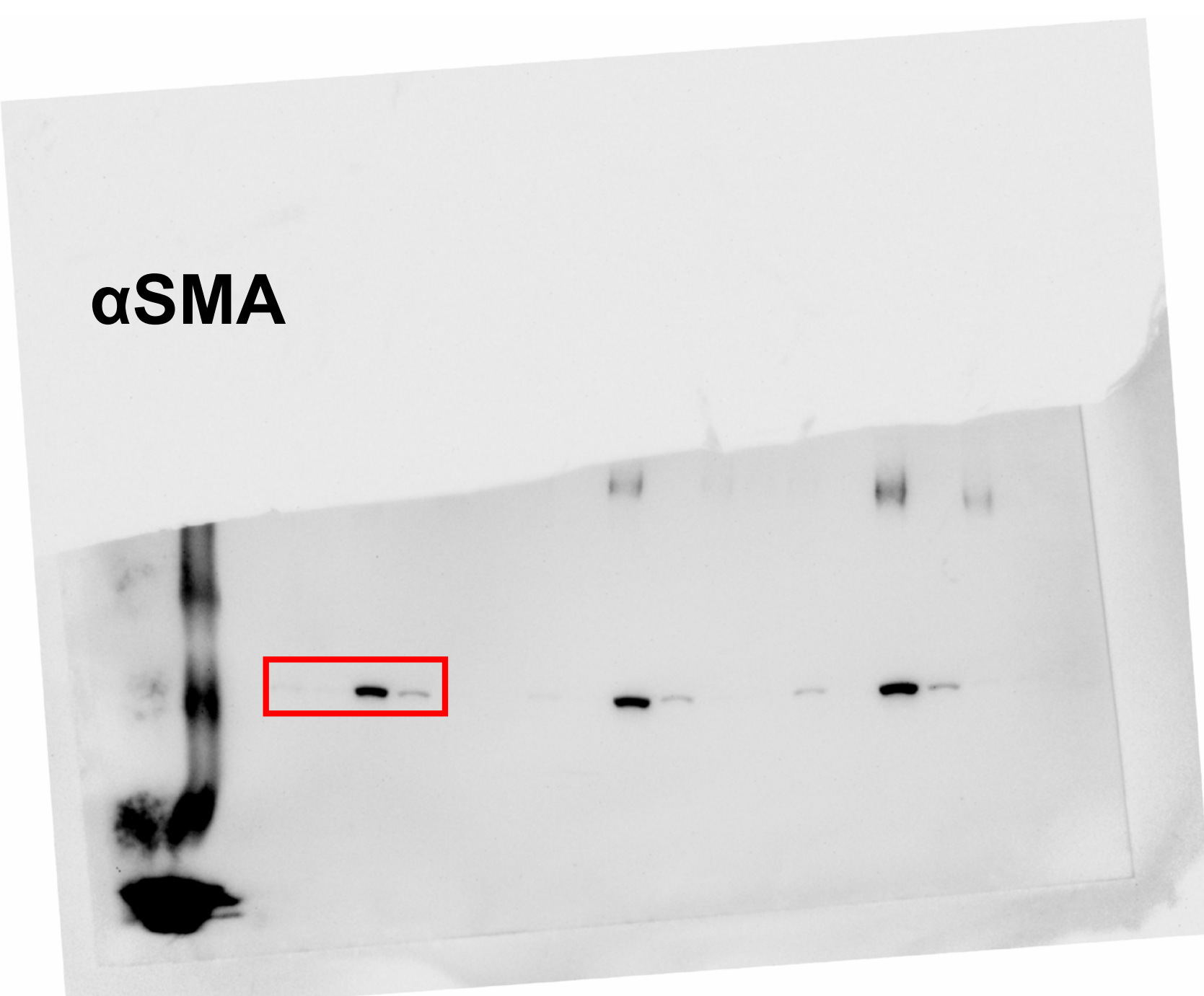

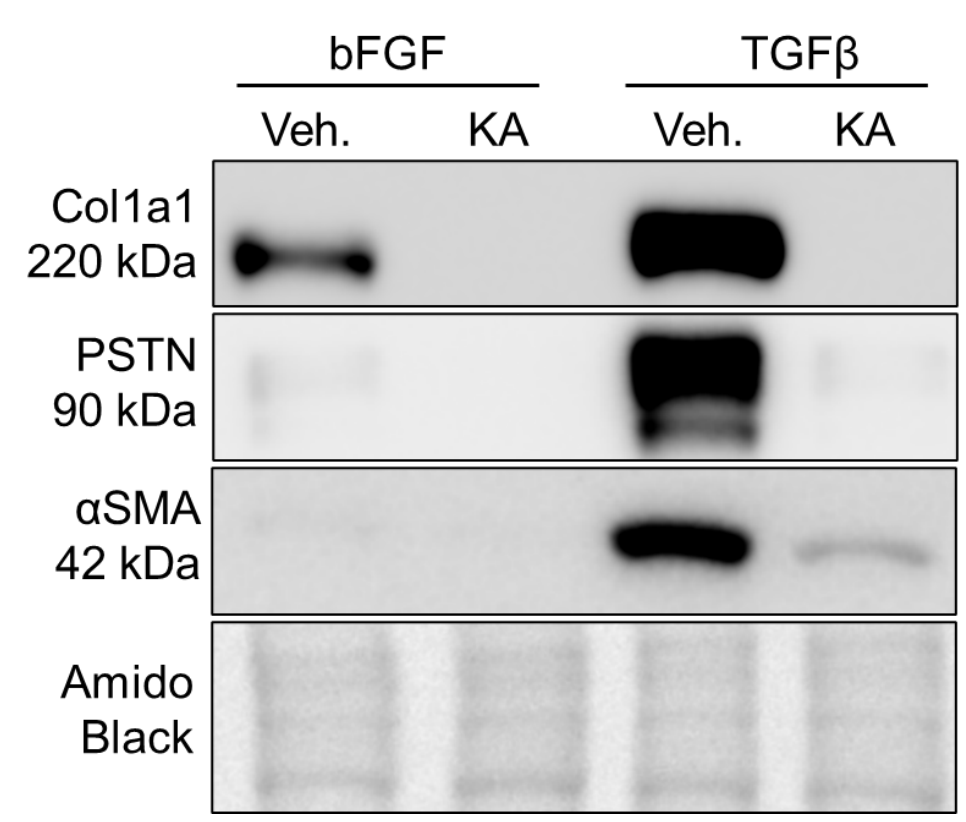

Figure 8A

Col1a1

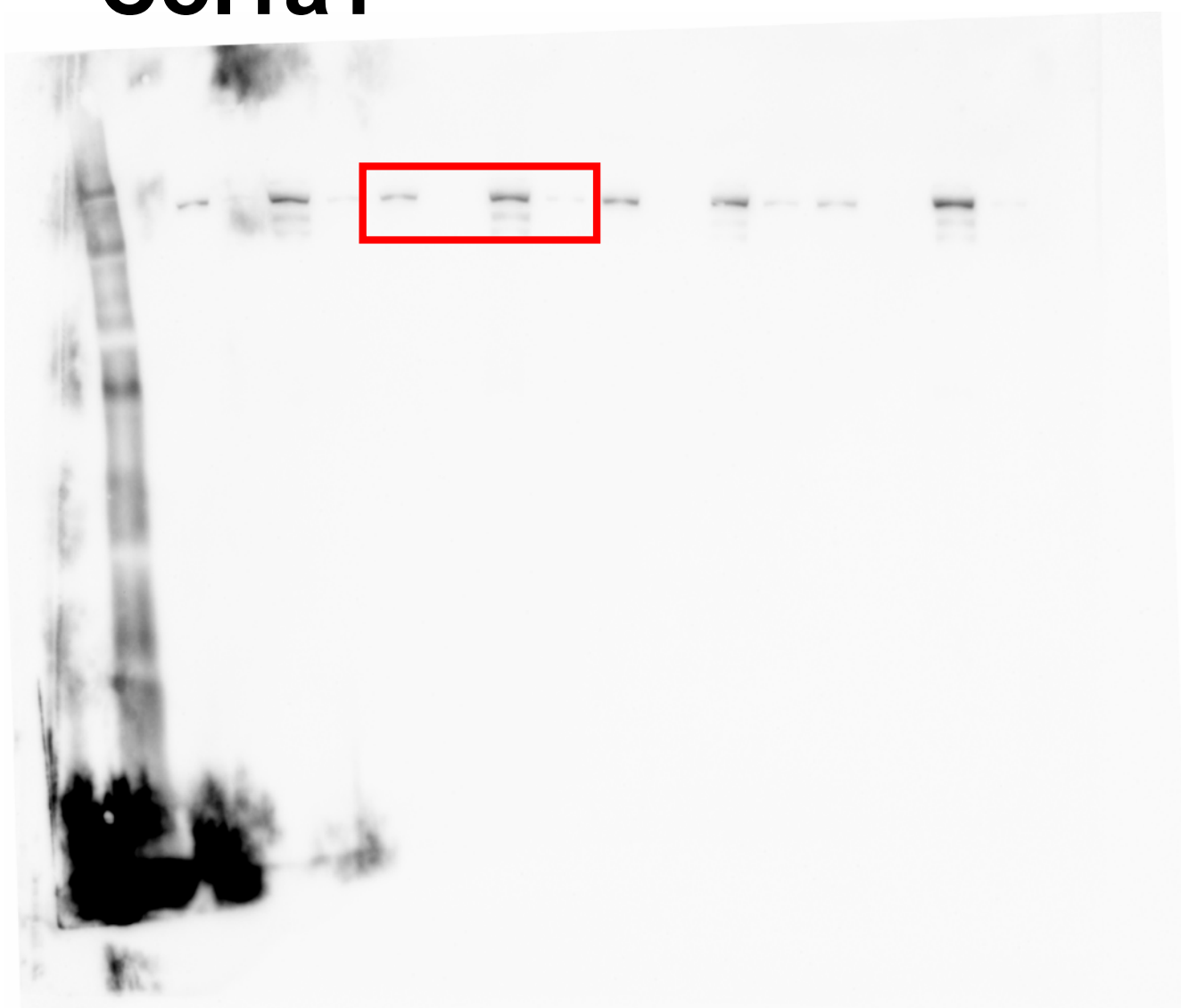

PSTN

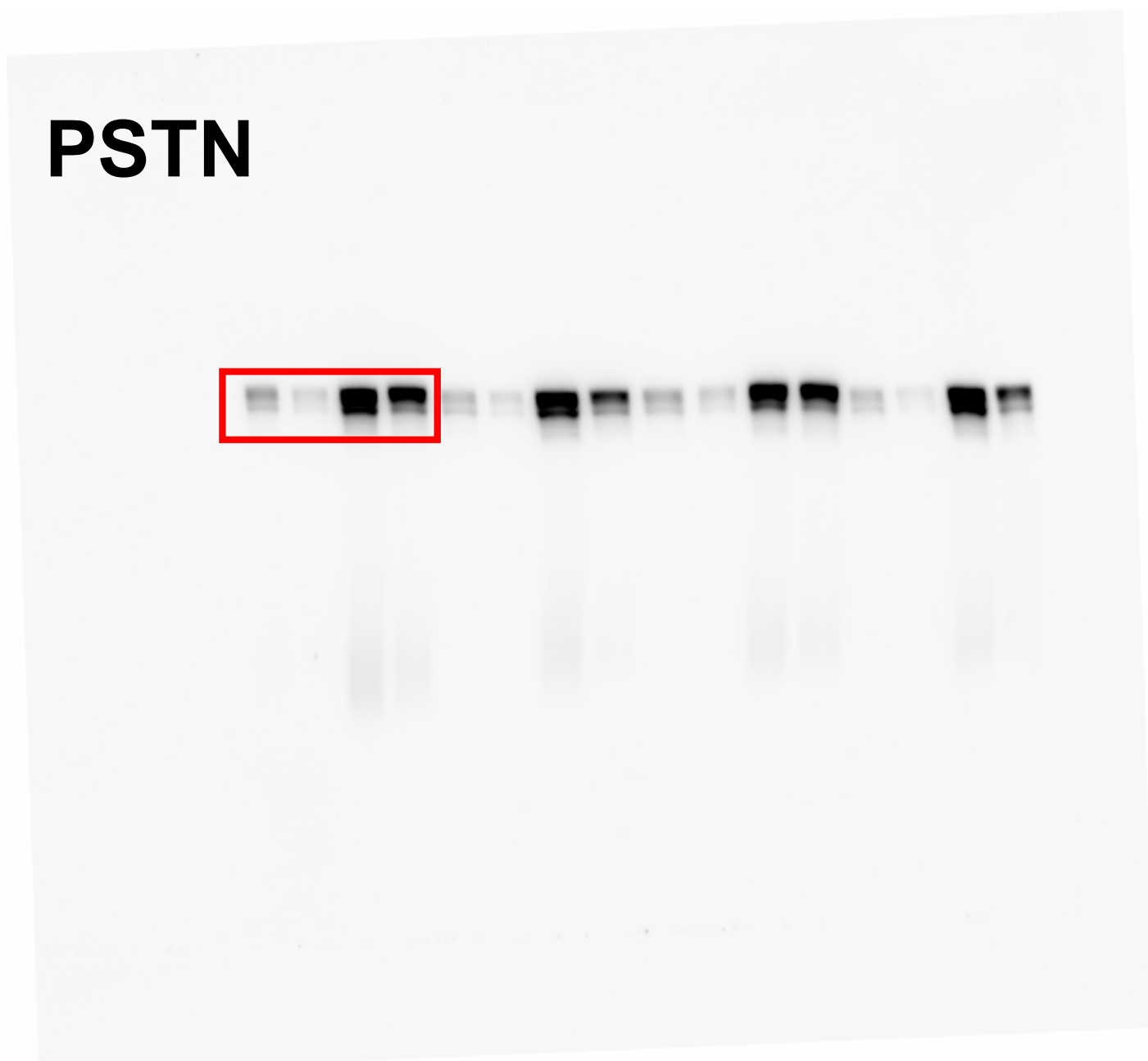

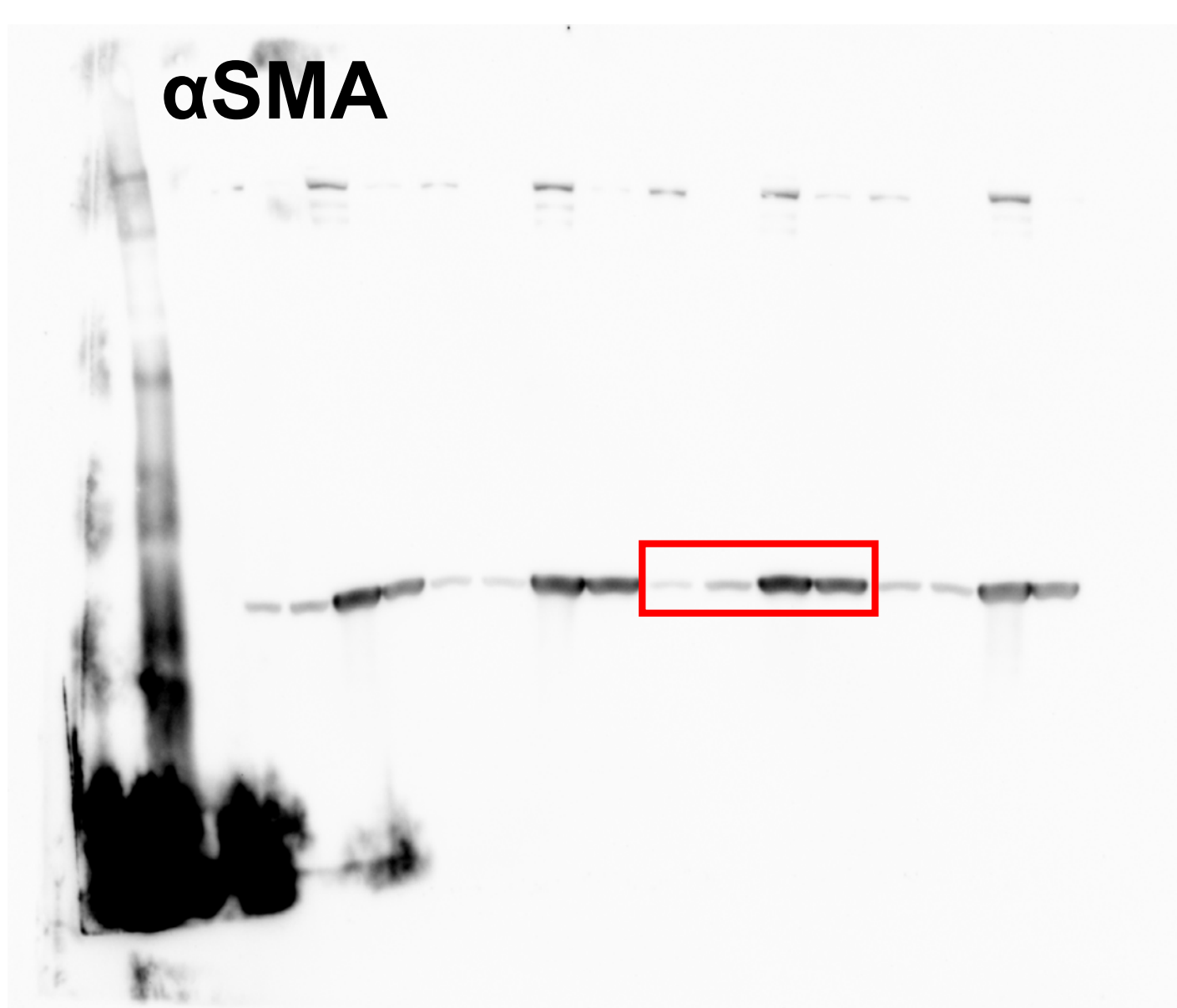

Amido Black (N3)

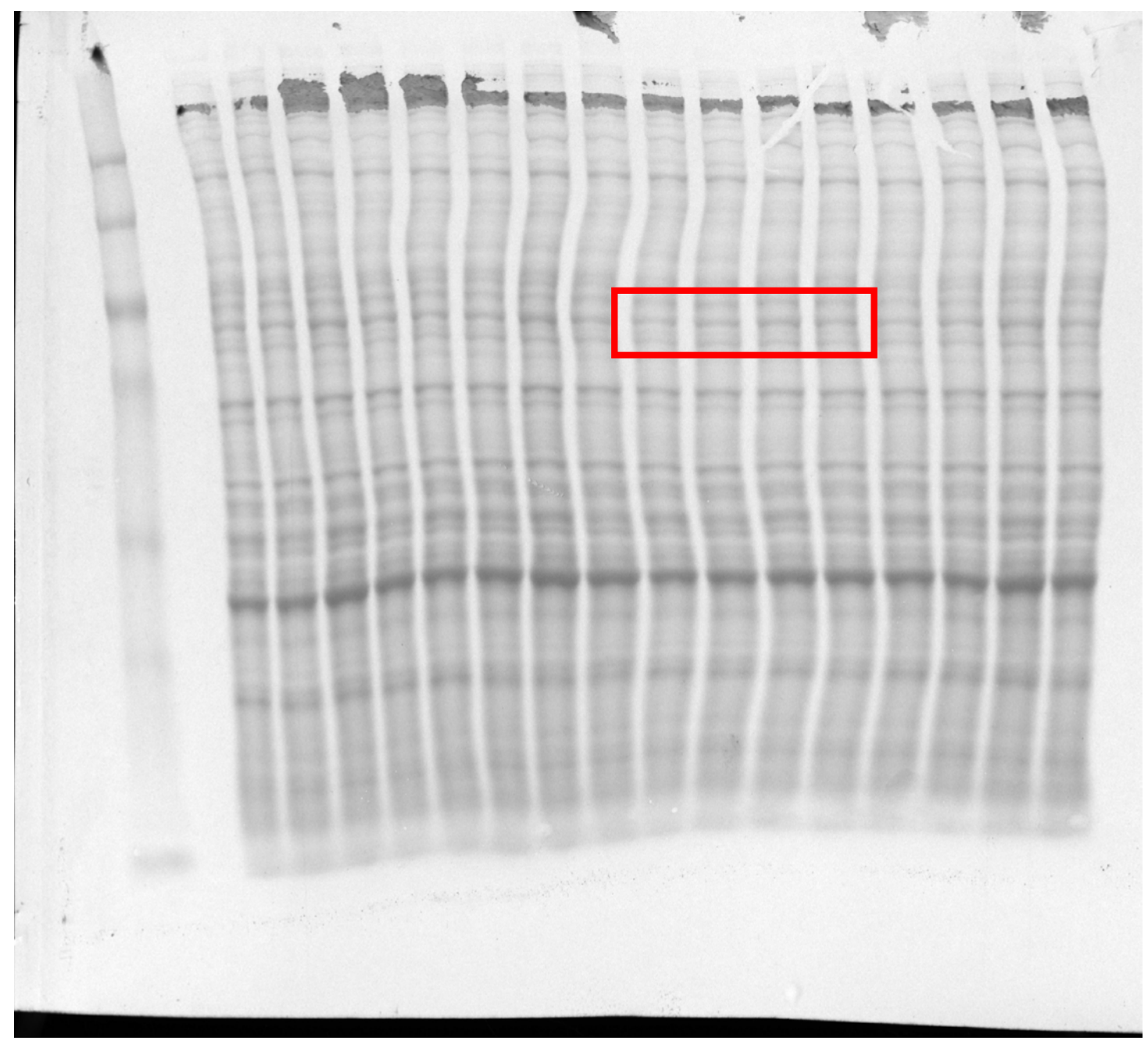

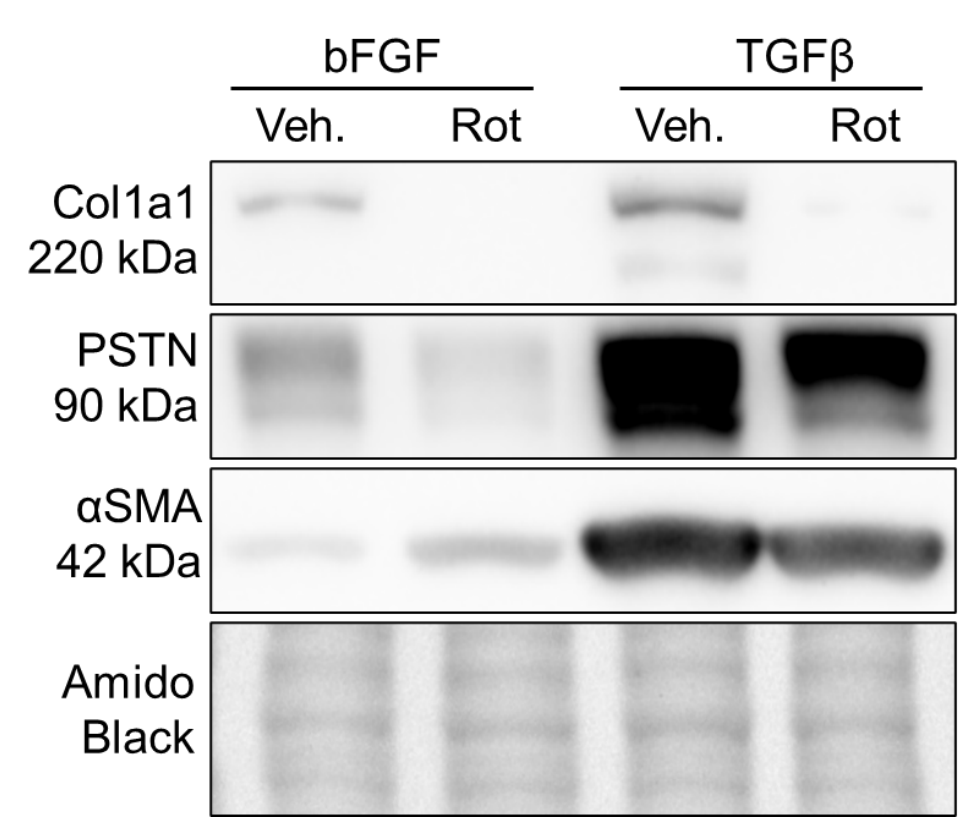

Figure 8C

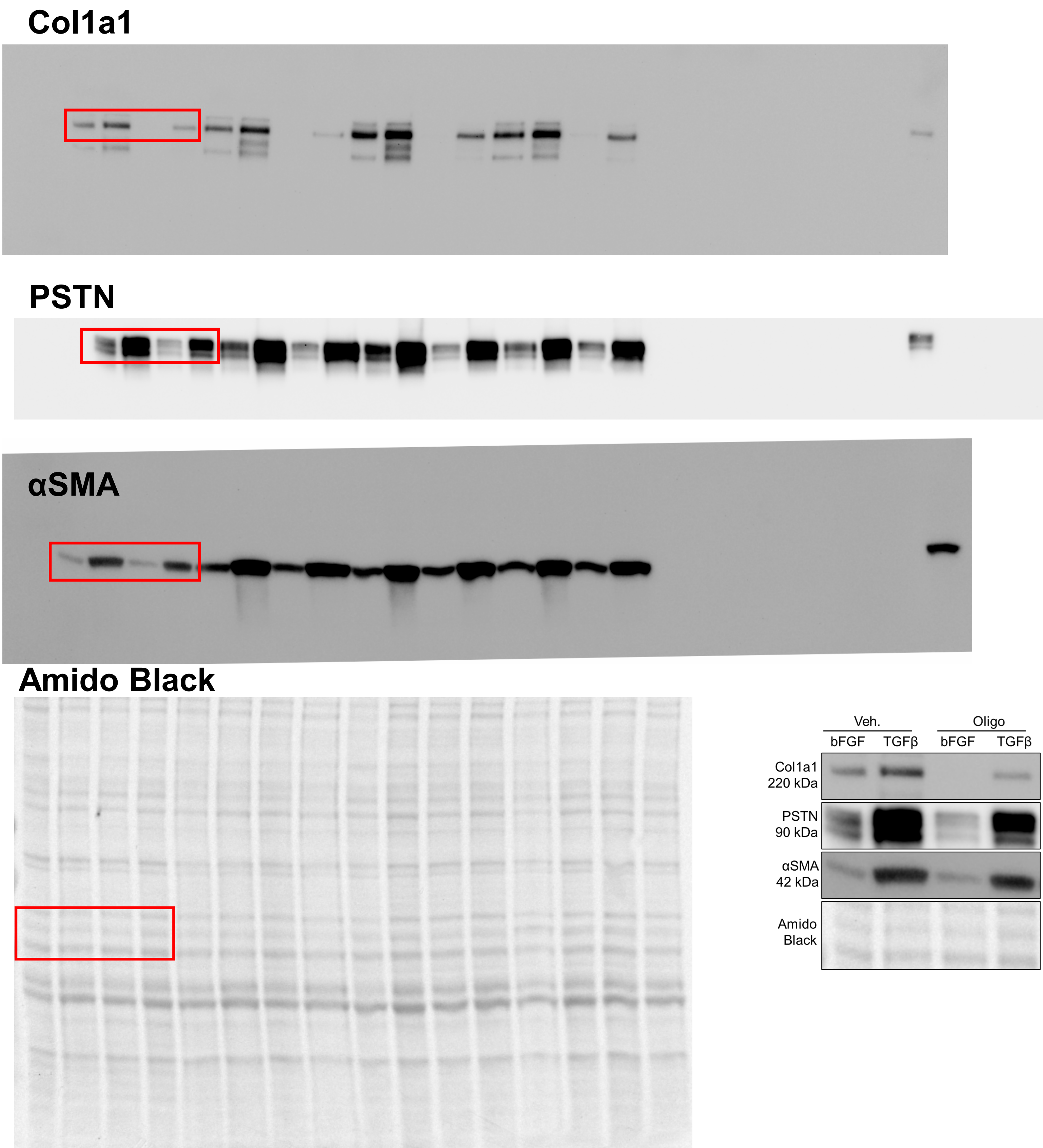
Col1a1
PSTN
αSMA
Amido Black
Veh.
Oligo
bFGF
TGFβ
bFGF
TGFβ
Col1a1
220 kDa
PSTN
90 kDa
αSMA
42 kDa
Amido
Black

Supp 9A

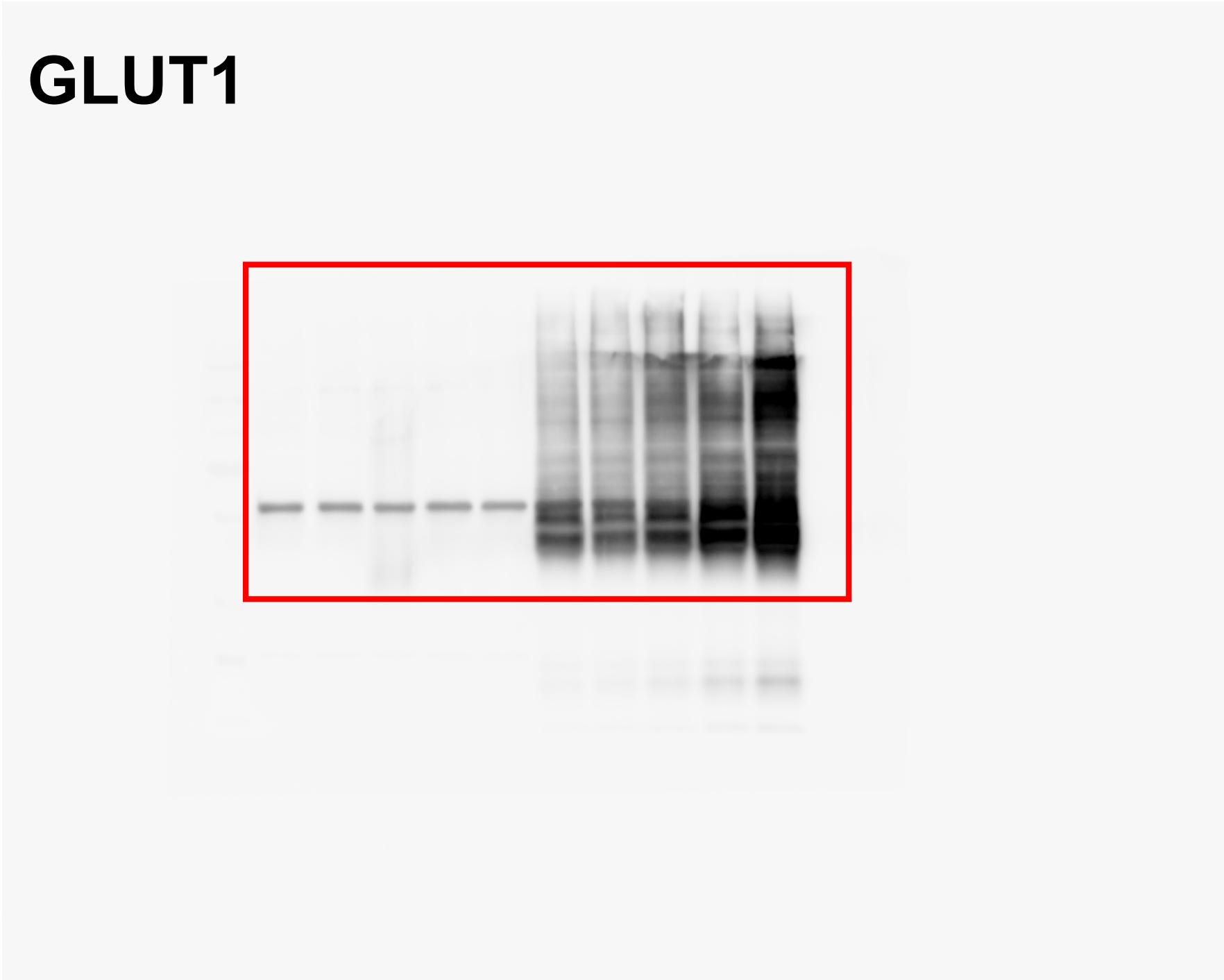
GLUT1

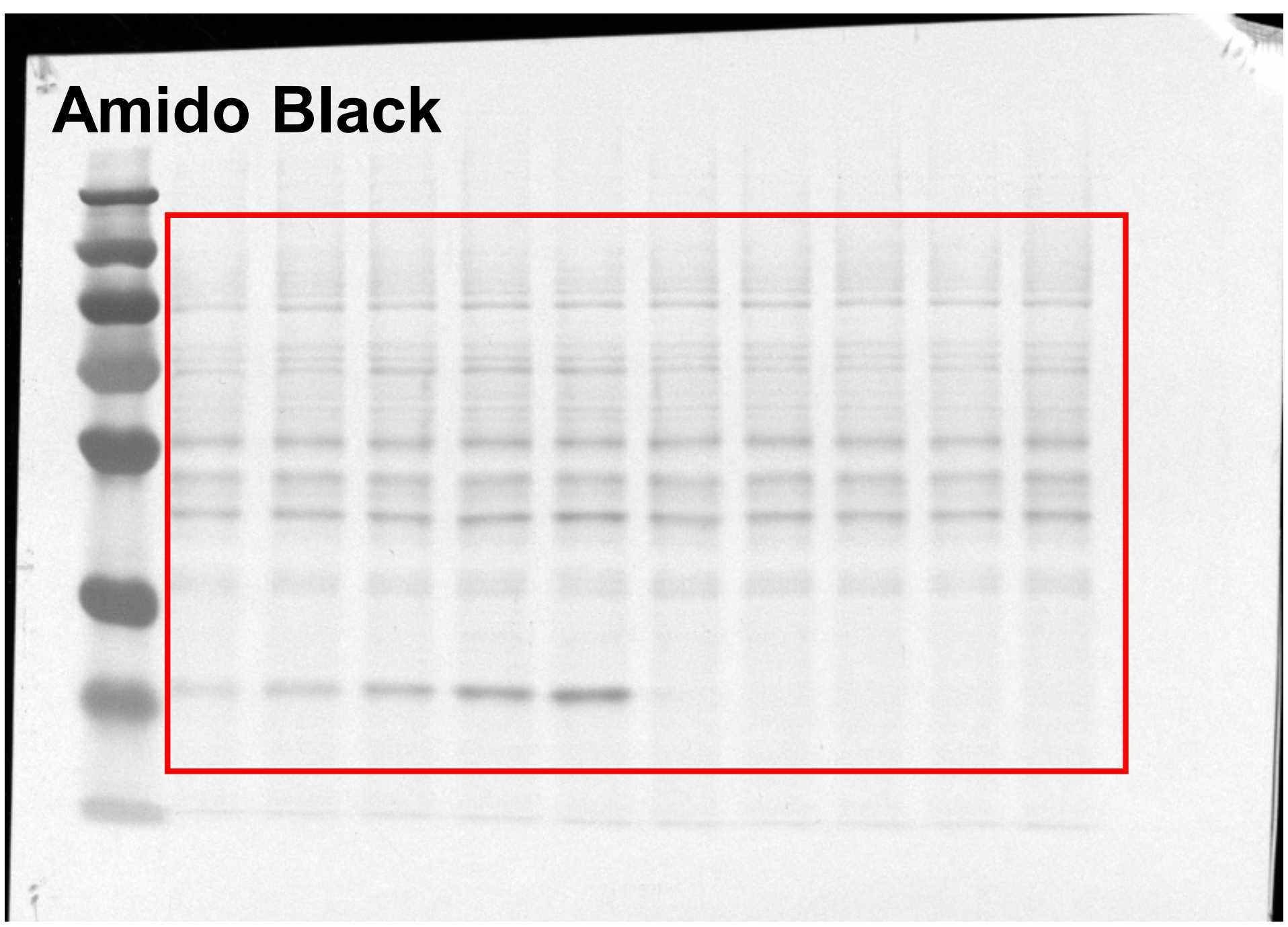
Amido Black

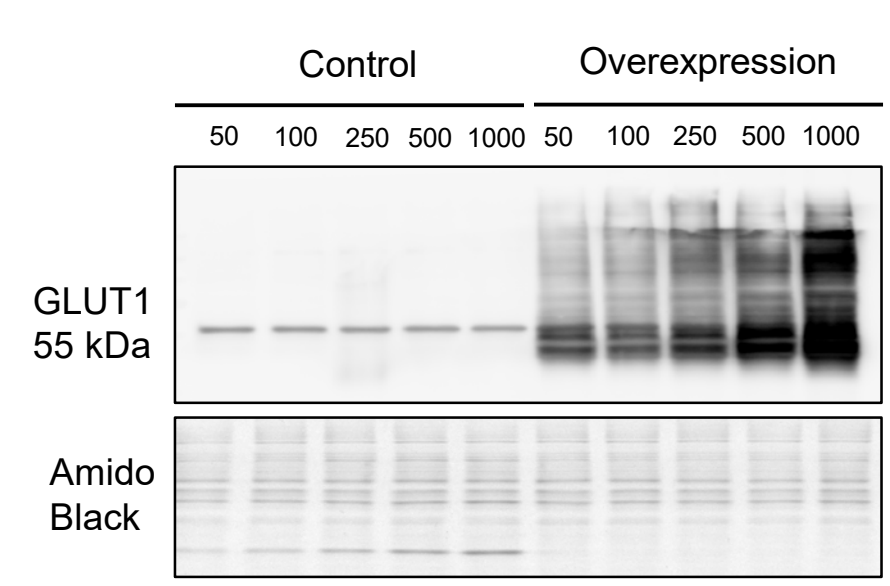
Control
Overexpression
50
100
250
500
1000
50
100
250
500
1000
GLUT1
55 kDa
Amido
Black

Supp 9C

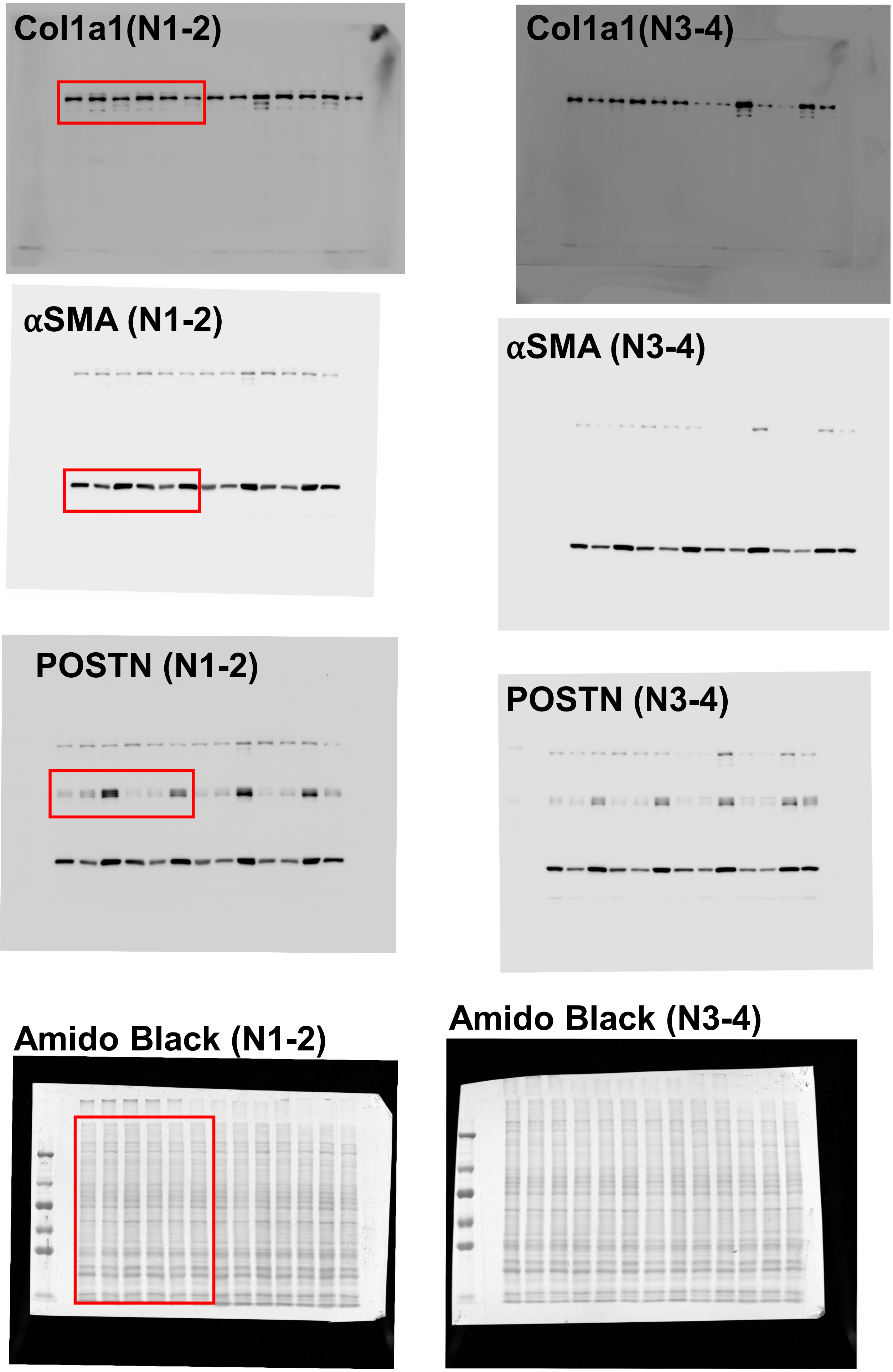
Col1a1(N1-2)
Col1a1(N3-4)
αSMA (N1-2)
αSMA (N3-4)
POSTN (N1-2)
POSTN (N3-4)
Amido Black (N1-2)
Amido Black (N3-4)

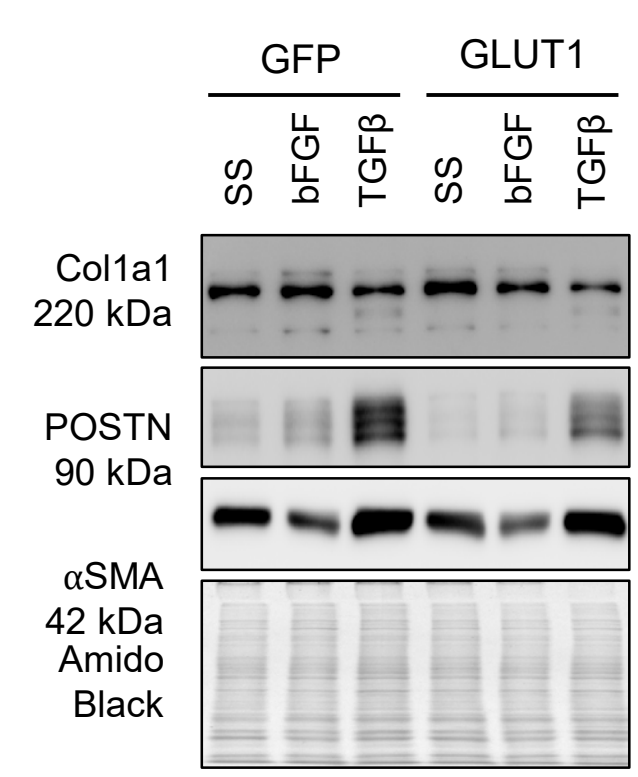
GFP
GLUT1
SS
bFGF
TGFβ
SS
bFGF
TGFβ
Col1a1
220 kDa
POSTN
90 kDa
αSMA
42 kDa
Amido
Black

Supp 9E

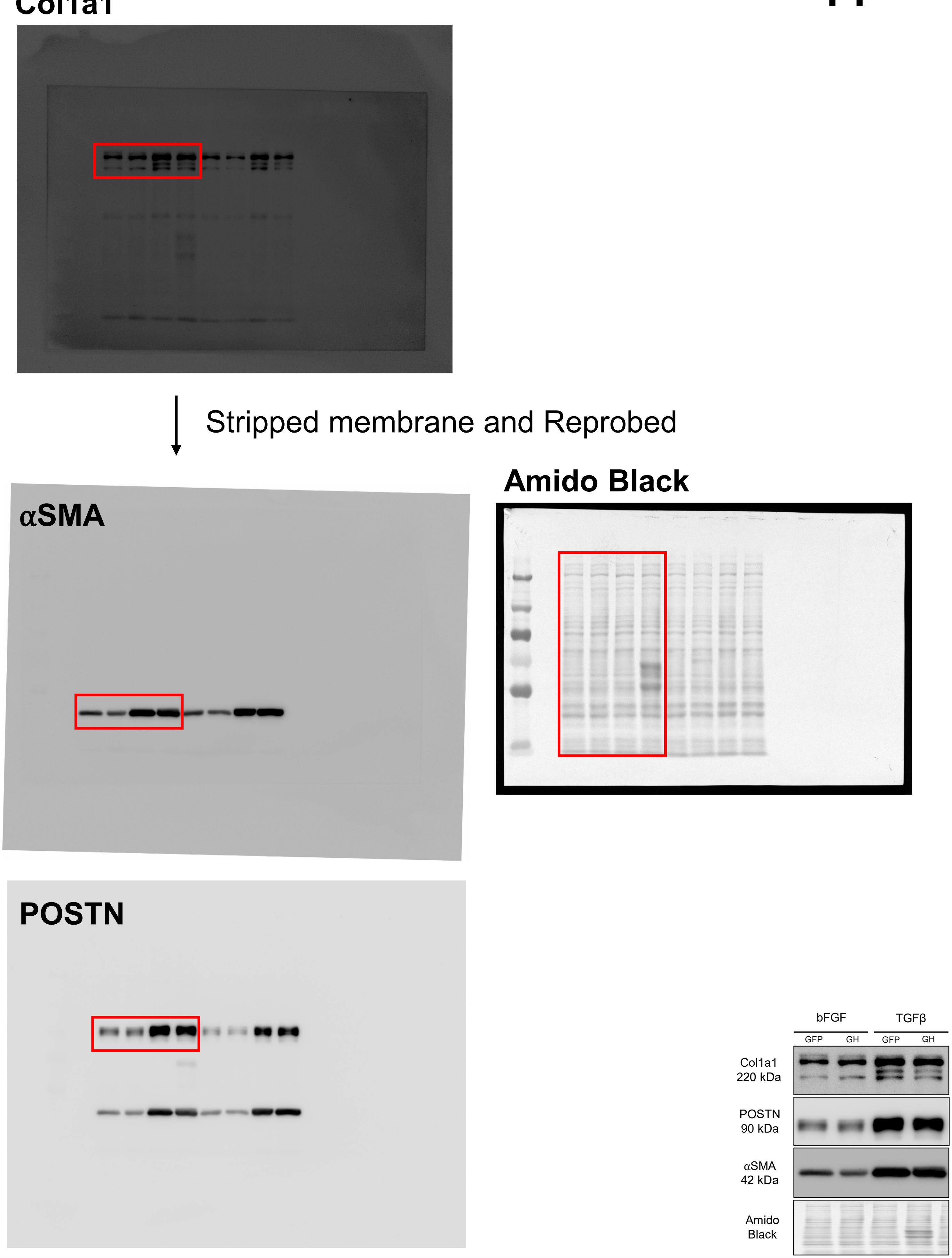
Col1a1
Stripped membrane and Reprobed
αSMA
Amido Black
POSTN
bFGF
TGFβ
GFP
GH
GFP
GH
Col1a1
220 kDa
POSTN
90 kDa
αSMA
42 kDa
Amido
Black
