# Supplementary material for: Phasic metabolic remodeling coordinates cardiac myofibroblast differentiation and function
Source: Redox Biol. 2026 Oct 16;98:104441. doi: 10.1016/j.redox.2026.104441 (PMC13695315; doi:10.1016/j.redox.2026.104441)
Supplement: Supplementary file 2 [file mmc2.pdf]

# Supplementary Figure 1

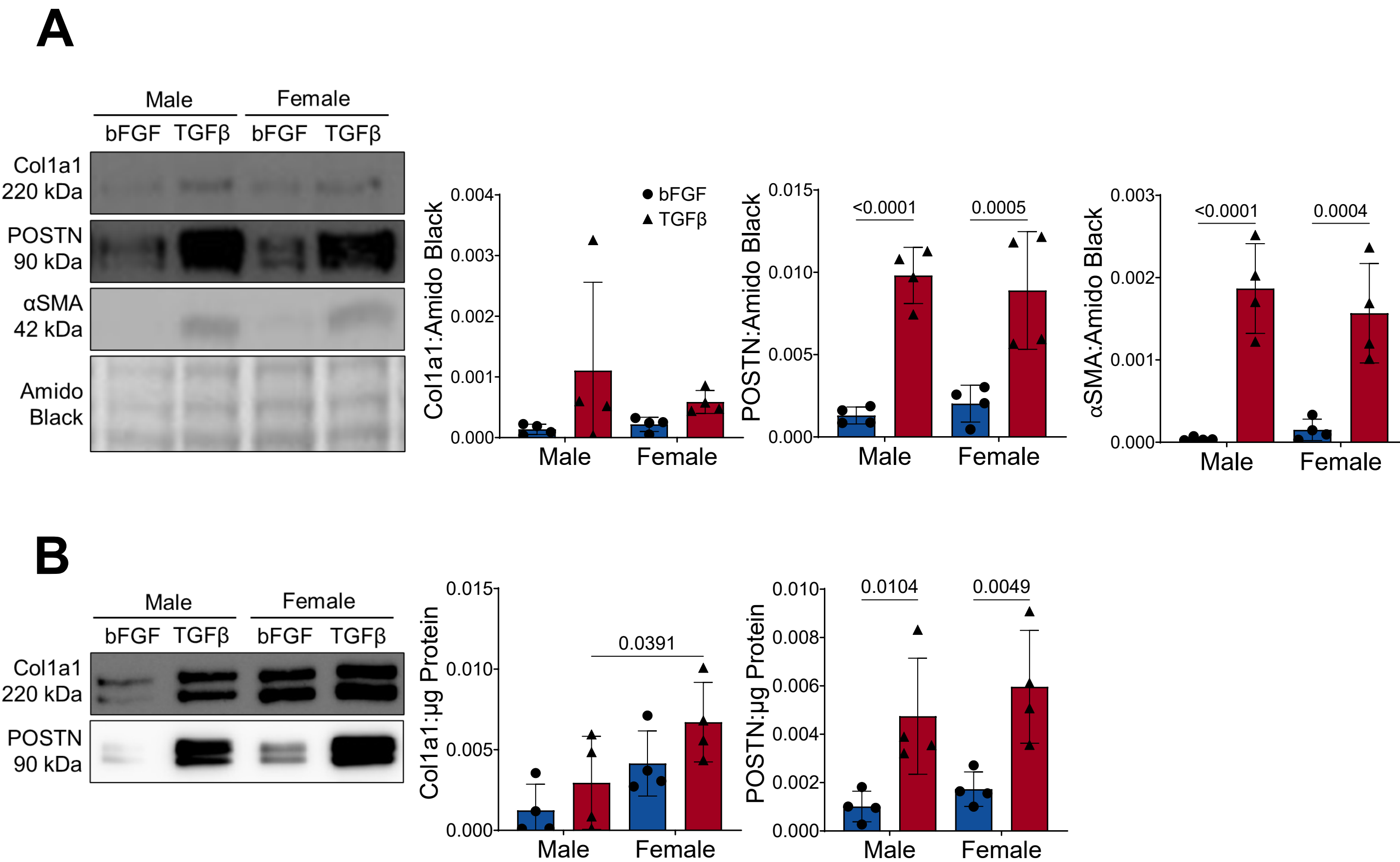


**Supplementary Figure 1. TGFβ induces similar myofibroblast marker responses in male- and female-derived cardiac fibroblasts.** Primary cardiac fibroblasts isolated from adult male and female mice were treated with bFGF (20 ng/mL) or TGFβ (20 ng/mL) for 48 h. **(A)** Representative immunoblots and densitometric quantification of intracellular type I collagen α1 chain (COL1A1), periostin (POSTN), and α-smooth muscle actin (αSMA). **(B)** Representative immunoblots and quantification of COL1A1 and POSTN in conditioned medium. Intracellular protein abundance was normalized to total lane protein visualized by amido black staining. Conditioned medium signals were normalized to cell protein. Data are mean ± SD from four independent fibroblast isolations per sex, each obtained from a different mouse. Statistical comparisons were performed by two-way ANOVA followed by Šídák's multiple-comparisons test.

# Supplementary Figure 2

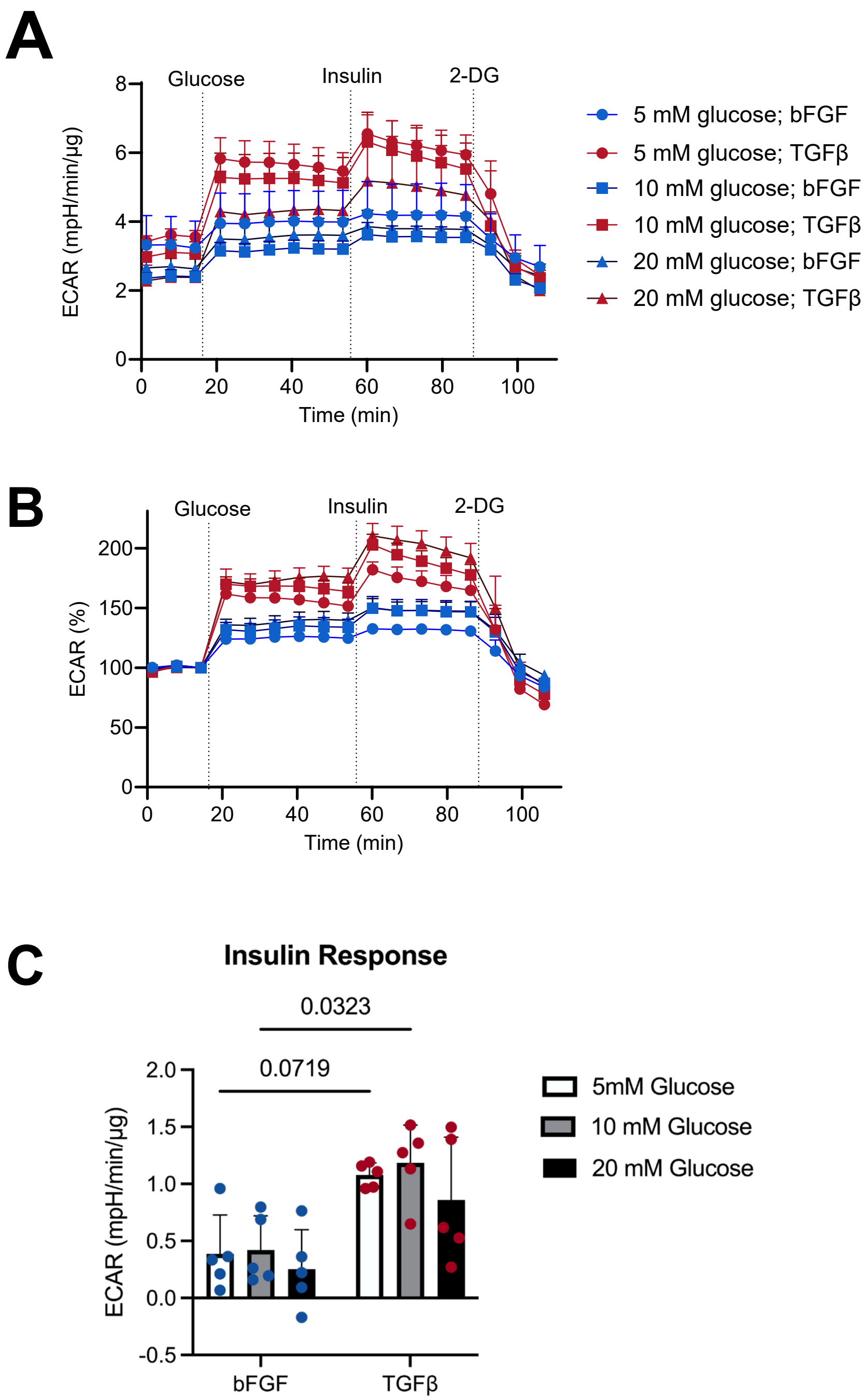


**Supplementary Figure 2. TGFβ enhances insulin-stimulated glycolytic activity in cardiac fibroblasts.** Primary cardiac fibroblasts isolated from adult male mice were treated with bFGF (20 ng/mL) or TGFβ (20 ng/mL) for 48 h before extracellular flux analysis. **(A)** Protein-normalized extracellular acidification rate (ECAR) following addition of glucose at the indicated final concentrations, insulin (100 nM), and 2-deoxy-D-glucose (2-DG; 50 mM). **(B)** Baseline-subtracted ECAR traces from the experiment shown in A. **(C)** Insulin-stimulated glycolytic response, calculated as the increase in ECAR following insulin addition relative to the pre-insulin rate. Data are mean ± SD from five independent fibroblast isolations, each obtained from a different male mouse. Statistical comparisons in C were performed by two-way ANOVA followed by Šídák's multiple-comparisons test. Data are shown as means ± SD.

# Supplementary Figure 3

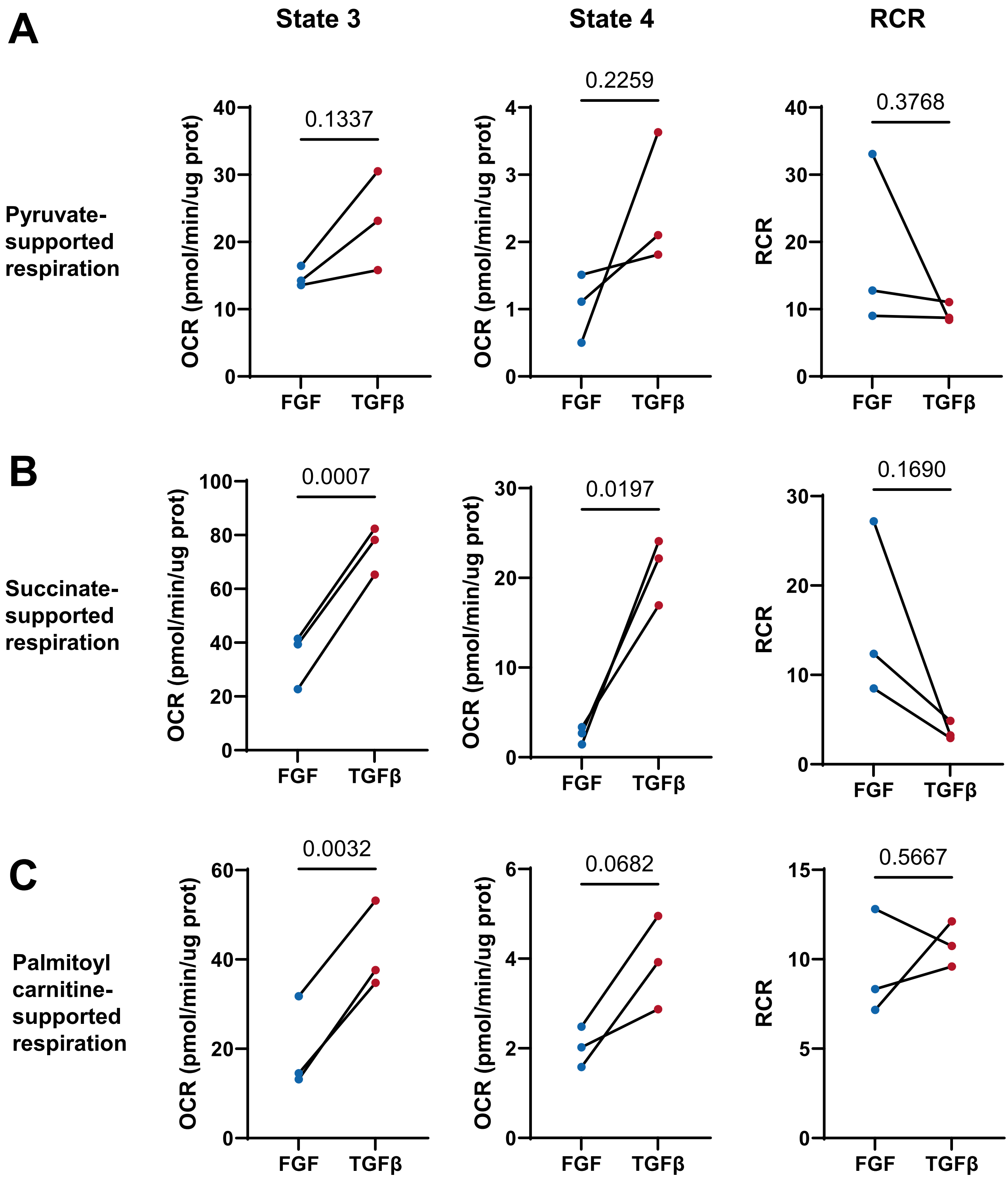


**Supplementary Figure 3. TGFβ increases substrate-supported respiration in permeabilized cardiac fibroblasts.** Primary cardiac fibroblasts were treated with bFGF (20 ng/mL) or TGFβ (20 ng/mL) for 48 h and permeabilized with saponin (25 µg/mL). Oxygen consumption was measured in the presence of **(A)** pyruvate (5 mM), malate (2.5 mM), and ADP (1 mM); **(B)** succinate (5 mM), rotenone (1 µM), and ADP (1 mM); or **(C)** palmitoylcarnitine (50 µM), malate (2.5 mM), and ADP (1 mM). ADP-stimulated respiration was designated state 3 respiration. Oligomycin (1 µM) was subsequently added to determine state $4_o$ respiration, and the respiratory control ratio was calculated as state 3 divided by state $4_o$ respiration. Data are mean ± SD from three paired independent fibroblast isolations, each obtained from a different mouse. Differences between bFGF- and TGFβ-treated cells were assessed by paired Student's *t*-test.

# Supplementary Figure 4

## GO Biological Processes
## TGFβ/bFGF

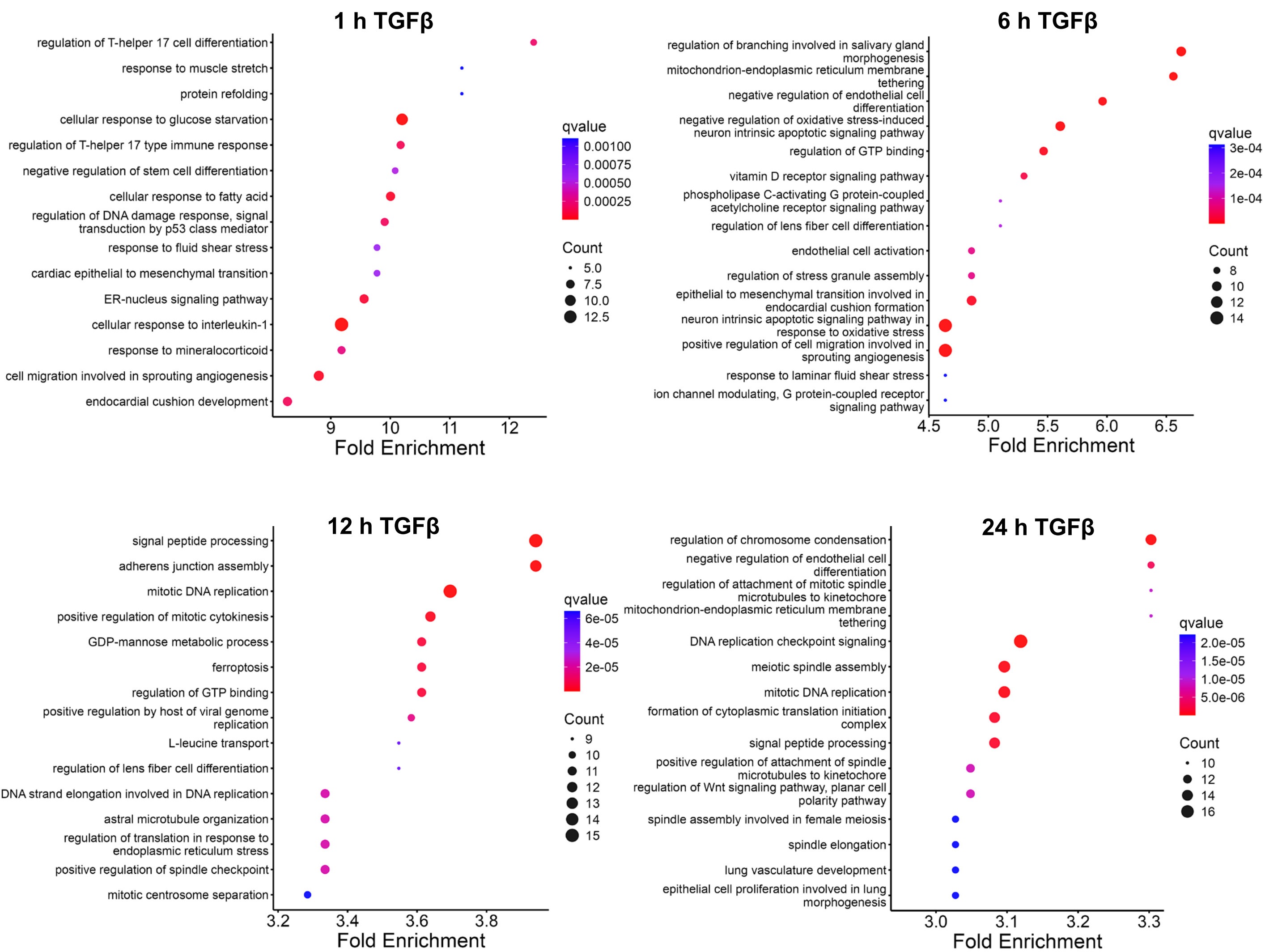


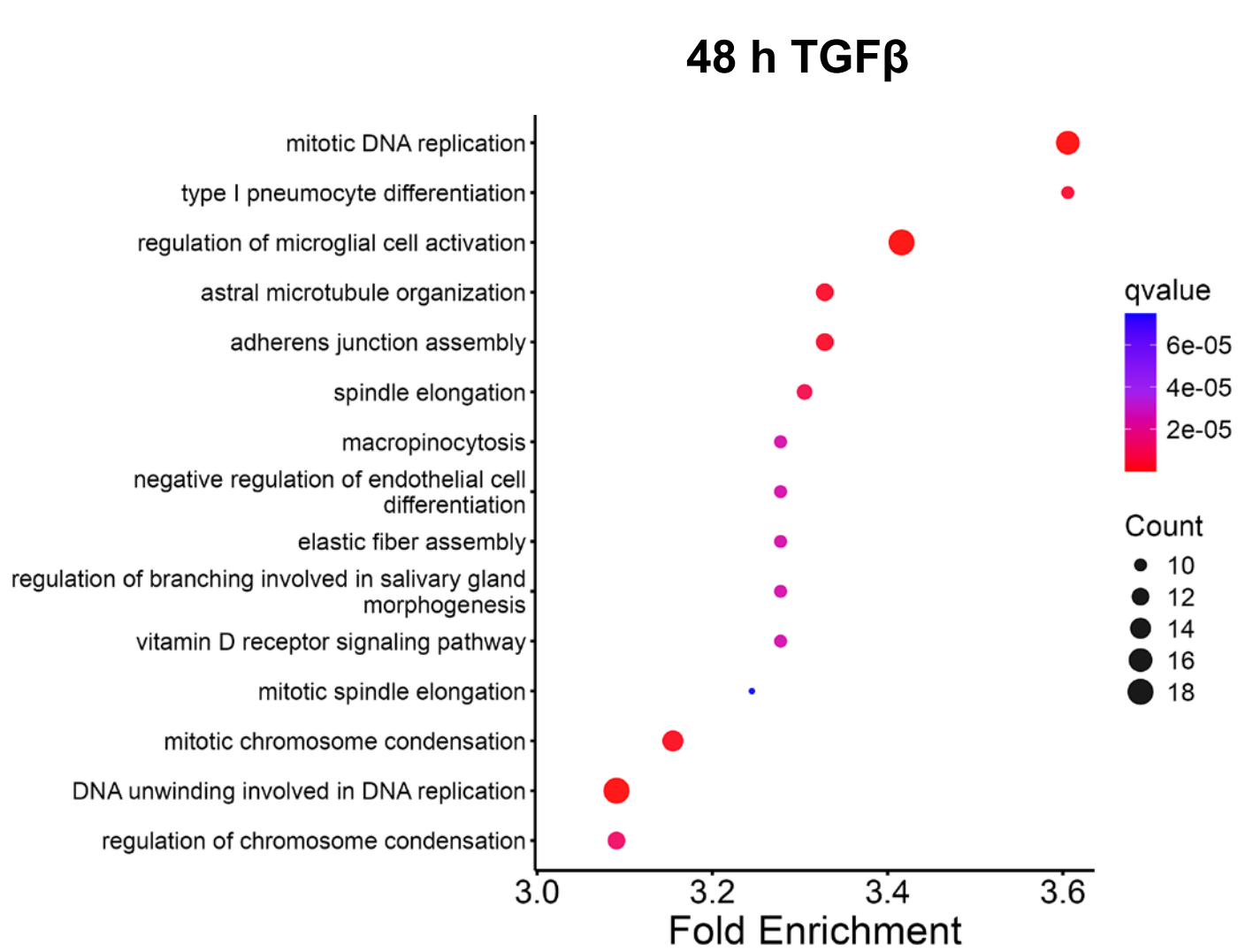


**Supplementary Figure 4. Gene Ontology analysis reveals time-dependent transcriptional remodeling after TGFβ stimulation.** Primary cardiac fibroblasts were treated with TGFβ or bFGF for 1, 6, 12, 24, or 48 h before bulk RNA sequencing. Bubble plots show the 15 most highly enriched Gene Ontology Biological Process terms among differentially expressed genes identified at each time point in TGFβ-treated fibroblasts relative to bFGF-treated fibroblasts. The x-axis indicates fold enrichment, the y-axis lists enriched biological processes, bubble size represents the number of differentially expressed genes associated with each term, and bubble color indicates the adjusted *q* value. Analyses included four independent fibroblast isolations per group.

# Supplementary Figure 5

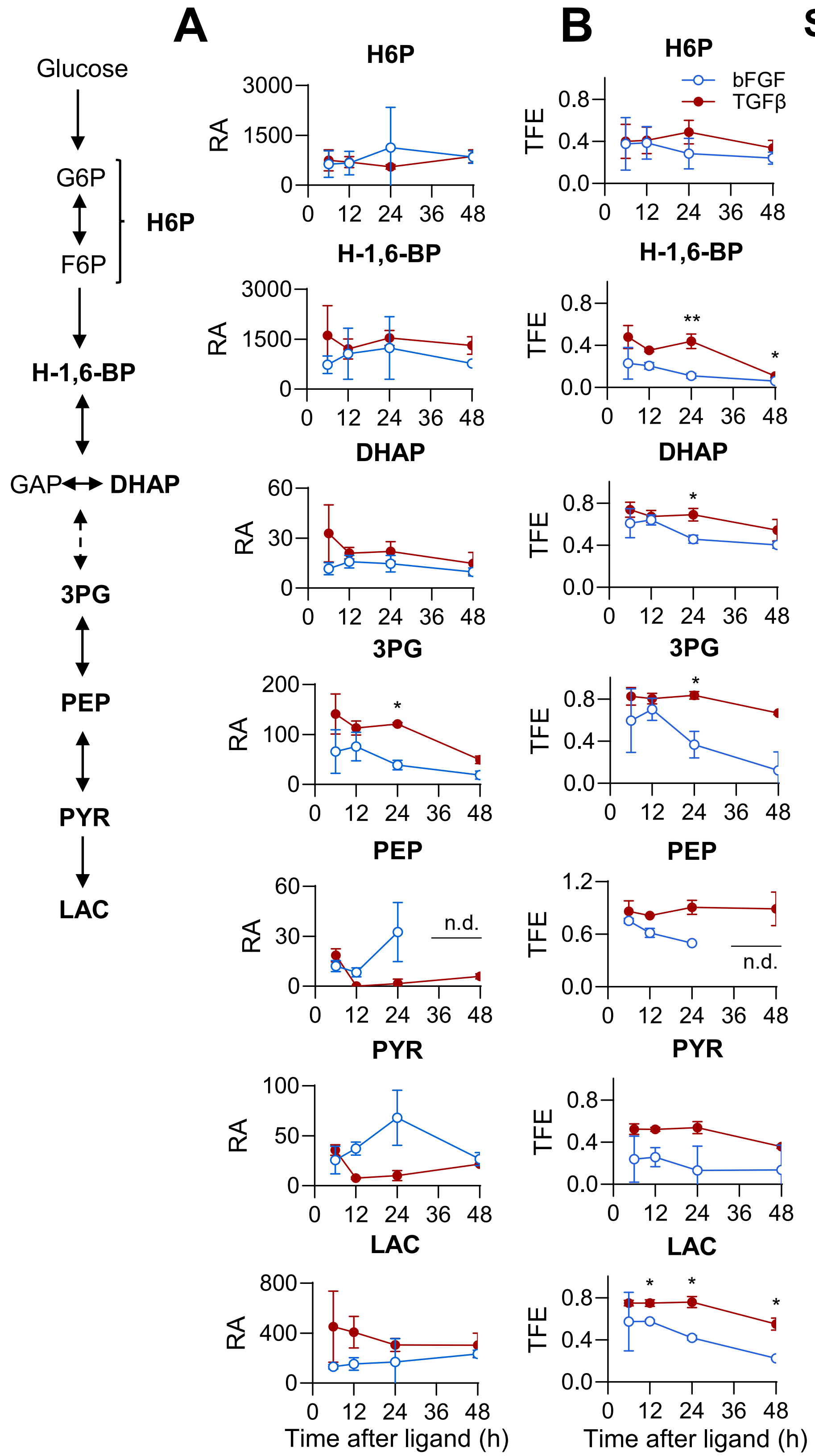


**Supplementary Figure 5. Relative abundance and total $^{13}$C enrichment of glycolytic metabolites.** Primary cardiac fibroblasts isolated from adult male mice were treated with bFGF or TGFβ for 6–48 h, with glucose replaced by [U-$^{13}C_6$]-glucose during the final 6 h of treatment. Polar metabolites were extracted for stable isotope-resolved metabolomics. The pathway schematic identifies the glycolytic metabolites analyzed in bold. **(A)** Protein-normalized relative abundance and **(B)** total fractional $^{13}$C enrichment (TFE) of glycolytic metabolites. Relative abundance (RA) data were normalized to total protein, and TFE values were logit transformed before statistical analysis. Data are mean ± SD from three independent fibroblast isolations, each obtained from a different male mouse. Statistical comparisons were performed by two-way repeated measures ANOVA followed by Šídák's multiple-comparisons test. Metabolites not detected at a given time point are indicated by "n.d." H6P, hexose 6-phosphate; H-1,6-BP, hexose 1,6-bisphosphate; DHAP, dihydroxyacetone phosphate; 3PG, 3-phosphoglycerate; PEP, phosphoenolpyruvate; PYR, pyruvate; LAC, lactate. *$p < 0.05$; **$p < 0.001$.

# Supplementary Figure 6

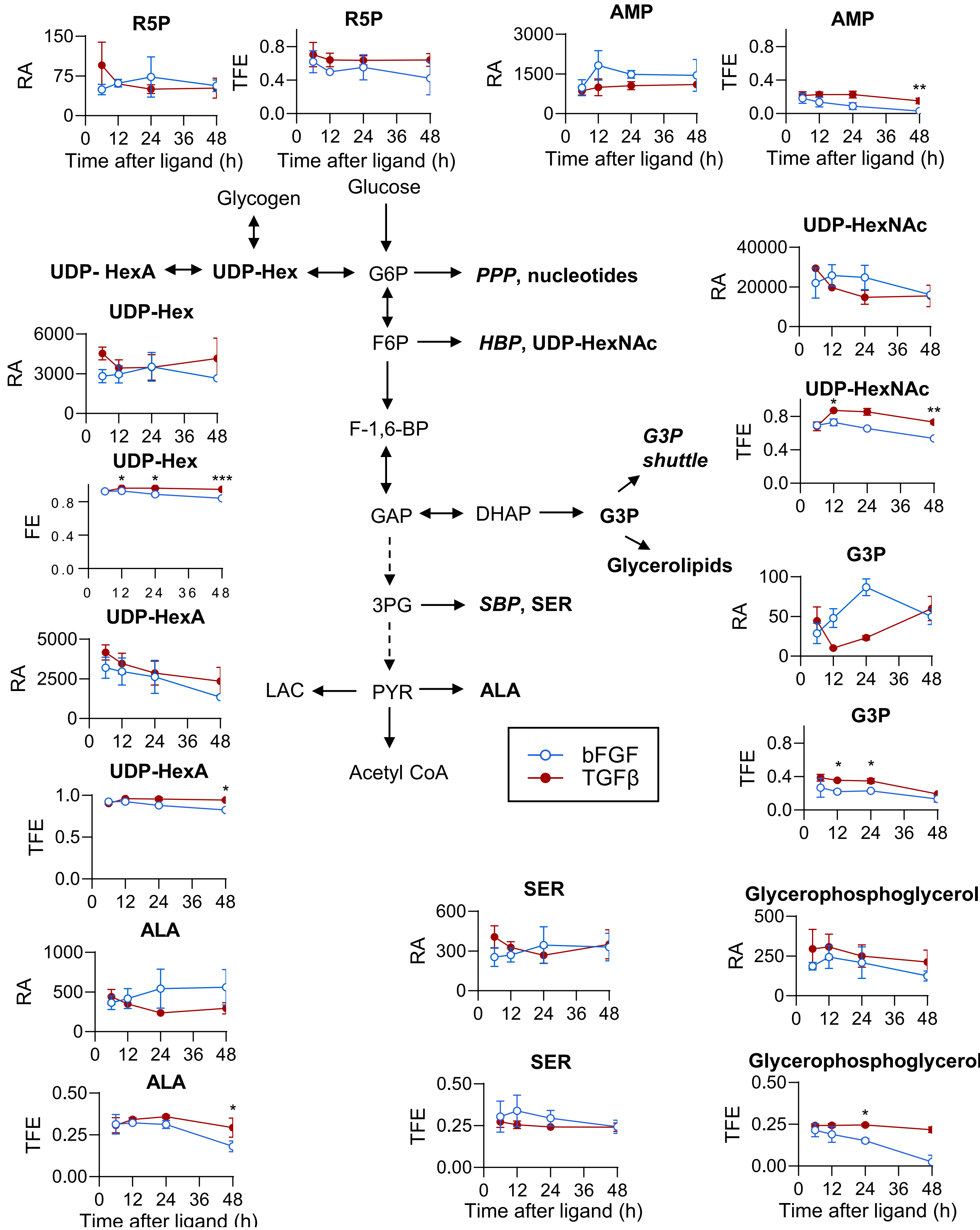


**Supplementary Figure 6. Relative abundance and total $^{13}C$ enrichment of metabolites in biosynthetic pathways branching from glycolysis.** Primary cardiac fibroblasts isolated from adult male mice were treated with bFGF or TGFβ for 6–48 h, with glucose replaced by [U-$^{13}C_6$]glucose during the final 6 h of treatment. Metabolites were extracted for stable isotope-resolved metabolomics. The central schematic depicts biosynthetic pathways branching from glycolysis, with analyzed metabolites shown in bold. Shown are protein-normalized relative abundance (RA) and total fractional $^{13}C$ enrichment (TFE) for metabolites in the pentose

## Supplementary Figure 7

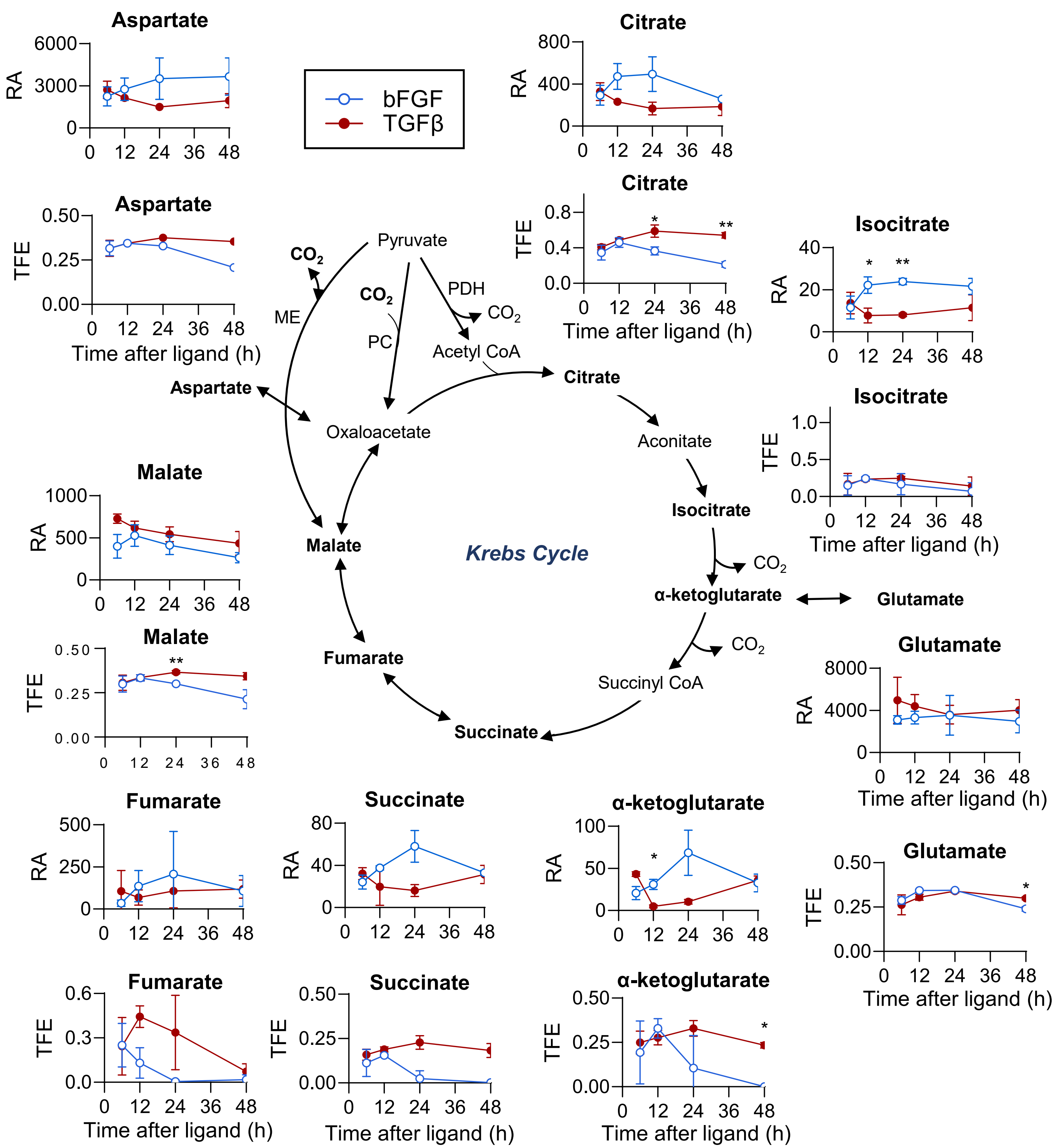


**Supplementary Figure 7. TGFβ increases glucose-derived labeling of Krebs cycle intermediates at later time points.** Primary cardiac fibroblasts isolated from adult male mice were treated with bFGF or TGFβ for 6–48 h, with glucose replaced by [U-$^{13}C_6$]-glucose during the final 6 h of treatment. Metabolites were extracted for stable isotope-resolved metabolomics. The central schematic depicts the Krebs cycle and associated routes of pyruvate entry and exchange, with analyzed metabolites shown in bold. Shown are protein-normalized relative abundance (RA) and total fractional $^{13}C$ enrichment (TFE) of Krebs cycle intermediates and associated metabolites. TFE values were logit transformed before statistical analysis. Data are mean ± SD from three independent fibroblast isolations, each obtained from a different male mouse. Statistical comparisons were performed by two-way repeated-measures ANOVA followed by Šídák's multiple-comparisons test. PDH, pyruvate dehydrogenase; PC, pyruvate carboxylase; ME, malic enzyme. *$p < 0.05$; **$p < 0.001$.

# Supplementary Fig. 8

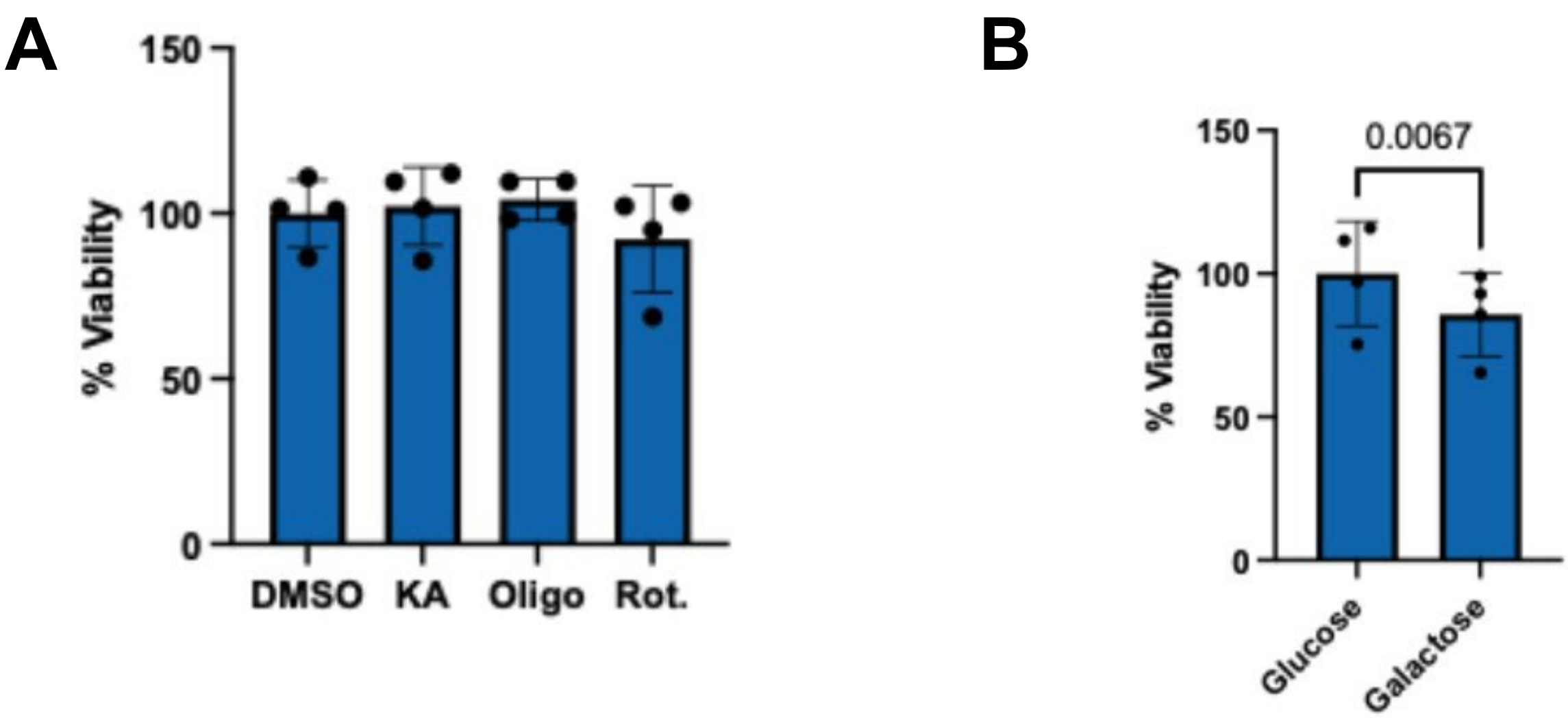


**Supplementary Figure 8. Metabolic interventions do not reduce cell viability in cardiac fibroblasts.** Primary cardiac fibroblasts isolated from adult male mice were treated for 48 h with vehicle, koningic acid (KA; 20 μM), oligomycin (Oligo; 1 μM), rotenone (Rot; 1 μM), or medium containing glucose or galactose as the principal carbohydrate substrate. Cell viability relative to vehicle control was calculated as the intracellular LDH activity in the cell lysate divided by total LDH activity (conditioned medium plus cell lysate), normalized to the corresponding vehicle control and multiplied by 100. (A) Relative cell viability following treatment with vehicle (DMSO), KA, oligomycin, or rotenone. (B) Relative cell viability of fibroblasts cultured in glucose- or galactose-containing medium. Data are mean ± SD from four independent fibroblast isolations, each obtained from a different male mouse. Comparisons in A were performed by one-way ANOVA followed by Šídák's multiple-comparisons test. Comparisons in B were performed by a paired t-test.

# Supplementary Fig. 9

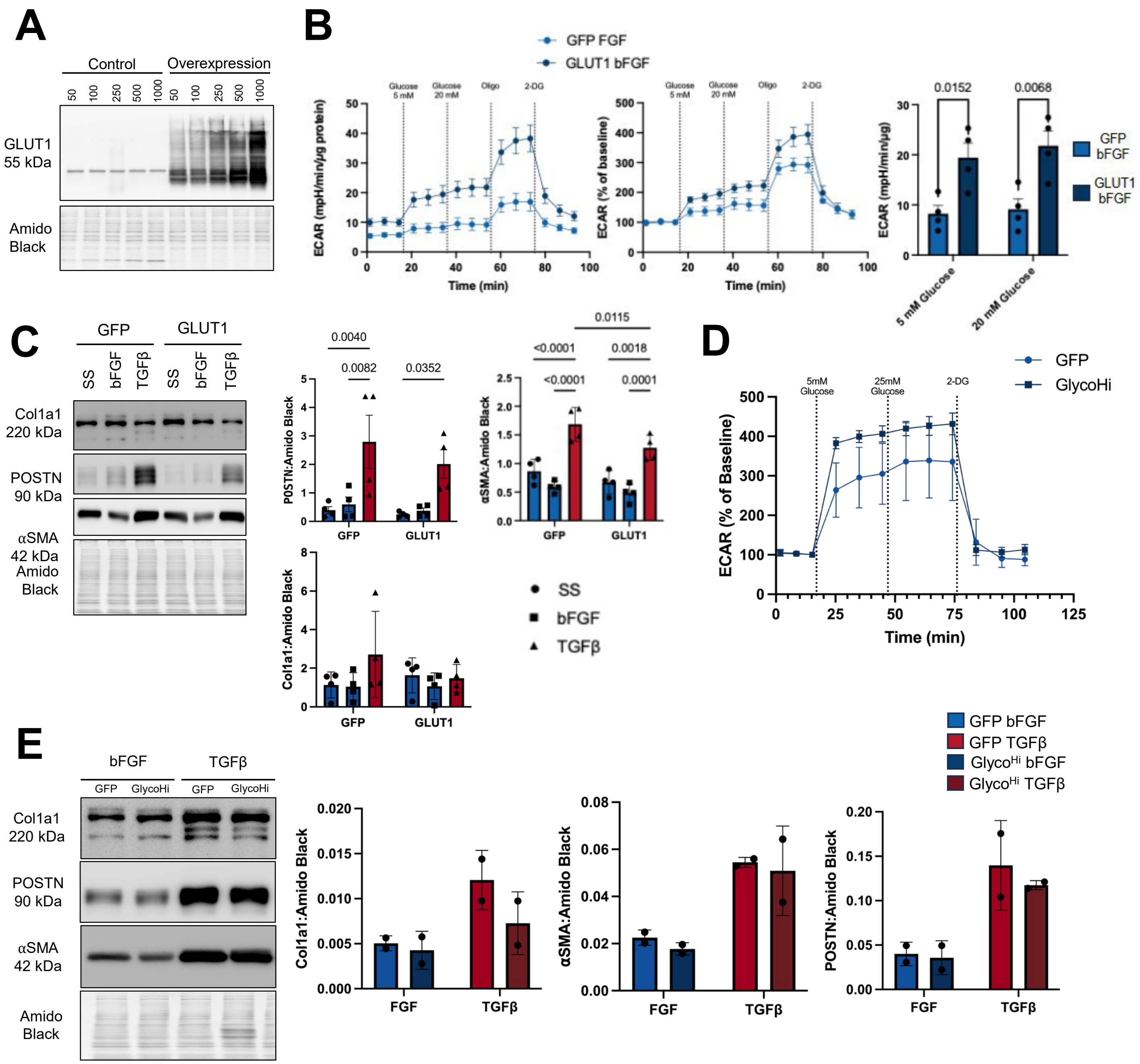


**Supplementary Figure 9. GLUT1 overexpression increases glycolytic activity but is insufficient to induce myofibroblast-marker expression. (A)** Primary cardiac fibroblasts were transduced with control Ad-GFP or Ad-SLC2A1 at multiplicities of infection (MOIs) of 50, 100, 250, 500, or 1000 for 24 h, followed by a 72-h recovery period. **(B, C)** Cardiac fibroblasts were transduced with Ad-GFP or Ad-SLC2A1 (GLUT1) at an MOI of 500. **(B)** ECAR and OCR following sequential addition of glucose to final concentrations of 5 and 20 mM, followed by oligomycin and 2-deoxy-D-glucose after 48 h treatment with bFGF. **(C)** Representative immunoblots and densitometric quantification of cardiac fibroblasts maintained under serum starved conditions (SS) or treated with bFGF or TGFβ for 48 h following immunoblotting for periostin (POSTN), α-smooth muscle actin (αSMA), and collagen (Col1a1). Densitometric values were normalized to total lane protein visualized by amido black staining. **(D)** Primary cardiac fibroblasts were transduced with control Ad-GFP or Ad-pd-PFK2 (Glyco$^{Hi}$) at MOI of 500 for 24 h, followed by a 24-h recovery period. ECAR following sequential addition of glucose to final concentrations of 5 and 25 mM, followed by 2-deoxy-D-glucose. An MOI of 500 was selected for subsequent experiments. n = 1 with technical replicates. **(E)** Representative immunoblots and densitometric quantification of cardiac fibroblasts treated with bFGF or TGFβ for 48 h following immunoblotting for periostin (POSTN), α-smooth muscle actin (αSMA), and collagen (Col1a1). Densitometric values were normalized to total lane protein visualized by amido black staining. Panel A , D, and E represent an optimization experiment from one (A,D) or two (E) fibroblast isolations; no inferential statistics were performed because of the low n. Data in B and C (right panels) are mean ± SD from four independent fibroblast isolations, each obtained from a different male mouse. Statistical comparisons were performed by two-way ANOVA followed by Šídák's multiple-comparisons test.
